# Electrochemical Trifluoromethylation of Enamides under Microflow Conditions

**DOI:** 10.1021/acs.oprd.4c00311

**Published:** 2024-10-22

**Authors:** Anna Vanluchene, Tomas Horsten, Eli Bonneure, Christian V. Stevens

**Affiliations:** Department of Green Chemistry and Technology, Faculty of Bioscience Engineering, Ghent University, Coupure Links 653, 9000 Ghent, Belgium

**Keywords:** electrochemistry, trifluoromethylation, flow electrolysis, enamides

## Abstract

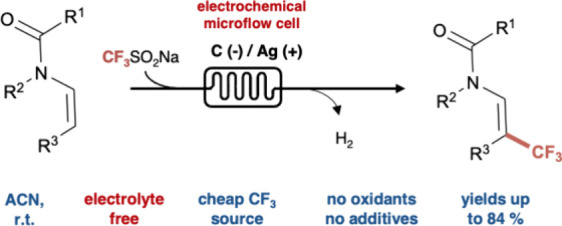

The development of sustainable trifluoromethylations of enamides is of great interest to the pharmaceutical industry. Herein, we demonstrate a sustainable direct electrochemical trifluoromethylation method in a microflow cell, using Langlois reagent, without the need for a supporting electrolyte, oxidants, or any additive under mild conditions. This method can be applied to various substrates with a yield of up to 84%. Additionally, the batch process yielded significantly less (22%), highlighting the microflow cell’s efficiency.

## Introduction

**Scheme 1:**
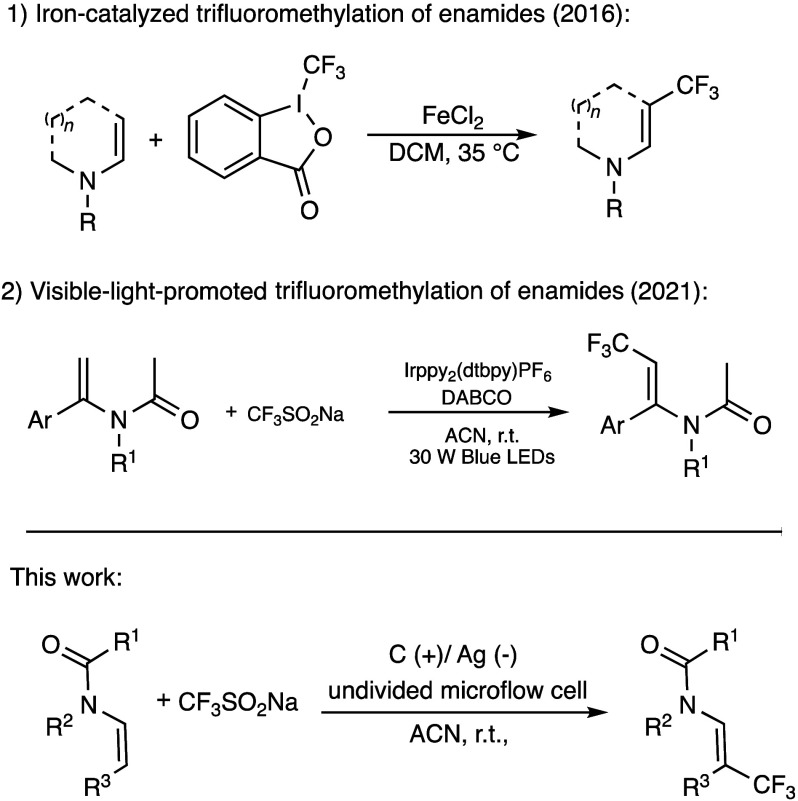
Recent Representative Examples of Trifluoromethylation of Enamides

The synthesis of trifluoromethylated compounds is a long-standing objective in organic chemistry due to their significant applications in agrochemicals and pharmaceuticals, comprising about 20% of the marketed drugs.^1−8^ The introduction of fluorinated groups can enhance the drug’s acidity, lipophilicity, and metabolic stability.^9^ Additionally, functionalized enamides, found in numerous natural products^10^ and pharmaceuticals,^11,12^ are key building blocks for complex amino derivatives.^13−16^ They offer greater stability and reduced reactivity compared to enamines due to the electron-withdrawing *N*-acyl group.^13,15,17^ Several approaches to synthesize trifluoromethylated enamides are documented in the literature. Togni’s reagent was used in combination with copper catalysis by Feng and Loh,^18^ and with iron catalysis by Gillaizeau's group (Scheme 1).^19^ Masson's group demonstrated photoredox oxytrifluoromethylation of enecarbamates using Togni’s reagent in the presence of a photocatalyst.^20^ Yang's group developed visible-light-promoted olefinic trifluoromethylation of enamides by Langlois reagent in the presence of a photocatalyst and DABCO (Scheme 1).^21^ Yu's group introduced visible-light-promoted trifluoromethylation using Umemoto reagent in the presence of a photocatalyst,^22^ and without photocatalyst in the presence of a base.^11^ Nevertheless, all these methods employed high-cost or unstable trifluoromethylating reagents, additional metal catalysts, photocatalysts, or oxidants. Thus, alternative methods toward direct trifluoromethylation of enamides are desirable, with an electrochemical approach being particularly promising.^23^ A series of significant trifluoromethylation methodologies using electrochemistry^24^ and Langlois reagent (CF_3_SO_2_Na) has been developed, for instance trifluoromethylation of unactivated alkenes,^25^ quinolinones,^26^ 2-pyridones,^27^ and allylic alcohols.^28^ However, direct trifluoromethylation of enamides remains challenging. Interestingly, a double functionalization of enamides is more feasible when the trifluoromethylated carbocation intermediate is attacked by a nucleophile.^29−31^ For instance, in the presence of water, an oxytrifluoromethylation product can be obtained,^32^ and with an alcohol, the corresponding alkoxytrifluoromethylation product can be formed.^33^ Luo's group demonstrated the difficulty of direct trifluoromethylation with a method involving H_2_SO_4_ in DMF in an electrochemical batch reactor and based on *N*-benzyl-*N*-(1-phenylvinyl)acetamides.^34^ Earlier this year, Chausset-Boissarie's group reported the trifluoromethylation of enamides by Langlois reagent with a supporting electrolyte with poor yields and a limited scope.^33^

The challenges stem from Langlois reagent, and the formed sodium salts limited solubility in the organic solvents, which hinder the double functionalization and have a large electrochemical potential window, such as ACN. This results in a decrease in yield, especially due to electrode passivation when no additional solvent, e.g., MeOH or water, is used. We present a straightforward method for the direct trifluoromethylation of enamides in a microflow cell, eliminating the need for any additives. This method enhances the process, leading to higher yields compared with a batch reactor. This approach reduces waste and simplifies product purification, making electrolyte-free electrochemical synthesis highly desirable yet underexplored.^27,35−39^

## Results and Discussion

We began our study with model substrate **1a** and Langlois reagent (**2**), aiming to optimize the reaction conditions in an undivided microflow cell (Table 1). The conditions were as follows: an initial reaction in ACN with 3 equiv of **2**, graphite electrodes, a constant current (cc) of 10 mA (*j* = 1.5 mA/cm^2^), and a flow rate of 155 μL/min, corresponding to a residence time of 52 s and an applied charge of 2 F, resulting in a moderate yield (entry 1), which also proved to be an optimal production rate (for detailed information, see 1). Adding a supporting electrolyte (LiClO_4_, entry 2) reduced the yield. Increasing the channel height (larger interelectrode distance) compared to the initial (54 vs 127 μm), while keeping the applied charge constant, slightly decreased the yield (entry 3). Since **2** precipitated in the syringe, we next used 2 equiv of **2** (entry 4), which led to an increase in yield. Further decreasing the equivalents of **2** resulted in a lower yield (entries 5 and 6). Increasing the applied charge to 2.5 F (entry 7) did not affect the yield, while a higher applied charge of 3 F (entry 8) decreased the yield, as did applying a lower charge of 1.5 F (entry 9). Evaluating various solvents showed that in HFIP (entry 7), only trace amounts of the product were detected, while other solvent mixtures (entry 8–11) also led to yield reduction and the formation of several side products. The reaction proceeded the best in ACN, although electrode fouling was observed on the cathode (see 1). Alternating and rapid alternating polarity were evaluated as strategies to prevent fouling.^40^ Although these methods reduced electrode fouling, as measured by electrode weights before and after the reaction (see Table S4 for details), yields dropped significantly, with mostly starting material present in the reaction mixture (entry 15–17). This drop in yield is likely due to the high capacitance of graphites, where electricity is consumed by charge–discharge cycles of the electrical double layer.^40,41^ Longer pulses and higher currents only slightly improved the yields (entries 18 and 19). Additionally, different cathodes were tested: stainless steel (SS, entry 23) and silver (Ag, entry 24). To our delight, using a silver electrode, the reaction yield increased to 82% with less fouling (8.5 vs 21.4 mg for carbon). Adding a back-pressure regulator (since gas is produced) significantly reduced the yield. No reaction occurred without electricity. Different channel geometries did not significantly affect the yield compared to a simple channel design (see 2).

**Table 1:**
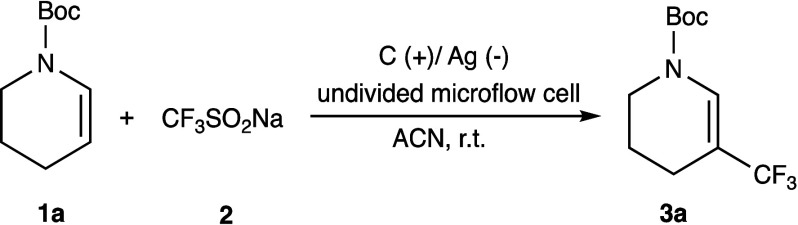
Selected Optimization Data for the Electrochemical Trifluoromethylation of Enamide **1a** to Product **3a**^a^

entry	equiv of **2a**	solvent	current density [mA/cm^2^]	anode/cathode	applied charge [F]	yield^b^ [%]
1	3	ACN	1.5	C/C	2	65
2^c^	3	ACN	1.5	C/C	2	47
3^d^	3	ACN	1.5	C/C	2	54
4	2	ACN	1.5	C/C	2	74
5	1.5	ACN	1.5	C/C	2	63
6	1.1	ACN	1.5	C/C	2	38
7	2	ACN	1.8	C/C	2.5	77
8	2	ACN	2.2	C/C	3	51
9	2	ACN	1.1	C/C	1.5	54
10	2	HFIP	1.5	C/C	2	3
11	2	MeOH	1.5	C/C	2	9
12	2	ACN:MeOH 4:1	1.5	C/C	2	17
13	2	ACN:H_2_O 4:1	1.5	C/C	2	8
14	2	ACN:H_2_O 1:1	1.5	C/C	2	3
15^e^	2	ACN	1.5	C/C	2	9
16^f^	2	ACN	1.5	C/C	2	9
17^g^	2	ACN	1.5	C/C	2	22
18^g^	2	ACN	2.9	C/C	4	14
19^h^	2	ACN	2.9	C/C	4	0
20	2	ACN	1.5	C/SS	2	68
21	2	ACN	1.5	C/Ag	2	82
22^i^	2	ACN	1.5	C/Ag	2	56
23^j^	2	ACN	0	C/Ag	0	0

a Reaction conditions in an undivided flow electrolysis cell (Analytical Sales, Inc.): **1a** (0.2 mmol, 1 equiv), **2a** (1.1–3 equiv), solvent (10 mL), plate electrodes, current, RT, flow rate 155 μL/min (corresponds to residence time 52 s) for 65 min (time to convert 0.2 mmol).

b Yield was evaluated by ^19^F-NMR spectroscopy of the crude reaction mixture using trifluorotoluene as an internal standard.

c Supporting electrolyte LiClO_4_ (0.1 M).

d Channel height 254 μm instead of 127 μm. Alternating polarity.

e 0.2 Hz.

f 0.05 Hz.

g 0.01 Hz.

h Rapid alternating polarity, 10 Hz.

i With back pressure regulator.

j No current.

**Table 2:** Comparison of Trifluoromethylation of Enamides in the Batch Reactor and Microflow Cell

process	yield [%]	current density [mA/cm^2^]	current [mA]	potential [V]	applied charge [F]
flow	82	1.5	10	4	2
batch	22	1.5	5	7	2
batch	51	3.0	10	11	2

**Figure 1:**
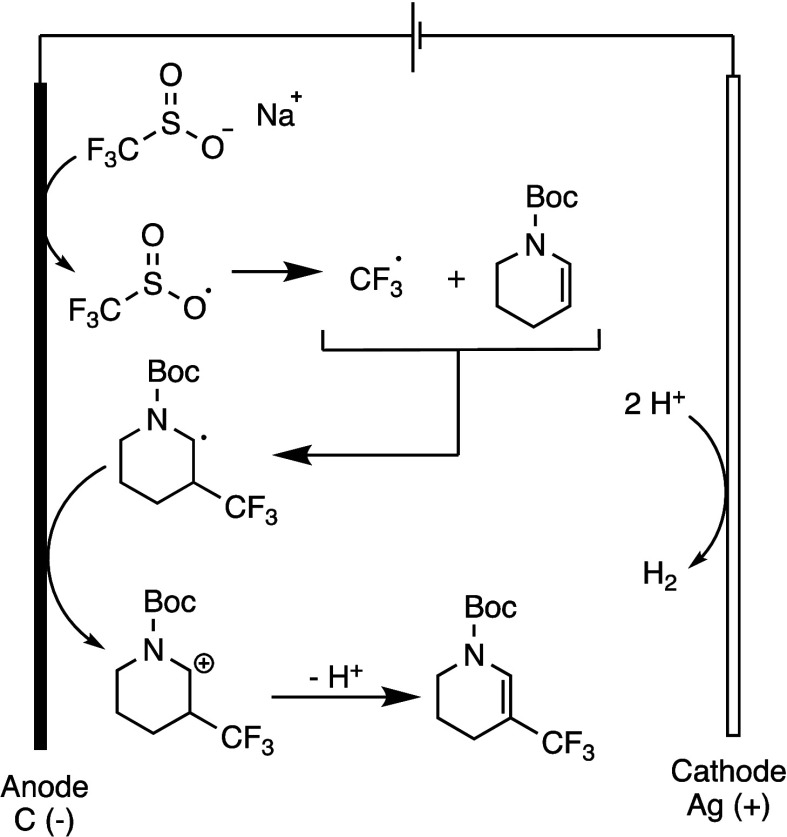
Proposed reaction mechanism.

The microflow cell and batch process were compared (Table 2) under the same optimized reaction conditions (0.2 mmol of substrate, 2 equiv. CF_3_SO_2_Na, 10 mL of ACN, C(+)/Ag(−), 2 F, and current density of 1.5 mA/cm^2^). In the batch reactor, a current of 5 mA was used, compared to 10 mA in the flow reactor, to achieve the same current density due to the double surface area of the electrodes. The batch process resulted in a yield of just 22%. The improved yield in the microflow cell, achieved under the same conditions, can be attributed to the greater process intensification due to a high surface-area-to-volume ratio. Increasing the current density in the batch reactor raised the yield to 51%, which is still significantly lower than 82% achieved in the flow reactor. Additionally, the batch reactor experienced high overpotential, unlike the flow reactor, due to the small interelectrode distance and the constant introduction of fresh reaction mixture, where the Langlois reagent provides conductivity. Several supporting electrolytes, including LiClO_4_, Bu_4_NClO_4_, TBAPF_6_, Et_4_NI, and Et_4_NBF_4_ (0.1 M), were tested in the batch reactor to reduce the overpotential, but their presence led to decreased yields (for details see Table S3).

**Scheme 2:**
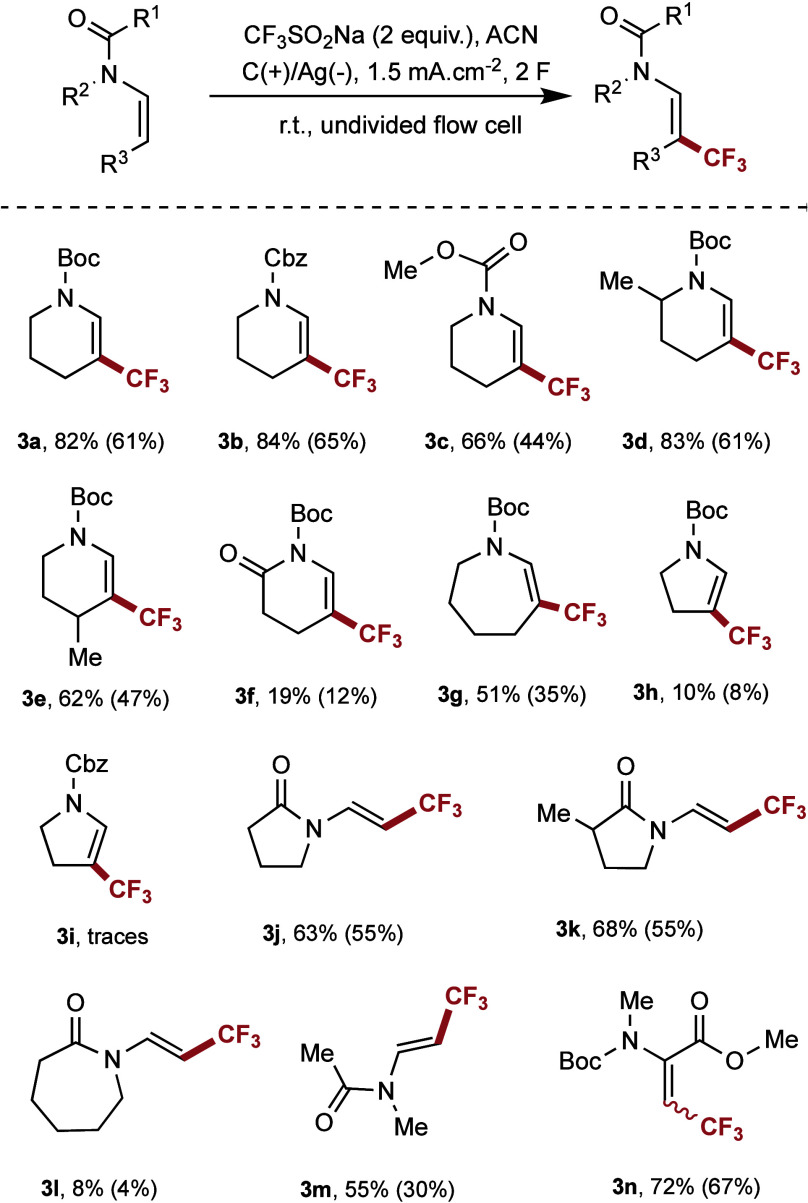
Electrochemical Synthesis of Trifluoromethylated Enamides in the Flow Cell Reaction conditions: **1** (0.2 mmol, 1 equiv), CF_3_SO_2_Na (0.2 mmol), ACN (10 mL), electrochemical flow cell (Analytical Sales Inc.), PFA simple channel (*h* = 127 μm), graphite plate anode (50 × 50 × 3 mm), silver plate anode (50 × 50 × 1 mm), constant current 10 mA (*j* = 1.5 mA/cm^2^), flow rate 155 μL/h (2 F/mol) at room temperature. The yield was evaluated by ^19^F-NMR spectroscopy of the crude reaction mixture using trifluorotoluene as internal standard. Yields reported in brackets refer to isolated and purified products.

Having established the optimized reaction conditions, the substrate scope of the trifluoromethylation reaction of enamides was explored. As shown in Scheme 2, various substrates, including carbamates and endocyclic, exocyclic, and acyclic enamides, were tested. The effect of different protecting groups was evaluated; *N*-Boc- and *N*-Cbz-protected substrates led to similar yields (**3a**, **3b**), while the methoxycarbonyl-protected substrate led to a slightly lower yield (**3c**). Next, the effect of various substituents was examined. For the enamide with a methyl group (electron donating) in the 4-position (**3e**), the desired product was obtained in a slightly lower yield than that with a methyl group in the 2-position (**3d**), though the yield remained above 80%, likely due to steric hindrance. Considerably lower yields were observed for **3f**, likely due to the lower affinity of CF_3_ radicals to electron deficient substrates. The ring size had a significant effect on the reaction' success. A moderate yield was obtained for the seven-membered ring (**3g**), while poor yields were obtained with five-membered rings (**3h** to electron deficient substrates. The ring size had a significant effect on the reaction's success: a moderate yield was obtained for the seven-membered ring (3g), while poor yields were observed for five-membered rings (3h, 3i). Cyclic voltammetry showed that the oxidation potential of the five-membered ring substrates is lower than that of the Langlois reagent (Ep_1/2_ = 1.11 V compared to Ep_1/2_ = 1.17 V, vs Ag/AgCl), leading to the primary oxidation of the substrate and resulting in a large number of side products. To our delight, good yields were obtained for endocyclic five-membered enamides (**3j**, **3k**), but a significant drop in yield was observed for the seven-membered endocyclic enamide (**3l**). Additionally, good yields were obtained for acyclic enamides (**3m**, **3n**). In summary, several new trifluoromethylated enamides (**3c**, **3d**, **3e**, **3g**, **3k**, **3m**, and **3n**) were obtained in good yields.

Cyclic voltammetry experiments were conducted to gain further insight into the mechanism. Enamide (**1a**) showed a higher oxidation potential than CF_3_SO_2_Na (Ep_1/2_ = 1.21 V compared to Ep_1/2_ = 1.17 V, vs Ag/AgCl), indicating that CF_3_SO_2_Na is more easily oxidized at the anode. A plausible reaction mechanism for the electrochemical trifluoromethylation of enamide is illustrated in Figure 1. Initially, the CF_3_ radical is generated from CF_3_SO_2_Na by anodic oxidation with the release of SO_2_. The addition of this radical to the enamide leads to the formation of a carbon radical intermediate. This intermediate is further oxidized at the anode to form a carbon cation, which, after the loss of a proton, results in the desired trifluoromethylated product. As a counter reaction, protons are reduced to hydrogen gas at the cathode. This mechanism is consistent with previous literature reports.^42^

**Figure 2:**
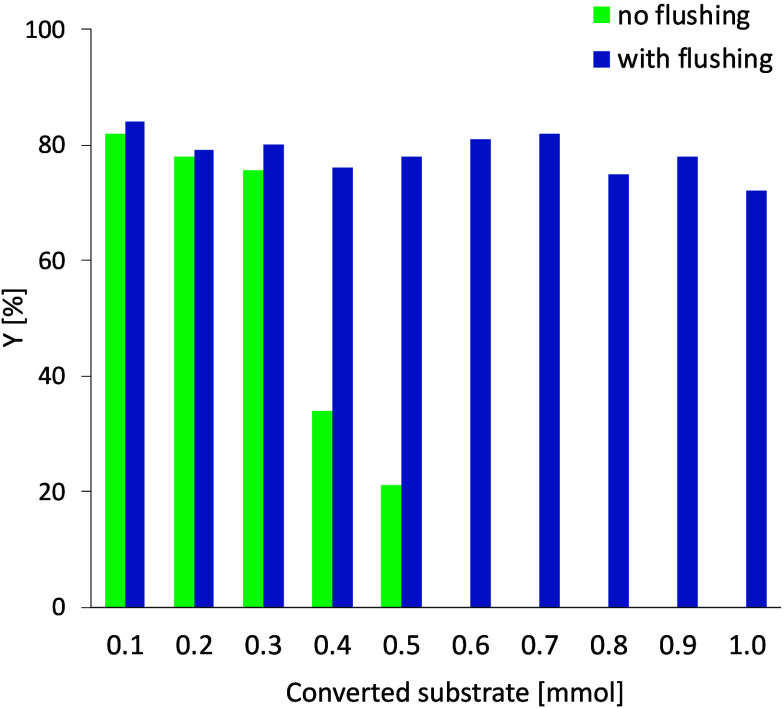
Stability of the production of trifluoromethylated product over time: comparing the microflow reactor performance without (green) and with (blue) a flushing step, every 0.2 mmol (1 h). Yields were evaluated by ^19^F-NMR spectroscopy of the crude reaction mixture using trifluorotoluene as an internal standard.

Despite, the fouling of electrodes, a high yield of the desired product **3a** was observed (82%) on a 0.2 mmol scale, which is comparable with scale in recent literature on trifluoromethylation functionalization with Langlois reagent, especially in the case of batch reactors.^11,21,30,33^ However, electrode fouling can negatively impact the yield during prolonged production. To address this often-overlooked issue, the stability of the yield over time was evaluated. Eventually, the yield decreases due to electrode passivation. After 3 h (corresponding to conversion of 0.6 mmol of substrate), almost no product was synthesized (Figure 2). Electrode fouling is caused by Na^+^ salts generated during the reaction with the Langlois reagent, which have limited solubility in ACN but are well soluble in water. The flow setup allows for a continuous processing with an incorporated flushing step. When the microflow cell was flushed with water, no decrease in yield was observed. Specifically, after 1 h of production (conversion of 0.2 mmol of substrate), the flow cell was flushed with 10 mL of H_2_O (5 mL/min for 2 min). Afterward, the reaction mixture was introduced again, and after five residence times (4.5 min, to ensure the absence of water and prevent side product formation), the reaction mixture was collected again. As the system is flushed by the reaction mixture for one washing step, 0.014 mmol of substrate is consumed, and the overall yield of the process is 73%. Additionally, the flow cell was disassembled after the flushing, and no electrode fouling was observed (see Section S5 for details). The valves to switch from the flow stream of the reaction mixture or water to flush the microflow cell were operated manually; however, this process can be automated (with programmable valves and pumps) for extended production of the desired product.

## Conclusions

In conclusion, an environmentally friendly method for direct electrochemical trifluoromethylation of enamides was developed using a microflow cell and Langlois reagent as the source of the trifluoromethyl radical, without the need for a supporting electrolyte. Various new trifluoromethyl enamides were produced in moderate to good yields. We demonstrated that this method is feasible exclusively in the microflow cell, and not in the batch reactor, for several reasons: (i) the microflow cell allows operation without a supporting electrolyte (which otherwise reduces the yield) due to the small interelectrode distance, resulting in a low Ohmic drop; (ii) process intensification, due to a high electrode surface-area-to-volume ratio, increases the yield in flow; (iii) the microflow cell can be easily flushed with water to prevent electrode fouling, allowing for continuous production without a yield drop, a feature unattainable in a batch reactor.

## Experimental Section

### General Considerations

Chemicals were purchased from Sigma-Aldrich, Fluorochem, Abcr, and Enamine and used as received. Technical-grade solvents were used for column chromatography. Column chromatography was performed via automatic flash chromatography on a Büchi Reveleris X2 flash chromatography system. Reusable columns (SiO_2_, particle size of 0.040–0.063 mm) were used for the purification of the crude products. The effluent was analyzed by an evaporative light scattering detector (ELSD) and three ultraviolet detectors, of which the wavelengths were adjusted depending on the mixture to be purified. Nuclear magnetic resonance ^1^H NMR, ^13^C NMR, and ^19^F-NMR spectra were recorded at 400.1, 100.6, and 376.5 MHz, respectively, in CDCl_3_ with TMS as the internal chemical shift standard, using a Bruker Avance III HD 400 spectrometer equipped with a 1H/BB z-gradient probe (BBO, 5 mm). All spectra were processed by using TopSpin 4.0.8. IR spectra were obtained from samples in neat form with a Quest ATR accessory with a diamond crystal puck using a Shimadzu IRAffinity-1S Fourier transform infrared spectrophotometer (FTIR). UPLC analysis was performed using a Vanquish UPLC system (Thermo Scientific) with a mobile phase ACN containing 0.1% formic acid. The injection volume was set to 2 μL, and the column temperature was maintained at 30 °C. A Kinetex C18 50 mm × 2.1 mm × 2.6 μm column (Phenomenex) was used for seperation. High-resolution mass spectrometry (HRMS) was recorded on a Q Exactive Plus (Orbitrap, Thermo Scientific), with the following specifications: MS method—resolution 140,000; AGC Target 1e6; Max It: 50 ms; scan range: 150–500 *m*/*z*; using both positive and negative ESI.

### General Procedure in the Microflow Cell

A flow electrolysis undivided cell with a PFA simple channel with a height of *h* = 127 μm, graphite plate anode (50 × 50 × 3 mm), and silver plate cathode (50 × 50 × 1 mm) was assembled. The flow cell, the PFA simple channel with a surface area of *a* = 681 mm^2^, and the graphite anode were purchased at Analytical Sales and Services, Inc., and the Ag plate was purchased at Fisher Scientific. The carbon plate anode was aligned by silicone rubber alignment gaskets, and for the Ag plate cathode, homemade alignment gaskets made from PTFE foil were used, since the Ag plate cathode is thinner (1 mm) compared to the graphite plate cathode. The microflow cell was connected with a power supply (Axiomet AX-3003P). Langlois reagent, CF_3_SO_2_Na (0.4 mmol, 62 mg, 2 equiv), was dissolved in 10 mL of ACN; afterward, 0.2 mmol (37 mg) of substrate was added. The reaction mixture was then transferred to a 10 mL Luer-Lock syringe and pumped through the microflow cell by a syringe pump at a flow rate of *Q* = 155 μL/h, corresponding to a residence time of *t*_R_ = 52 s (the reactor volume was *V* = 0.134 mL). The cell was operated under a constant current of *I* = 10 mA (current density of *j* = 1.6 mA/cm^2^), resulting in voltage (*U*) from 4 to 5 V. This setting results in applied charge (*q*) of 2 F relative to substrate **1a** (*n*_A_). The residence time *t*_R_, flow rate *Q*, and current density *j* were calculated based on eqs 1, 2, and 3, respectively. When the reactor reached the steady state (3 min), the reaction mixture was collected. For optimization of the procedure, the yield was evaluated by quantitative F-NMR (32 scans, *t*_relaxation_ = 30 s). The reaction mixture was concentrated under reduced pressure, and the internal standard (trifluorotoluene) was added. Otherwise, the reaction mixture was extracted with Et_2_O and H_2_O two times. The organic phases were collected and dried with anhydrous MgSO_4_. The solvent was removed, and the crude product was purified by flash silica gel column chromatography using the eluent indicated in each case to give the final trifluoromethylated product.
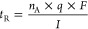
1
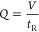
2
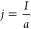
3

### General Reaction Procedure in Batch

To a 10 mL vial (Ika ElectraSyn 2.0, Ident. No.: 0020008980) equipped with a stirring bar were added CF_3_SO_2_Na (0.4 mmol, 2 equiv), supporting electrolyte (0.1M), ACN (10 mL), and enamide (0.2 mmol, 1 equiv). IKA Graphite SK-50 (Ident. No.: 0040002858) as the anode and IKA Silver plated as the cathode (Ident. No.: 0040002854) with dimensions of 8 mm × 52.5 mm × 2 mm were used in the electrolysis. The reaction was run at constant current (10 mA) and applied charge of 2 F. After completion of the reaction, the crude reaction mixture was concentrated under reduced pressure and the internal standard (trifluorotoluene) was added. For optimization of the procedure, the yield was evaluated by quantitative F-NMR (32 scans, *t*_relaxation_ = 30 s). Afterward, the electrodes were washed by sonicating in acetone followed by washing in water to remove the buildup. If impurities were still present on the electrode surface, then the electrode surface was cleaned by fine sandpaper.

## Data Availability

https://zenodo.org/records/13959448.

## Supporting Information Available

Experimental details and compound characterization data (DOCX)

## References

[ref1] Cantillo D.; Kappe C. O. Halogenation of organic compounds using continuous flow and microreactor technology. React. Chem. Eng. 2017, 2 (1), 7–19. 10.1039/C6RE00186F.

[ref2] Champagne P. A.; Desroches J.; Hamel J. D.; Vandamme M.; Paquin J. F. Monofluorination of Organic Compounds: 10 Years of Innovation. Chem. Rev. 2015, 115 (17), 9073–9174. 10.1021/cr500706a. 25854146

[ref3] Atobe M.; Tateno H.; Matsumura Y. Applications of Flow Microreactors in Electrosynthetic Processes. Chem. Rev. 2018, 118 (9), 4541–4572. 10.1021/acs.chemrev.7b00353. 28885826

[ref4] Allegrini G.; Di Desidero T.; Barletta M. T.; Fioravanti A.; Orlandi P.; Canu B.; Chericoni S.; Loupakis F.; Di Paolo A.; Masi G.; et al. Clinical, pharmacokinetic and pharmacodynamic evaluations of metronomic UFT and cyclophosphamide plus celecoxib in patients with advanced refractory gastrointestinal cancers. Angiogenesis 2012, 15 (2), 275–286. 10.1007/s10456-012-9260-6. 22382585 PMC3338912

[ref5] Karbwang J.; White N. J. Clinical Pharmacokinetics of Mefloquine. Clin Pharmacokinet 1990, 19 (4), 264–279. 10.2165/00003088-199019040-00002. 2208897

[ref6] Plosker G. L.; Figgitt D. P. Tipranavir. Drugs 2003, 63 (15), 1611–1618. 10.2165/00003495-200363150-00009. 12887268

[ref7] Marshall G.; Burns F.; Akodu J.; Hunter A.; Barber T. A retrospective review to identify acceptability of doravirine-containing regimens in our single-centre cohort of people with HIV (PWH). HIV Med. 2023, 24, 25–26.

[ref8] Kim N.; Patrick L.; Mair S.; Stevens L.; Ford G.; Birks V.; Lee S. H. Absorption, metabolism and excretion of [ 14 C]gemigliptin, a novel dipeptidyl peptidase 4 inhibitor, in humans. Xenobiotica 2014, 44 (6), 522–530. 10.3109/00498254.2013.865856. 24304170

[ref9] Baar M.; Blechert S. Graphitic Carbon Nitride Polymer as a Recyclable Photoredox Catalyst for Fluoroalkylation of Arenes. Chem. - Eur. J. 2015, 21 (2), 526–530. 10.1002/chem.201405505. 25413695

[ref10] Yet L. Chemistry and biology of salicylihalamide A and related compounds. Chem. Rev. 2003, 103 (11), 4283–4306. 10.1021/cr030035s. 14611264

[ref11] Wang H.; Cheng Y. Z.; Yu S. Y. Visible-light-promoted and photocatalyst-free trifluoromethylation of enamides. Sci. China Chem. 2016, 59 (2), 195–198. 10.1007/s11426-015-5528-1.

[ref12] Courant T.; Dagousset G.; Masson G. enamide Derivatives: Versatile Building Blocks for Total Synthesis. Synthesis-Stuttgart 2015, 47 (13), 1799–1826. 10.1055/s-0034-1378706.

[ref13] Matsubara R.; Kobayashi S. Enamides and enecarbamates as nucleophiles in stereoselective C-C and C-N bond-forming reactions. Acc. Chem. Res. 2008, 41 (2), 292–301. 10.1021/ar700098d. 18281949

[ref14] Gopalaiah K.; Kagan H. B. Use of Nonfunctionalized Enamides and Enecarbamates in Asymmetric Synthesis. Chem. Rev. 2011, 111 (8), 4599–4657. 10.1021/cr100031f. 21568332

[ref15] Beltrana F.; Miesch L. Tertiary Enamides as Versatile and Valuable Substrates to Reach Chemical Diversity. Synthesis 2020, 52 (17), 2497–2511. 10.1055/s-0040-1707403.

[ref16] Gigant N.; Chausset-Boissarie L.; Gillaizeau I. Direct Metal-Catalyzed Regioselective Functionalization of Enamides. Chem. - Eur. J. 2014, 20 (25), 7548–7564. 10.1002/chem.201402070. 24862089

[ref17] Carbery D. R. Enamides: valuable organic substrates. Org. Biomol Chem. 2008, 6 (19), 3455–3460. 10.1039/b809319a. 19082143

[ref18] Feng C.; Loh T. P. Copper-catalyzed olefinic trifluoromethylation of enamides at room temperature. Chem. Sci. 2012, 3 (12), 3458–3462. 10.1039/c2sc21164e.

[ref19] Rey-Rodriguez R.; Retailleau P.; Bonnet P.; Gillaizeau I. Iron-Catalyzed Trifluoromethylation of enamide. Chem. - Eur. J. 2015, 21 (9), 3572–3575. 10.1002/chem.201406333. 25611040

[ref20] Carboni A.; Dagousset G.; Magnier E.; Masson G. Photoredox-Induced Three-Component Oxy-, Amino-, and Carbotrifluoromethylation of Enecarbamates. Org. Lett. 2014, 16 (4), 1240–1243. 10.1021/ol500374e. 24520865

[ref21] Tang K.; Chen Y. X.; Guan J. P.; Wang Z. J.; Chen K.; Xiang H. Y.; Yang H. Visible-light-promoted olefinic trifluoromethylation of enamides with CF3SO2Na. Org. Biomol Chem. 2021, 19 (34), 7475–7479. 10.1039/D1OB01410B. 34612366

[ref22] Jiang H.; Huang C.; Guo J.; Zeng C.; Zhang Y.; Yu S. Direct C-H Functionalization of Enamides and Enecarbamates by Using Visible-Light Photoredox Catalysis. Chem. - Eur. J. 2012, 18 (47), 15158–15166. 10.1002/chem.201201716. 23018775

[ref23] Winterson B.; Rennigholtz T.; Wirth T. Flow electrochemistry: a safe tool for fluorine chemistry. Chem. Sci. 2021, 12 (26), 9053–9059. 10.1039/D1SC02123K. 34276934 PMC8261735

[ref24] Kisukuri C. M.; Fernandes V. A.; Delgado J. A. C.; Häring A. P.; Paixao M. W.; Waldvogel S. R. Electrochemical Installation of CFH-, CF,H-, CF -, and Perfluoroalkyl Groups into Small Organic Molecules. Chem. Rec 2021, 21 (9), 2502–2525. 10.1002/tcr.202100065. 34151507

[ref25] Zou Z. L.; Zhang W. G.; Wang Y.; Kong L. Y.; Karotsis G.; Wang Y.; Pan Y. Electrochemically Promoted Fluoroalkylation-Distal Functionalization of unactivated Alkenes. Org. Lett. 2019, 21 (6), 1857–1862. 10.1021/acs.orglett.9b00444. 30817165

[ref26] Xu C. Z.; Liu Y.; Liu H.; Ma J. J.; He X.; Wu H. F.; Li Y. Q.; Sun Z. Z.; Chu W. Y. Metal-free electrochemical oxidative trifluoromethylation/C(sp)-H functionalization of quinolinones. Tetrahedron Lett. 2020, 61 (35), 152226 10.1016/j.tetlet.2020.152226.

[ref27] Leclercq E.; Moncomble A.; Debavelaere C.; Beaucamp M.; Penhoat M.; Chausset-Boissarie L. Electrolyte-free electrochemical C-H trifluoromethylation of 2-pyridones under batch and flow conditions. Green Chem. 2022, 24 (19), 7388–7394. 10.1039/D2GC02326A.

[ref28] Kang J. C.; Tu Y. Q.; Dong J. W.; Chen C.; Zhou J.; Ding T. M.; Zai J. T.; Chen Z. M.; Zhang S. Y. Electrochemical Semipinacol Rearrangements of Allylic Alcohols: Construction of All-Carbon Quaternary Stereocenters. Org. Lett. 2019, 21 (8), 2536–2540. 10.1021/acs.orglett.9b00263. 30945551

[ref29] Sun X.; Ma H. X.; Mei T. S.; Fang P.; Hu Y. L. Electrochemical Radical Formyloxylation-Bromination, -Chlorination, and -Trifluoromethylation of Alkenes. Org. Lett. 2019, 21 (9), 3167–3171. 10.1021/acs.orglett.9b00867. 30995058

[ref30] Huang Y. B.; Hong H. L.; Zou Z. R.; Liao C. S.; Lu J. J.; Qin Y. W.; Li Y. B.; Chen L. Electrochemical vicinal aminotrifluoromethylation of alkenes: high regioselective acquisition of β-trifluoromethylamines. Org. Biomol Chem. 2019, 17 (20), 5014–5020. 10.1039/C9OB00717B. 31042248

[ref31] Zhang L. L.; Zhang G. T.; Wang P.; Li Y. L.; Lei A. W. Electrochemical Oxidation with Lewis-Acid Catalysis Leads to Trifluoromethylative Difunctionalization of Alkenes Using CF3SO2Na. Org. Lett. 2018, 20 (23), 7396–7399. 10.1021/acs.orglett.8b03081. 30461286

[ref32] Jud W.; Kappe C. O.; Cantillo D. Catalyst-Free Oxytrifluoromethylation of Alkenes through Paired Electrolysis in Organic-Aqueous Media. Chem. - Eur. J. 2018, 24 (65), 17234–17238. 10.1002/chem.201804708. 30285302

[ref33] Leclercq E.; Barakat W.; Maazaoui R.; Penhoat M.; Gillaizeau I.; Chausset-Boissarie L. Electrochemical Trifluoromethylalkoxylation of Endocyclic Enamides in Batch and Flow. Adv. Synth Catal 2024, 366, 2919 10.1002/adsc.202301485.

[ref34] Zhang F. K.; Zhao X. F.; Zhang J.; Zhao L. L.; Li L.; Yang J. Y.; Li H.; Luo H. Q. Electrochemically Induced Regio- and Stereoselective ()-β-C(sp)-H Trifluoromethylation and Arylsulfonylation of Enamides. Adv. Synth Catal 2022, 364 (23), 4036–4042. 10.1002/adsc.202200934.

[ref35] Nikl J.; Lips S.; Schollmeyer D.; Franke R.; Waldvogel S. R. Direct Metal- and Reagent-Free Sulfonylation of Phenols with Sodium Sulfinates by Electrosynthesis. Chem. - Eur. J. 2019, 25 (28), 6891–6895. 10.1002/chem.201900850. 30861196

[ref36] Röckl J. L.; Schollmeyer D.; Franke R.; Waldvogel S. R. Dehydrogenative Anodic C-C Coupling of Phenols Bearing Electron-Withdrawing Groups. Angew. Chem. Int. Edit 2020, 59 (1), 315–319. 10.1002/anie.201910077. PMC6973026 31498544

[ref37] Han L. L.; Huang M. M.; Li Y. B.; Zhang J. Y.; Zhu Y.; Kim J. K.; Wu Y. J. An electrolyte- and catalyst-free electrooxidative sulfonylation of imidazo[1,2-]pyridines. Org. Chem. Front 2021, 8 (12), 3110–3117. 10.1039/D1QO00038A.

[ref38] He W. B.; Zhao S. J.; Chen J. Y.; Jiang J.; Chen X.; Xu X. H.; He W. M. External electrolyte-free electrochemical one-pot cascade synthesis of 4-thiocyanato-1 H-pyrazoles. Chin. Chem. Lett. 2023, 34 (2), 107640 10.1016/j.cclet.2022.06.063.

[ref39] Song H. Y.; Jiang J.; Song Y. H.; Zhou M. H.; Wu C.; Chen X.; He W. M. Supporting-electrolyte-free electrochemical [2 + 2+1] annulation of benzo[]isothiazole 1,1-dioxides,-arylglycines and paraformaldehyde. Chin. Chem. Lett. 2024, 35 (6), 109246 10.1016/j.cclet.2023.109246.

[ref40] Garrido-Castro A. F.; Hioki Y.; Kusumoto Y.; Hayashi K.; Griffin J.; Harper K. C.; Kawamata Y.; Baran P. S. Scalable Electrochemical Decarboxylative Olefination Driven by Alternating Polarity. Angew. Chem., Int. Ed. 2023, 62 (42), e202309157 10.1002/anie.202309157. 37656907

[ref41] Kawamata Y.; Hayashi K.; Carlson E.; Shaji S.; Waldmann D.; Simmons B. J.; Edwards J. T.; Zapf C. W.; Saito M.; Baran P. S. Chemoselective Electrosynthesis Using Rapid Alternating Polarity. J. Am. Chem. Soc. 2021, 143 (40), 16580–16588. 10.1021/jacs.1c06572. 34596395 PMC8711284

[ref42] Bhaskaran R. P.; Babu B. P. Progress in Electrochemical Trifluoromethylation Reactions. Adv. Synth Catal 2020, 362 (23), 5219–5237. 10.1002/adsc.202000996.

