# Supplementary material for: Electrochemical Trifluoromethylation of Enamides under Microflow Conditions
Source: Org Process Res Dev. 2024 Oct 22;28(11):4018–23. doi: 10.1021/acs.oprd.4c00311 (PMC11575483; doi:10.1021/acs.oprd.4c00311)
Supplement: Supplementary file 1 — op4c00311_si_001.docx [file op4c00311_si_001.docx]

**Electronic Supplementary Information**

**Electrochemical trifluoromethylation of enamides under microflow conditions**

Anna Vanluchene,^*^ Tomas Horsten, Eli Bonneure, Christian V. Stevens

^1^Department of Green Chemistry and Technology, Faculty of Bioscience Engineering, Ghent University, Coupure Links 653, 9000 Ghent, Belgium.

*Corresponding Author: Anna.Vanluchene@vscht.cz

Table of Contents

[1. Additional optimisation data 1](#_Toc172815503)

[2. Effect of supporting electrolyte on the product yield in batch 2](#_Toc172815504)

[3. Fouling of electrodes 3](#_Toc172815505)

[4. Cyclic voltammetry 3](#_Toc172815506)

[5. Set-up of microflow cell with flushing step 7](#_Toc172815507)

[6. Characterisation data of compounds 3a – 3n 8](#_Toc172815508)

[7. Compounds spectra 12](#_Toc172815509)

# Additional optimisation data

Herein we provide additional optimisation data. Different production rate was tested for initial conditions (reaction mixture ACN with 3 equivalents of CF_3_SO_2_Na, and graphite electrodes). Optimal production rate was corresponding with constant current of 10 mA (*j* = 1.5 mA/cm^2^) and a flow rate of 155 μl/min, higher production rate (constant current of 20 mA (*j* = 2.9 mA/cm^2^) and a flow rate of 311 μl/min) led to drop in yield. From brief estimation of Reynolds number was also obvious that for all production rate laminar flow is present in the microchannel (Re << 2400).

Table S1: Comparison of different production rate

| I  [mA] | **j  [mA/cm^2^]** | q  [F] | **Q  [ul/min]** | **Y [%]** | Re  [-] |
| --- | --- | --- | --- | --- | --- |
| 5 | 0.7 | 2 | 78 | 62 | 23 |
| 10 | 1.5 | 2 | 155 | 65 | 46 |
| 20 | 2.9 | 2 | 311 | 52 | 96 |

Different channel design did not have significant effect on the final yield. All PFA channel films were purchased from Analytical Sales, Inc. (Simple channel film: F1088706; Tangential mixer channel film: F1088707; Meandering channel film: F1088703; Fin separator channel film: F1088710).

Table S2: Comparison of different channel designs

| **channel  type** | **Y [%]** |
| --- | --- |
| simple | 74 |
| tangential | 69 |
| meandering | 62 |
| fin separator | 66 |

# Effect of supporting electrolyte on the product yield in batch

The microflow cell and batch process were compared under the same optimised reaction conditions (0.2 mmol of substrate, 2 equiv. CF_3_SO_2_Na, 10 mL ACN, 10 mA, C (+)/Ag(-), 2 F). To reduce the overpotential in the batch process, several supporting electrolytes were tested, including LiClO_4_, Bu_4_NClO_4_, TBAPF_6_, Et_4_NI, and Et_4_NBF_4_ (0.1 M). However, the yields were lower for all tested salts compared to the reaction run without any supporting electrolyte and in the microflow reactor.

Table S3: Effect of supporting electrolyte on the product yield in batch

| **Entry** | **electrolyte** | **Y [%]** |
| --- | --- | --- |
| 1 | no | 51 |
| 2 | LiClO_4_ | 6 |
| 3 | Bu_4_NClO_4_ | 48 |
| 4 | TBAPF_6_ | 17 |
| 5 | Et_4_NI | 0 |
| 6 | Et_4_NBF_4_ | 39 |
| 7 | Bu_4_NBF_4_ | 35 |

# Fouling of electrodes

Table S4: Evaluation of electrode fouling by measuring anode
and cathode weights before and after the reaction


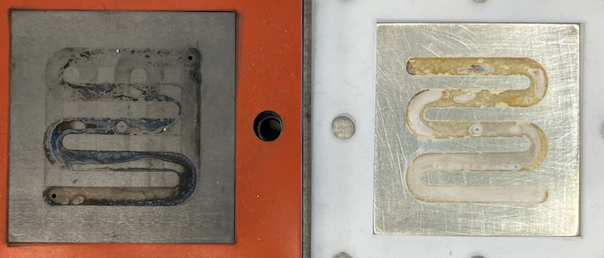

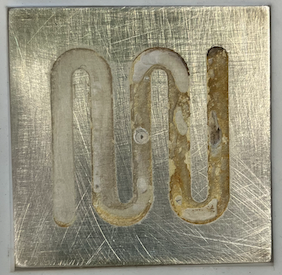


**Anode**

**Cathode**


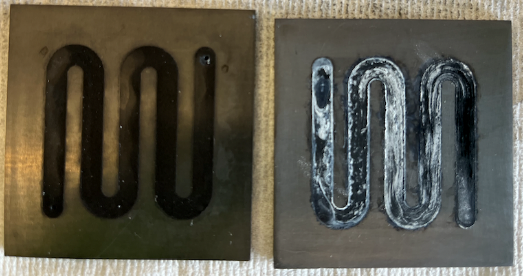


**Anode**

**Cathode**

**A)**

**B)**

Figure S1: Fouling of electrodes in the microflow cell: A) C(+)/C(-); B) C(+)/Ag(-)

| **Entry** | **Reaction description** | **anode [mg]** | **cathode [mg]** | **total [mg]** |
| --- | --- | --- | --- | --- |
| 4 | C(+)/C(-), CC, 10 mA | 5.2 | 16.2 | 21.4 |
| 15 | C(+)/C(-), AP (5s), 10 mA | 2.1 | 3.3 | 5.4 |
| 16 | C(+)/C(-), AP (20s), 10 mA | 2.4 | 3.5 | 5.9 |
| 17 | C(+)/C(-), AP (100s), 10 mA | 3.0 | 5.1 | 8.1 |
| 18 | C(+)/C(-), AP (100s), 20 mA | 6.4 | 7.5 | 13.9 |
| 19 | C(+)/C(-), rAP (0.1s), 20 mA | 0.7 | 1.1 | 1.8 |
| 21 | C(+)/Ag(-), CC, 10 mA | 0.5 | 8.4 | 8.9 |

# Cyclic voltammetry

The cyclic voltammetry curves were recorded at room temperature using Et_4_NBF_4_ (250 mM) and 10mM of the investigated compound (CF_3_SO_2_Na or substrate corresponding to product 3a – 3n) in ACN. A glassy carbon disc working electrode (d = 3 mm), a Pt wire counter electrode and an Ag/AgCl reference electrode were used. The scan rate is 100 mV/s. Recorded by Biologic SP-50 Potentiostat.

**Cyclic voltammetry of substrates corresponding to products 3a – 3n and CF_3_SO_2_Na**

Table S5: Overview of oxidational potential for all substrates

| **Compound** | **Ep_1/2  [V vs Ag/AgCl]** |
| --- | --- |
| CF_3_SO_2_Na | 1.17 |
| 3a | 1.21 |
| 3b | 1.28 |
| 3c | 1.30 |
| 3d | 1.23 |
| 3e | 1.22 |
| 3f | 1.72 |
| 3g | 1.30 |
| 3h | 1.11 |
| 3i | 1.15 |
| 3j | 1.61 |
| 3k | 1.56 |
| 3l | 1.47 |
| 3m | 1.50 |
| 3n | 1.96 |

# Set-up of microflow cell with flushing step


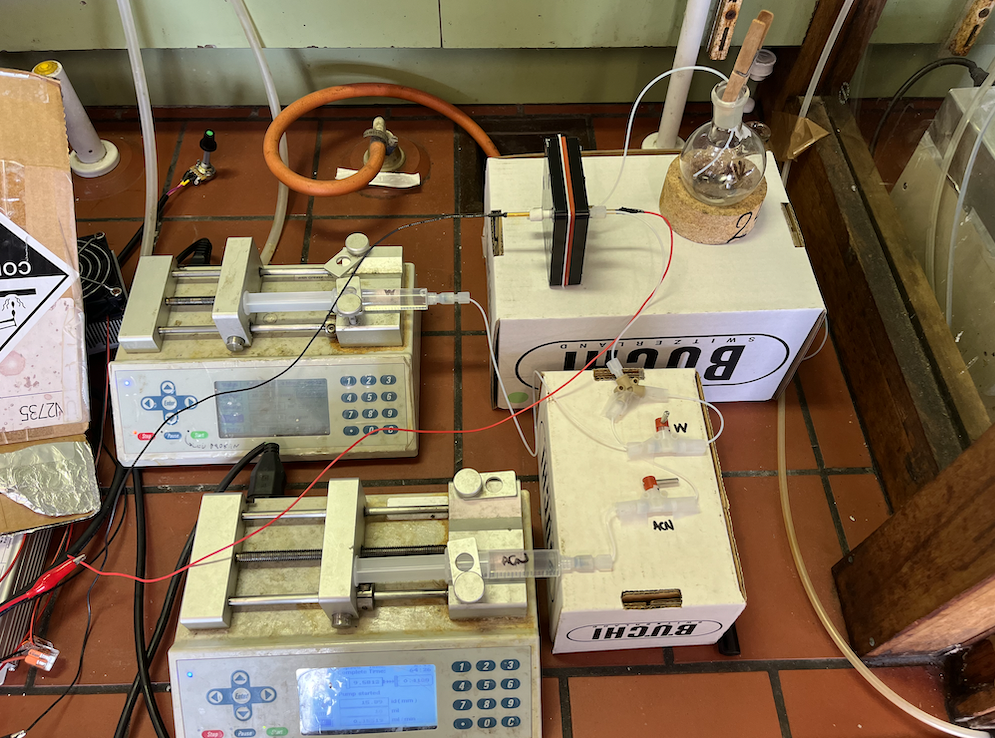


reaction mixture

water

valves

Y-junction

microflow cell

inlet

outlet

connection to power supply

Figure S2: Set-up of microflow cell with flushing step.

**
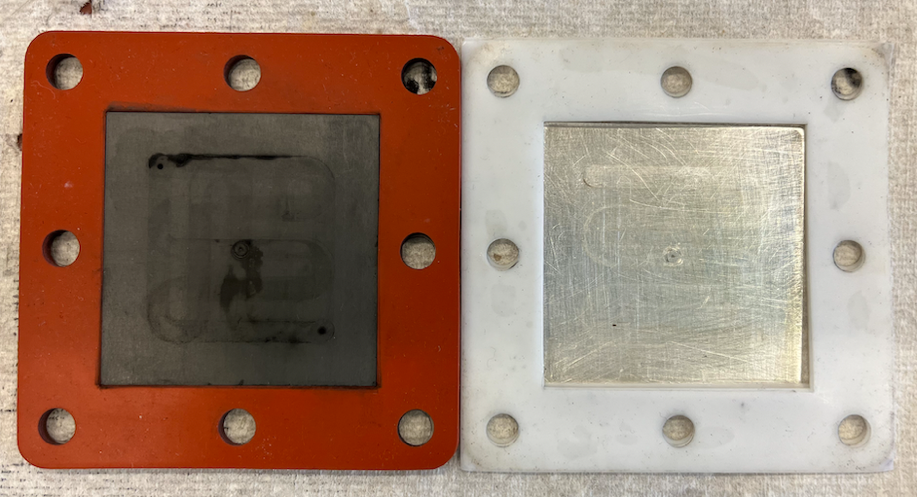
**

Figure S3: Electrodes C(+)/Ag(-) after the electrolysis with flushing step incorporated

# Characterisation data of compounds 3a – 3n

The chemical shifts are reported in parts per million (ppm), coupling constants (*J*) are quoted in Hz. Multiplicities are reported as s (singlet), d (doublet), t (triplet), q (quartet), m (multiplet), br (broad), dd (doublet of doublet) and dq (doublet of quartet).

**(3a) *tert*-butyl 5-(trifluoromethyl)-3,4-dihydropyridine-1(2*H*)-carboxylate**

Purified by flash silica gel column chromatography: Hex/EtOAc = 95/5; yield 61 %; colourless oil. Spectral data is in accordance with literature.^1^

**^1^H-NMR** (400 MHz, CDCl_3_) (rotamers): δ 7.54 & 7.34 (1H), 3.61-3.54 (br,2H), 2.18 (t, *J* = 6.2 Hz, 2H), 1.88 (q, *J* = 6.1, 2H), 1.51 (s, 9H)

**^13^C-NMR** (100,6 MHz, CDCl_3_) (rotamers) : δ 151.8 & 152.4, 128.1 (q, *J* = 7.8 Hz), 124.8 (q,  *J* = 268.7 Hz), 105.6 (q, *J* = 27.6 Hz), 82.1, 42.2, 41.1, 28.1, 20.4, 19.3; **^19^F-NMR** (376,5 MHz, CDCl_3_): δ -66.54, -66.62.

**(3b) benzyl 5-(trifluoromethyl)-3,4-dihydropyridine-1(2*H*)-carboxylate**

Purified by flash silica gel column chromatography: Hex/EtOAc = 95/5; yield 65 %; colourless oil. Spectral data is in accordance with literature.^2^

**^1^H-NMR** (400 MHz, CDCl_3_) (rotamers) : δ 7.56 & 7.42 (s, 1H), 7.40 - 7.31 (m, 5H), 5.22 (s, 2H), 3.63 (t, *J* = 5.0 Hz, 2H), 2.19 (t, *J* = 5.4 Hz, 2H),1.95 - 1.84 (br, 2H)

**^13^C-NMR** (100,6 MHz, CDCl_3_) (rotamers): δ 153.5 & 152.9, 135.6, 128.7, 128.5, 128.3, 127.7 - 127.2 (m), 124.6 (q, *J* = 270.2 Hz), 107.1 (q, *J* = 26.3 Hz), 68.3, 42.1&41.9, 20.3, 19.4&19.1

**^19^F-NMR** (376,5 MHz, CDCl_3_): δ - 66.7, - 66.8.

**(3c) methyl 5-(trifluoromethyl)-3,4-dihydropyridine-1(2*H*)-carboxylate**

^^Purified by flash silica gel column chromatography: Hex/EtOAc = 95/5; yield 44 %; colourless oil.

**^1^H-NMR** (400 MHz, CDCl_3_) (rotamers): δ 7.53 & 7.39 (1H), 3.81 (3H), 3.66 - 3.57 (br, 2H), 2.19 (t, *J* = 6.0 Hz, 2H), 1.90 (q, *J* = 6.0 Hz, 2H)

**^13^C-NMR** (100,6 MHz, CDCl_3_): δ 154.1 & 153.4, 128.0, 127.6, 124.6 (q, *J* = 269.0 Hz), 106.7, 53.5, 42.0, 41.8, 20.3, 19.3 &19.1

**^19^F-NMR** (376,5 MHz, CDCl_3_): δ -66.77, -66.81.

**HRMS** (ESI) ([M+H]^+^) Calcd for C_8_H_11_F_3_NO_2_: 210.07419; Found: 210.07339

**(3d) *tert*-butyl 2-methyl-5-(trifluoromethyl)-3,4-dihydropyridine-1(2*H*)-carboxylate**

Purified by flash silica gel column chromatography: Hex/EtOAc = 95/5; yield 61 %; colourless oil.

**^1^H-NMR** (400 MHz, CDCl_3_): δ 7.40 & 7.24 (1H), 4.40-4.17 (br,1H), 2.19-2.05 (br, 2H), 1.77-1.62 (br, 2H), 1.44 (s, 9H), 1.04 (d, *J* = 6.7 Hz, 3H)

**^13^C-NMR** (100,6 MHz, CDCl_3_): δ 152.1 & 151.4, 126.8 (q, *J* = 6.85 Hz), 124.9 (q, *J* = 268.71 Hz), 81.9, 46.5, 45.5, 28.1, 25.4, 17.2, 15.2

**^19^F-NMR** (376,5 MHz, CDCl_3_): δ -66.31.

**HRMS** (ESI) ([M-C_4_H_8_+H]^+^) Calcd for C_8_H_11_F_3_NO_2_: 210.07419; Found: 210.07344

**(3e) *tert*-butyl 4-methyl-5-(trifluoromethyl)-3,4-dihydropyridine-1(2*H*)-carboxylate**

****Purified by flash silica gel column chromatography: Hex/EtOAc = 95/5; yield 47 %; colourless oil.

**^1^H-NMR** (400 MHz, CDCl_3_): δ 7.46 & 7.27 (1H), 3.85-3.67 (m, 1H), 3.39-3.17 (m, 1H), 2.51-2.41 (m,1H), 1.80-1.67 (m, 1H), 1.65-1.54 (m, 1H), 1.44 (s, 9H), 1.06 (d, *J* = 6.9 Hz, 3H)

**^13^C-NMR** (100,6 MHz, CDCl_3_): δ 152.3, 151.7, 128.0, 125.2 (q, *J* = 269.9 Hz), 109.8 (q, *J* = 24.8 Hz), 82.1, 38.5, 37.6, 28.3, 28.1, 24.5, 20.2

**^19^F-NMR** (376,5 MHz, CDCl_3_): δ -63.21 & - 63.09

**HRMS** (ESI) ([M-C_4_H_8_+H]^+^) Calcd for C_8_H_11_F_3_NO_2_: 210.07419; Found: 210.07346

**(3f) *tert*-butyl 2-oxo-5-(trifluoromethyl)-3,4-dihydropyridine-1(2*H*)-carboxylate**

****Purified by flash silica gel column chromatography: Hex/EtOAc = 95/5 – 85/15; yield 12 %; colourless oil.

**^1^H-NMR** (400 MHz, CDCl_3_): δ 7.39 - 7.36 (m, 1H), 2.65 (t, *J* = 7.7Hz), 2.45 (t, *J* = 7.5 Hz), 1.49 (s, 9H)

**^13^C-NMR** (100,6 MHz, CDCl_3_): δ 167.2, 128.9 (q, *J* = 7.1 Hz), 149.30, 123.64 (q, *J* = 269.4 Hz), 109.44 (q, *J* = 33.1 Hz), 85.3, 32.6, 27.9, 18.6

**^19^F-NMR** (376,5 MHz, CDCl_3_): δ -67.20.

**HRMS** (ESI) ([M+Na]^+^) Calcd for C_11_H_14_F_3_NNaO_3_: 288.08235

Found: 288.08121.

**(3g) *tert*-butyl 6-(trifluoromethyl)-2,3,4,5-tetrahydro-1*H*-azepine-1-carboxylate**

Purified by flash silica gel column chromatography: Hex/EtOAc = 95/5; yield 35 %; colourless oil.

**^1^H-NMR** (400 MHz, CDCl_3_): δ 7.23 (s, 1H), 3.74 (t, *J* = 5.9 Hz, 2H), 2.37 -2.31 (m, 2H), 1.87 - 1.81 (m, 4H), 1.45 (s, 9H)

**^13^C-NMR** (100,6 MHz, CDCl_3_): δ 153.1, 133.6 (q, *J* = 6.87 Hz), 113.4, 113.1, 81.8, 47.2 - 45.61(br), 33.0, 31.6, 28.1, 27.1, 24.3, 23.5, 22.6, 14.1

**^19^F-NMR** (376,5 MHz, CDCl_3_): δ -66.77

**HRMS** (ESI) ([M-C_4_H_8_+H]^+^) Calcd for C_8_H_11_F_3_NO_2_: 210.07419

Found: 210.07341

**(3h) *tert*-butyl 4-(trifluoromethyl)-2,3-dihydro-1*H*-pyrrole-1-carboxylate**

****Purified by flash silica gel column chromatography: Hex/EtOAc = 95/5; yield 8 %; colourless oil.

**^1^H-NMR** (400 MHz, CDCl_3_) (rotamers): δ 7.10 & 6.92 (1H), 3.89 (t, *J =* 8.49 Hz, 2H), 2.87 - 2.74 (br, 2H), 1.50 (9H)

**^13^C-NMR** (100,6 MHz, CDCl_3_) (rotamers): δ 151.8 & 151.1, 133.5, 123.4 (q, *J* = 264.83 Hz), 109.7, 109.38, 81.7, 60.4, 46.4, 28.2, 27.3, 26.2

**^19^F-NMR** (376,5 MHz, CDCl_3_): δ - 63.47, -63.54.

**HRMS** (ESI) ([M-C_4_H_8_+H]^+^) Calcd for C_6_H_7_F_3_NO_2_: 182.04289

Found: 182.04210

**(3j) (*Z*)-1-(3,3,3-trifluoroprop-1-en-1-yl)pyrrolidin-2-one**

Purified by flash silica gel column chromatography: Hex/EtOAc = 90/10 – 60/40; yield 55 %; colourless oil. Spectral data is in accordance with literature.^3^

**^1^H-NMR** (400 MHz, CDCl_3_): δ 7.63 (dd, *J* = 14.4, 1.7 Hz, 1H), 5.01 (dq, *J* = 13.5, 6.6 Hz, 1H), 3.54 (t, *J* = 7.2 Hz, 2H), 2.55 (t, *J* = 8.2 Hz, 2H), 2.19 (q, *J* = 7.7 Hz, 2H)

**^13^C-NMR** (100,6 MHz, CDCl_3_): δ 174.2, 131.2 (q, *J* = 7.3 Hz), 124.4 (q, *J* = 267.5 Hz), 98.3 (q, *J* = 34.9 Hz), 44.8, 30.9, 17.4

**^19^F-NMR** (376,5 MHz, CDCl_3_): δ -60.36 & -60.37.

**(3k) (*Z*)-3-methyl-1-(3,3,3-trifluoroprop-1-en-1-yl)pyrrolidin-2-one**

****Purified by flash silica gel column chromatography: Hex/EtOAc = 80/20; yield 55 %; colourless oil.

**^1^H-NMR** (400 MHz, CDCl_3_): δ 7.54 (br d *J* = 14.4, 1.7 Hz, 1H) *,* 4.94 (dq, *J* = 13.5, 6.6 Hz, 1H), 3.47-3.39 (m, 1H), 3.39-3.30 (m,1H), 2.60-2.48 (m, 1H), 2.39-2.28 (m, 1H), 1.75-1.64 (m, 1H), 1.19 (d, *J* = 7.12 Hz)

**^13^C-NMR** (100,6 MHz, CDCl_3_): δ 176.6, 131.3 (q, *J* = 7.36 Hz), 124.4 (q, *J* = 267.3 Hz), 98.6 (q, *J* = 34.9 Hz), 43.0, 36.9, 26.5, 15.7

**^19^F-NMR** (376,5 MHz, CDCl_3_): δ -60.30, 60.31.

**HRMS** (ESI) ([M+H]^+^) Calcd for C_8_H_11_F_3_NO: 194.07927;

Found: 194.07854.

**(3l) (*Z*)-1-(3,3,3-trifluoroprop-1-en-1-yl)azepan-2-one**

Purified by flash silica gel column chromatography: Hex/EtOAc = 60/40; yield 4%; colourless oil. Spectral data is in accordance with literature.^3^

**^1^H-NMR** (400 MHz, CDCl_3_): δ 7.95 (dq, *J_1_* = 1.89 Hz, *J_2_* = 14.73 Hz, 1H), 5.08 (dq, *J_1_* = 6.45 Hz, *J_2_* = 13.72 Hz, 1H), 3.60 - 3.55 (m, 2H), 2.71- 2.65 (m, 2H), 1.82 - 1.67 (m, 6H)

**^13^C-NMR** (100,6 MHz, CDCl_3_): δ 174.8, 134.2 (q, *J* = 7.31 Hz), 124.9 (q, *J* = 267.48 Hz), 96.8 (q, *J* = 34.45 Hz), 45.4, 37.0, 29.2, 27.1, 23.3; **^19^F-NMR** (376,5 MHz, CDCl_3_): δ -59.65 & -59.66.

**(3m) (Z)-N-methyl-N-(3,3,3-trifluoroprop-1-en-1-yl)acetamide**

Purified by flash silica gel column chromatography: Hex/EtOAc = 80/20; yield 30%; colourless oil.

**^1^H-NMR** (400 MHz, CDCl_3_): δ 8.03 & 7.35 (2 x d, *J_1_* = 13.64 Hz, *J_2_* = 13.48 Hz, 1H), 5.10 - 4.97 (br, 1H), 3.10&3.15 (2 x br.s, 3H), 2.31 (s, 3H)

**^13^C-NMR** (100,6 MHz, CDCl_3_): δ 171.0, 169.6, 136.6-136.1 (br), 134.7-134.2 (br), 125.5, 122. 9, 98.1–96.3 (br), 32.8, 29.1, 22.6, 21.5;

**^19^F-NMR** (376,5 MHz, CDCl_3_): δ -59.95, - 60.00.

**HRMS** (ESI) ([M+H]^+^) Calcd for C_6_H_9_F_3_NO: 168.06307;
Found 168.0630

**(3n) methyl (*E*)-2-((*tert*-butoxycarbonyl)(methyl)amino)-4,4,4-trifluorobut-2-enoate**

Purified by flash silica gel column chromatography: Hex/EtOAc = 90/10; yield 67%; colourless oil.

**^1^H-NMR** (400 MHz, CDCl_3_): δ 5.28 (q, *J* = 7.8 Hz, 1H), 3.85 (s, 3H), 3.16 (s, 3H), 1.47 (s, 9H)

**^13^C-NMR** (100,6 MHz, CDCl_3_): δ 163.3, 152.2, 143.6 (q, J = 5.4 Hz), 122.9 (q, *J* = 268.8 Hz), 106.2-104.2 (br), 83.6, 52.7, 35.5, 27.9

**^19^F-NMR** (376,5 MHz, CDCl_3_): δ -56.39 & -56.41.

**HRMS** (ESI) ([M-C_4_H_8_+H]^+^) Calcd for C_7_H_9_F_3_NO_4_: 228.04837;

Found: 228.04747

**References:**1) Wang, H., Cheng, Y., & Yu, S. (2016). Visible-light-promoted and photocatalyst-free trifluoromethylation of enamides. *Science China Chemistry*, *59*, 195-198.

2) Cheng, Y., Yuan, X., Ma, J., & Yu, S. (2015). Direct Aromatic C-H Trifluoromethylation via an Electron‐Donor–Acceptor Complex. *Chemistry–A European Journal*, *21*(23), 8355-8359.

3) Rey‐Rodriguez, R., Retailleau, P., Bonnet, P., & Gillaizeau, I. (2015). Iron‐Catalyzed Trifluoromethylation of Enamide. *Chemistry–A European Journal*, *21*(9), 3572-3575.

# Compounds spectra


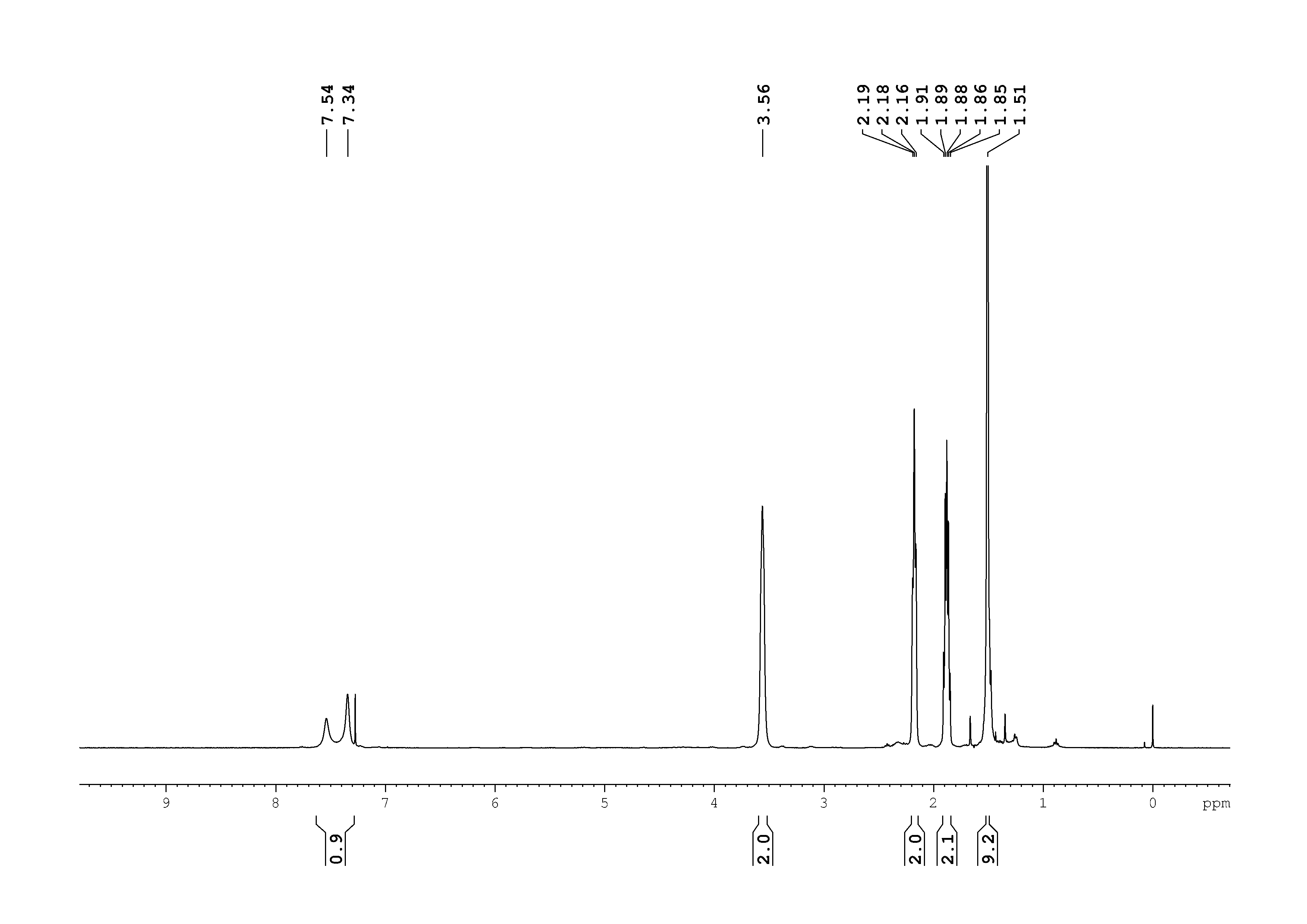
^1^H-NMR spectra of compound **3a**, CDCl_3_, 400.1 MHz


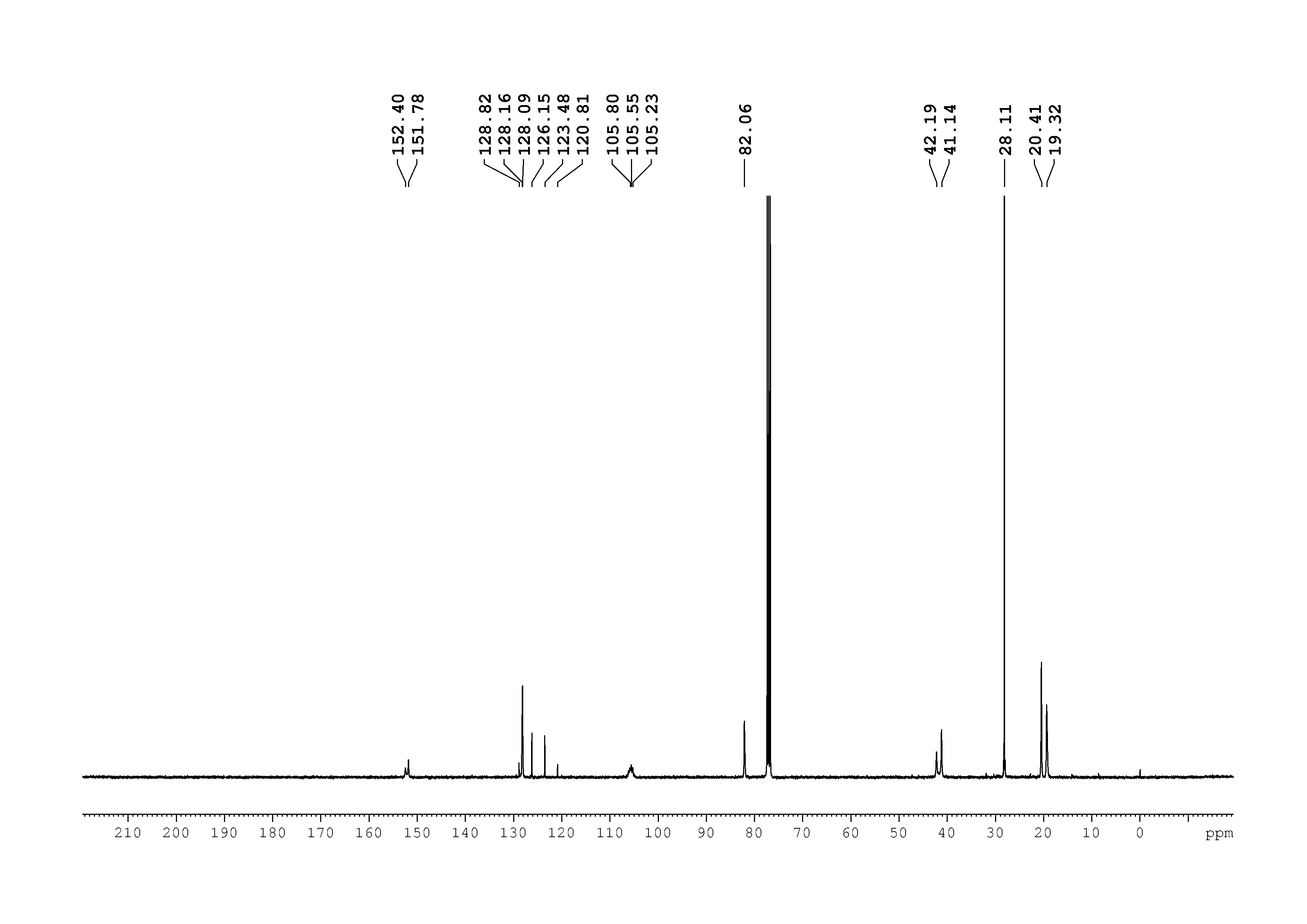
^1^C-NMR spectra of compound **3a**, CDCl_3_, 100.6 MHz


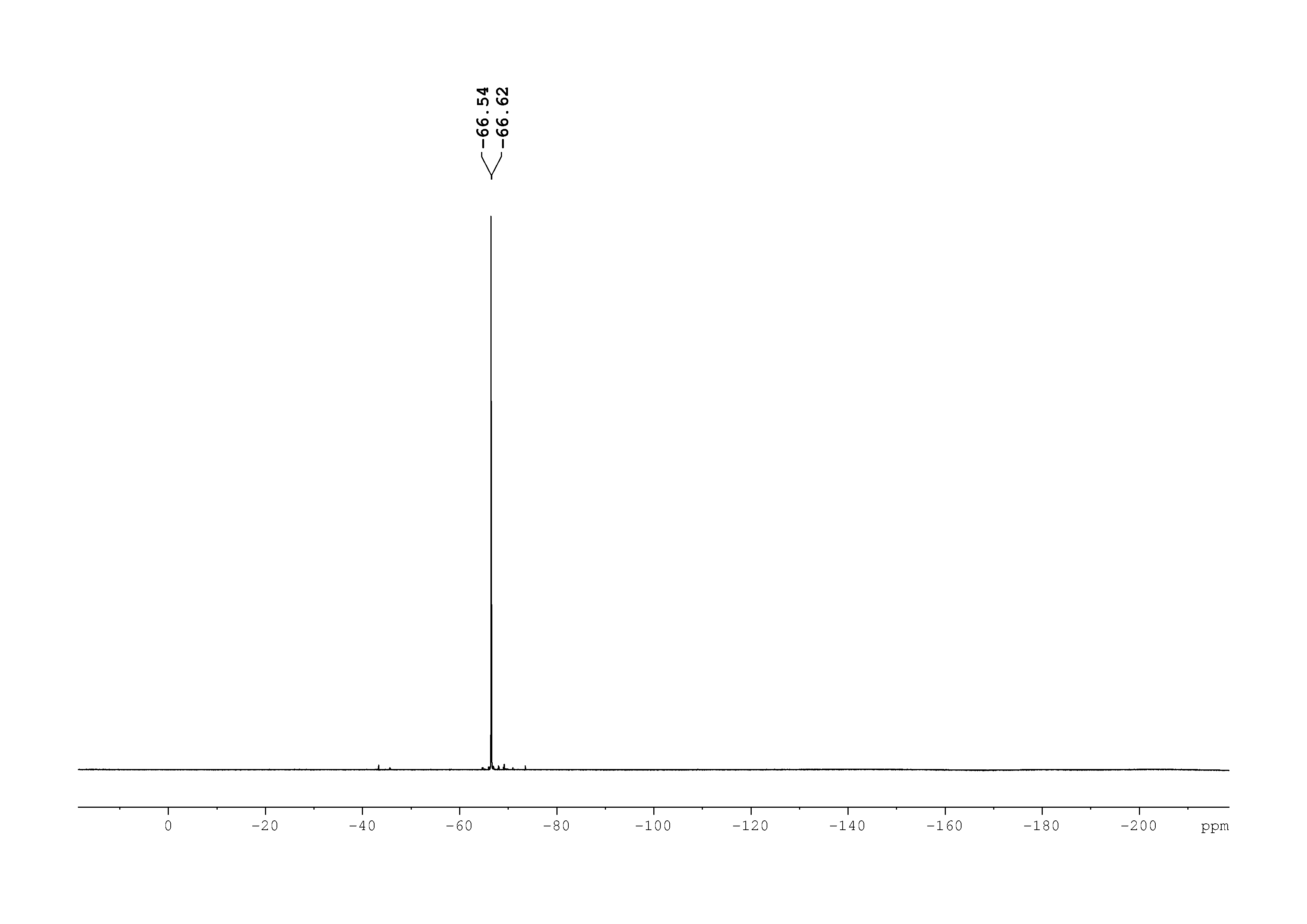
^19^F-NMR spectra of compound **3a**, CDCl_3_, 376.5 MHz


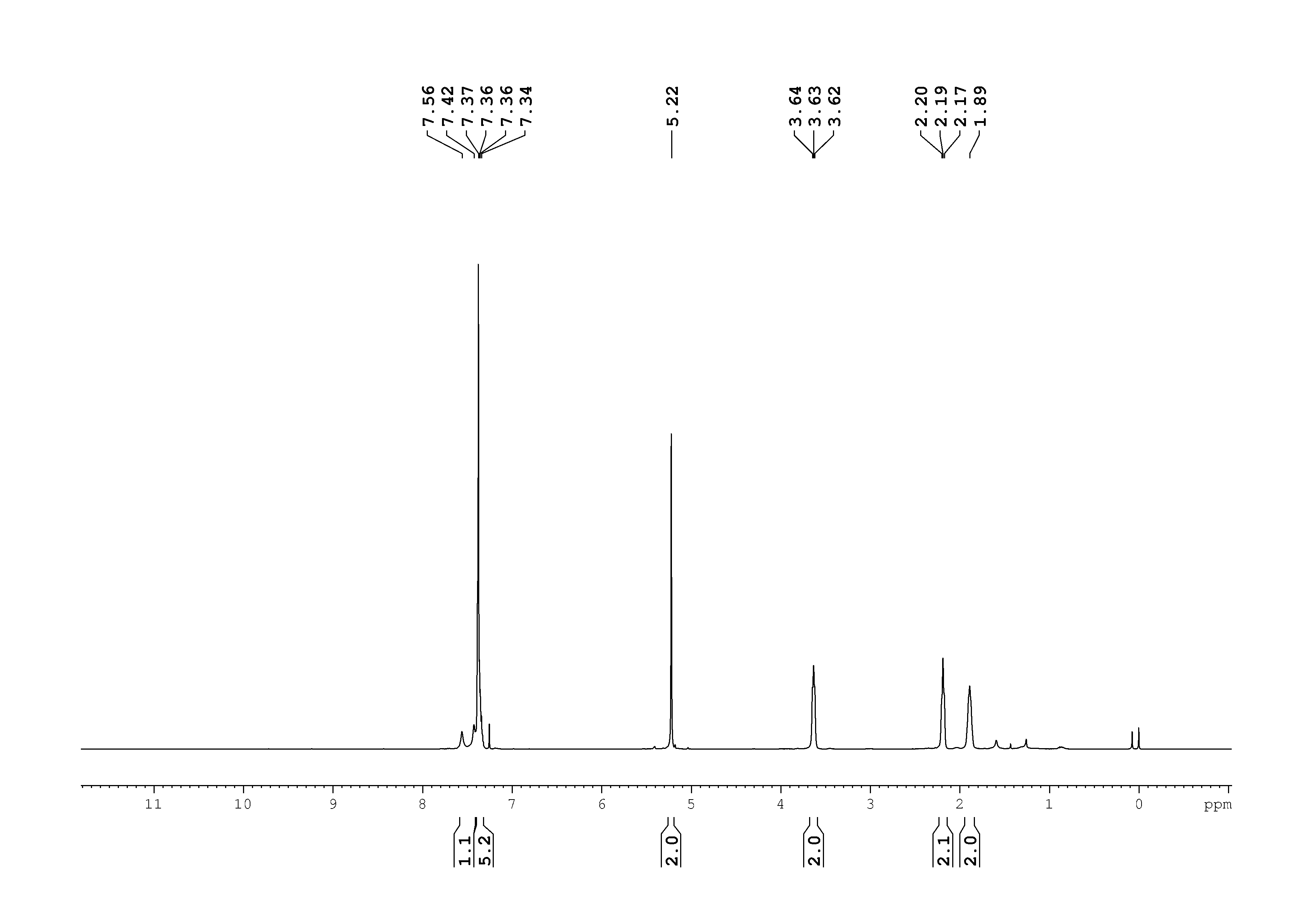
^1^H-NMR spectra of compound **3b**, CDCl_3_, 400.1 MHz


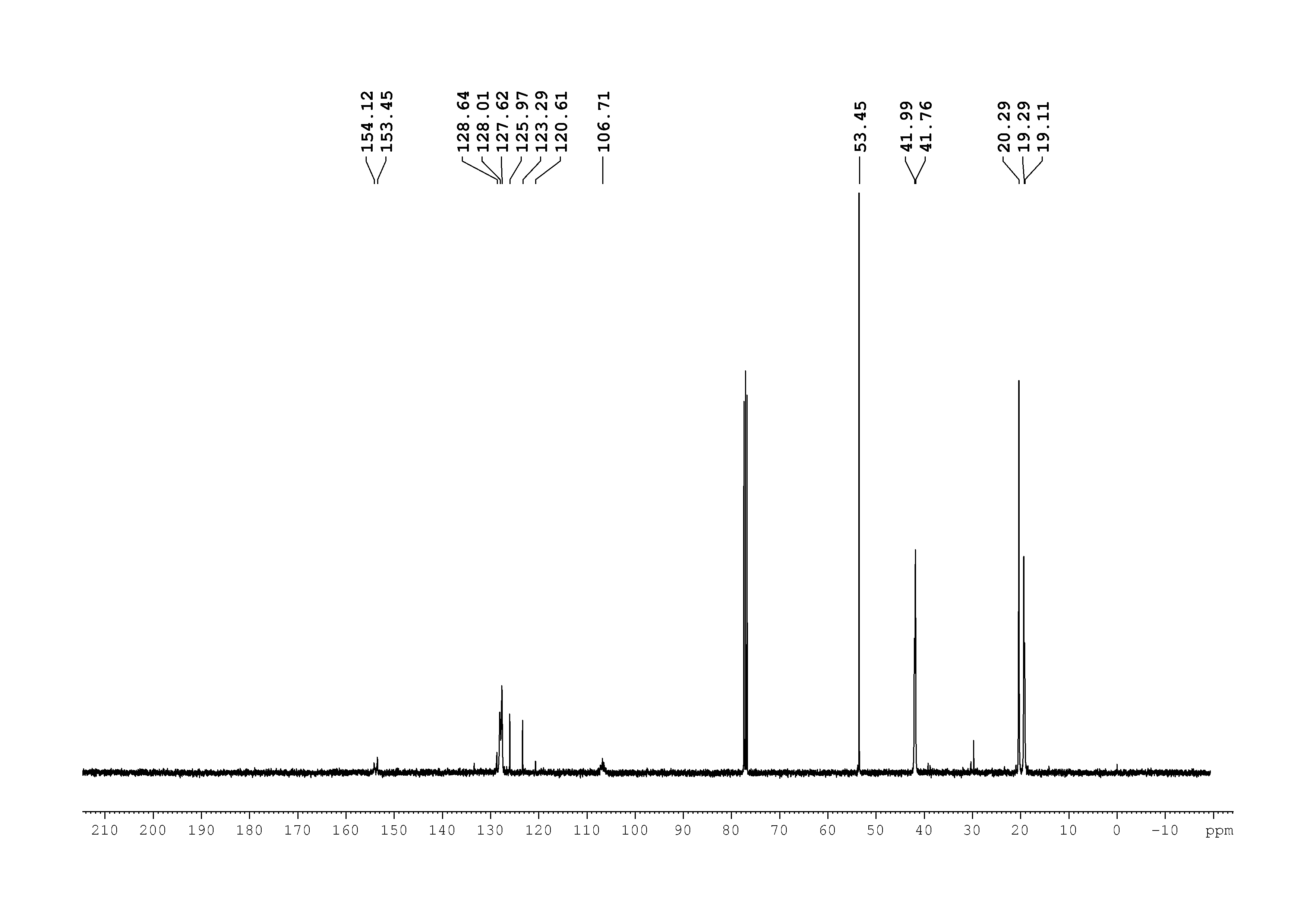
^13^C-NMR spectra of compound **3b**, CDCl_3_, 100.6 MHz


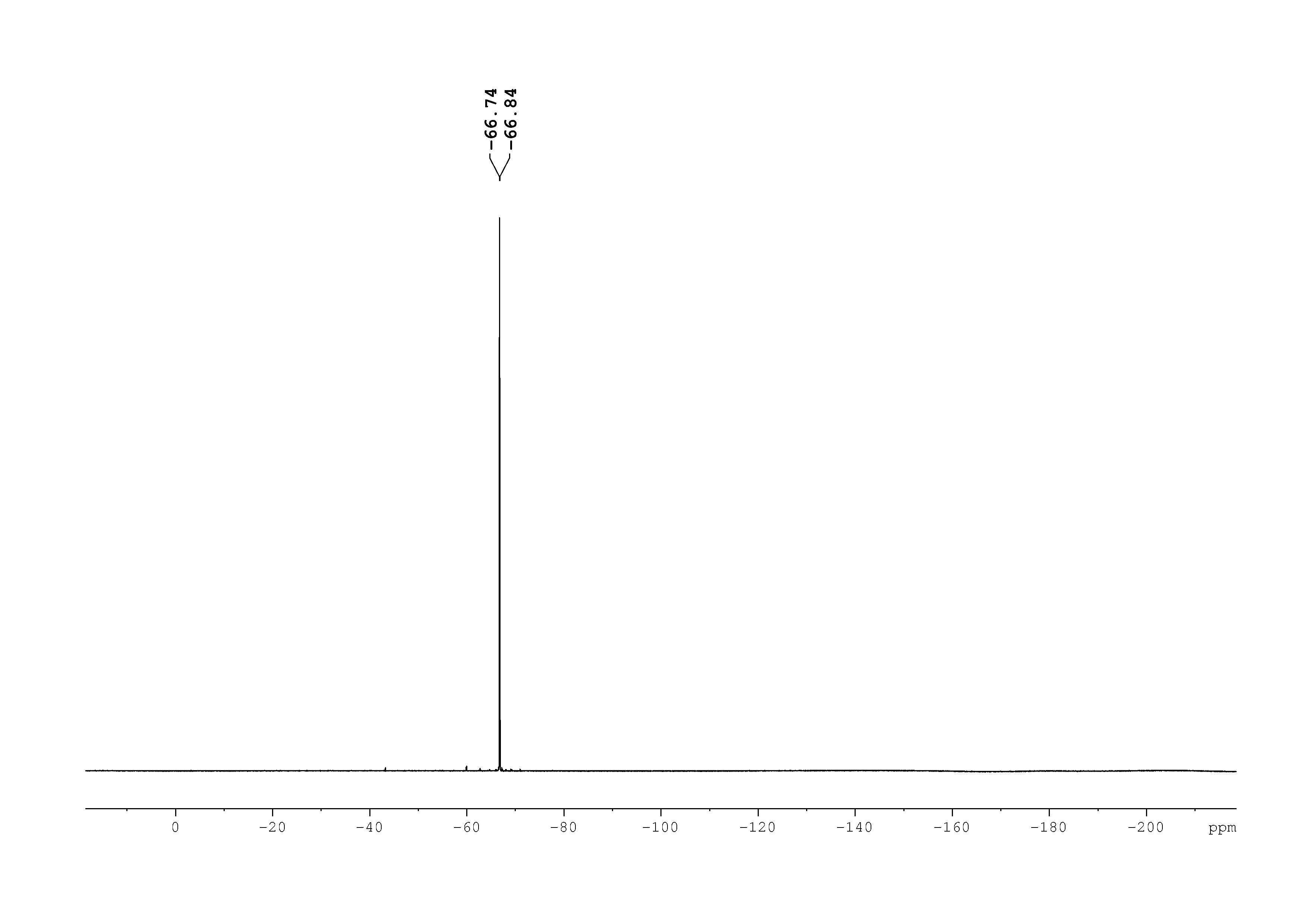
 ^19^F-NMR spectra of compound **3b**, CDCl_3_, 376.5 MHz


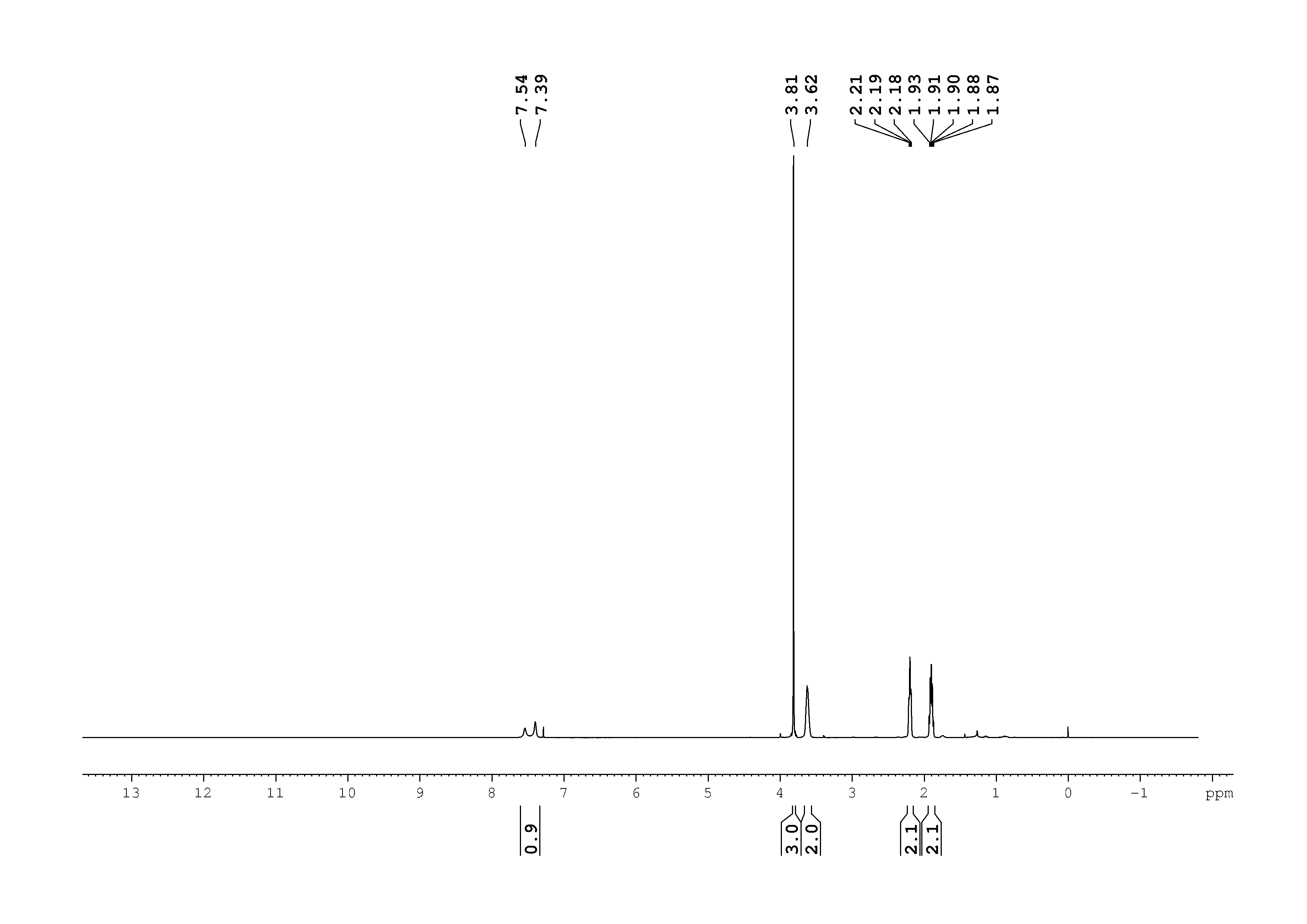
^1^H-NMR spectra of compound **3c**, CDCl_3_, 400.1 MHz


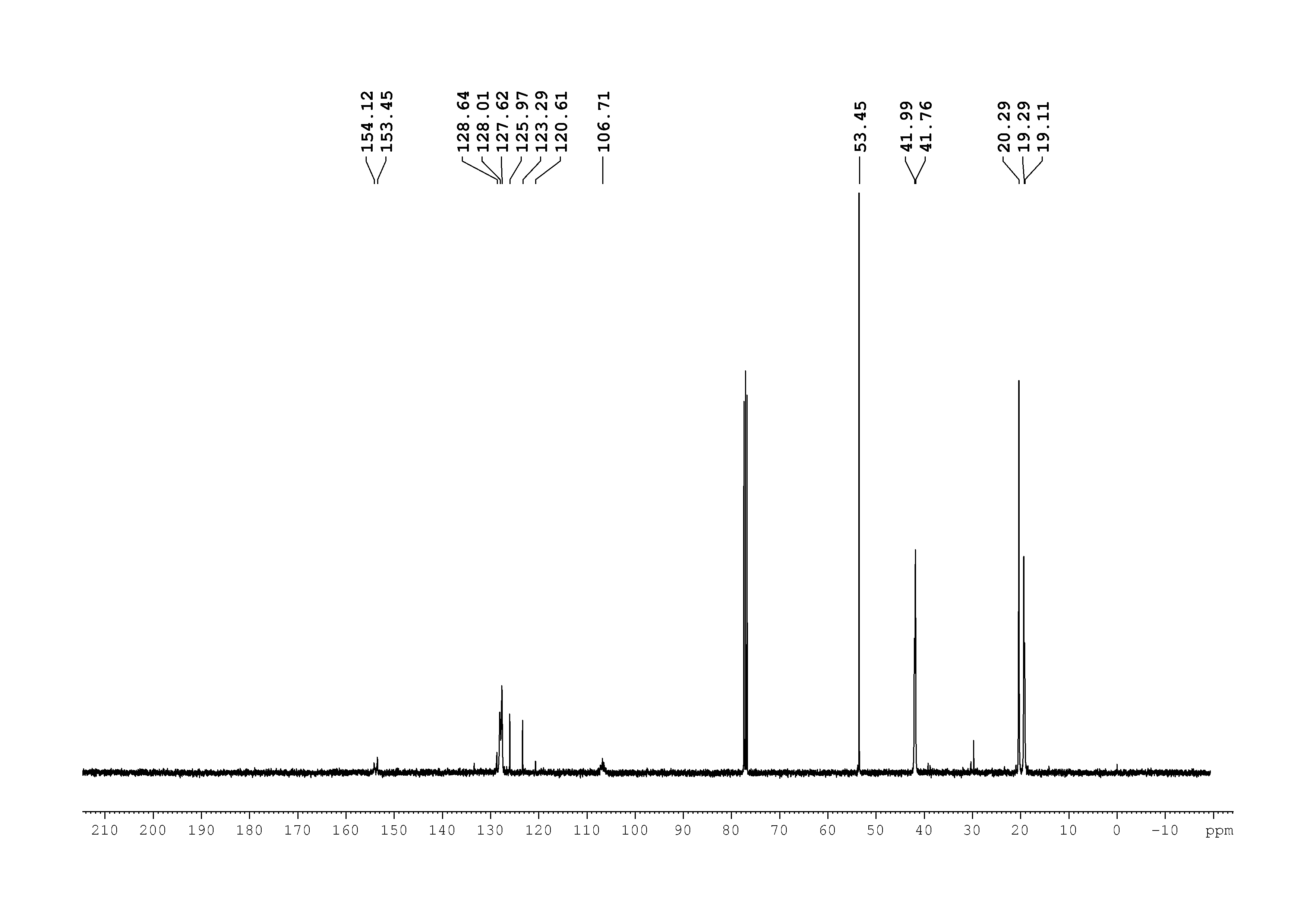
^13^C-NMR spectra of compound **3c**, CDCl_3_, 100.6 MHz


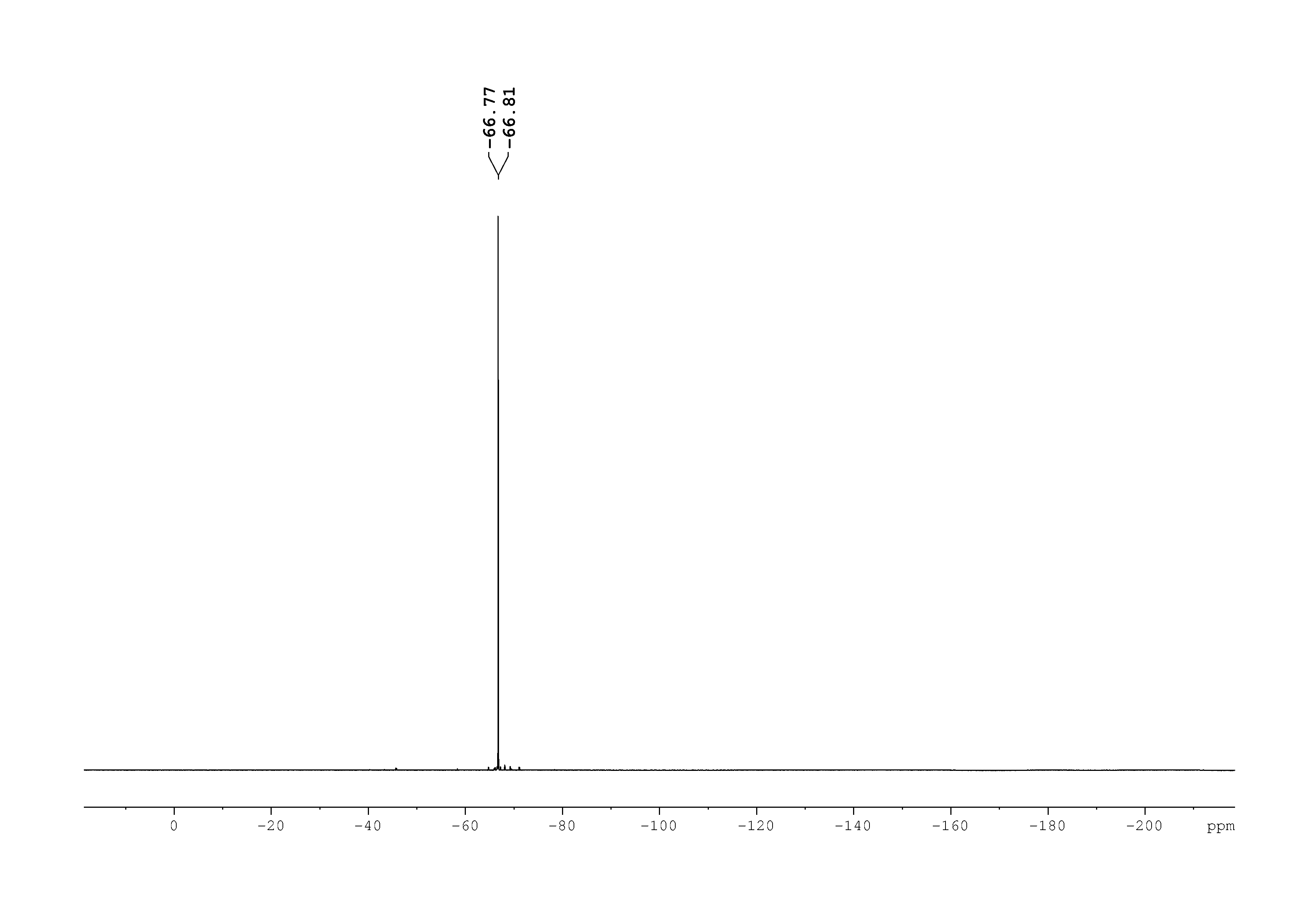
 ^19^F-NMR spectra of compound **3c**, CDCl_3_, 376.5 MHz


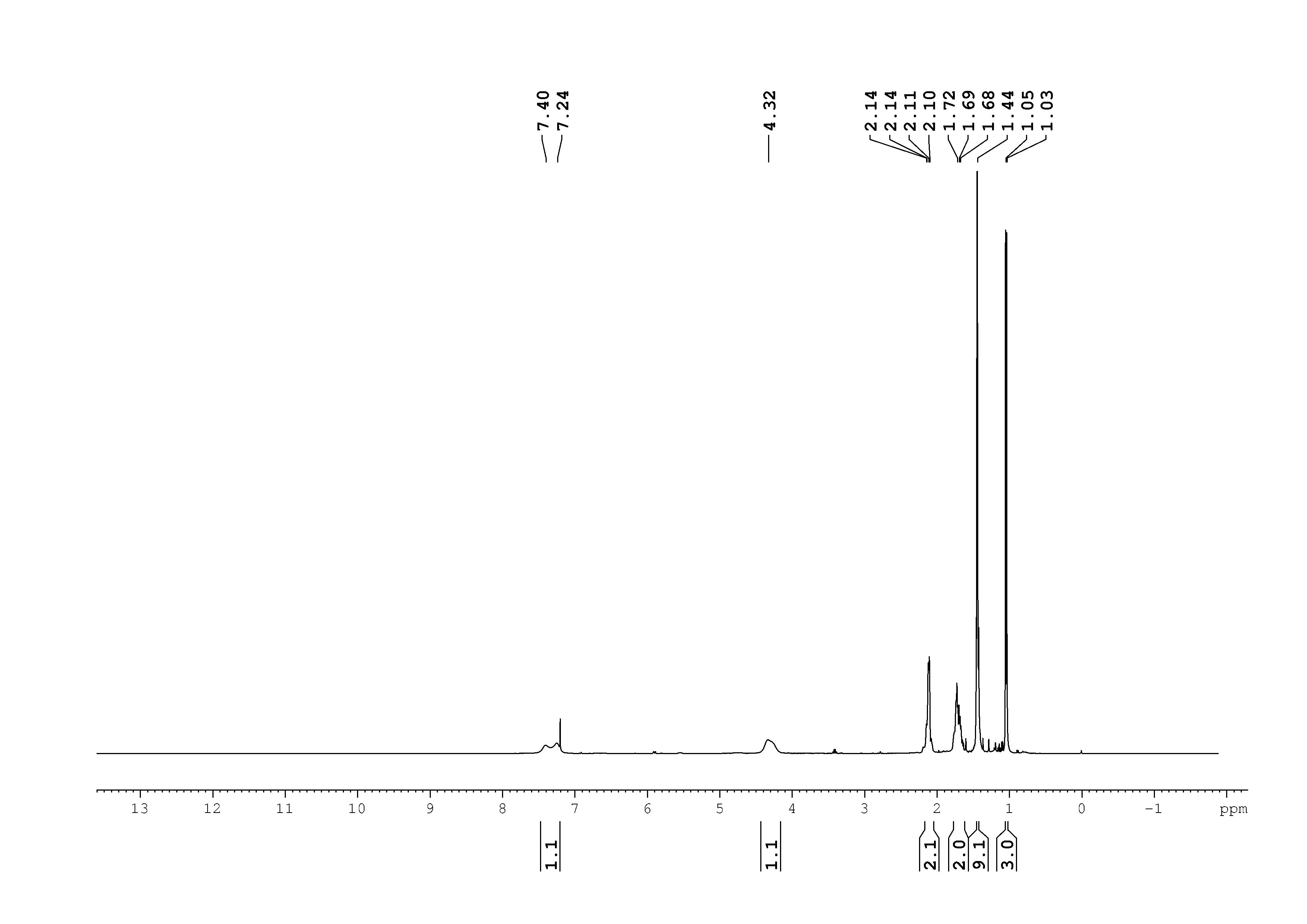
^1^H-NMR spectra of compound **3d**, CDCl_3_, 400.1 MHz


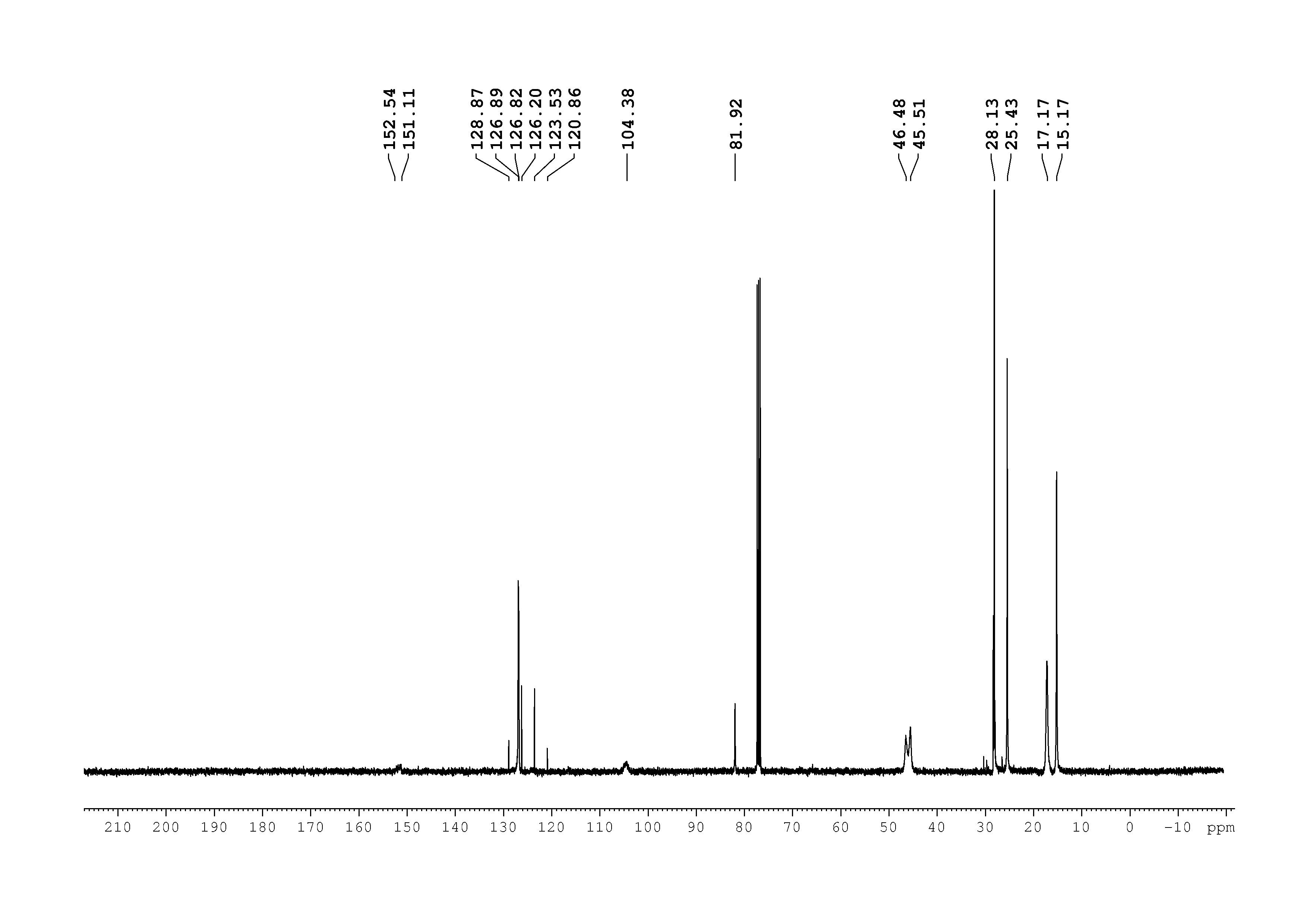
^13^C-NMR spectra of compound **3d**, CDCl_3_, 100.6 MHz


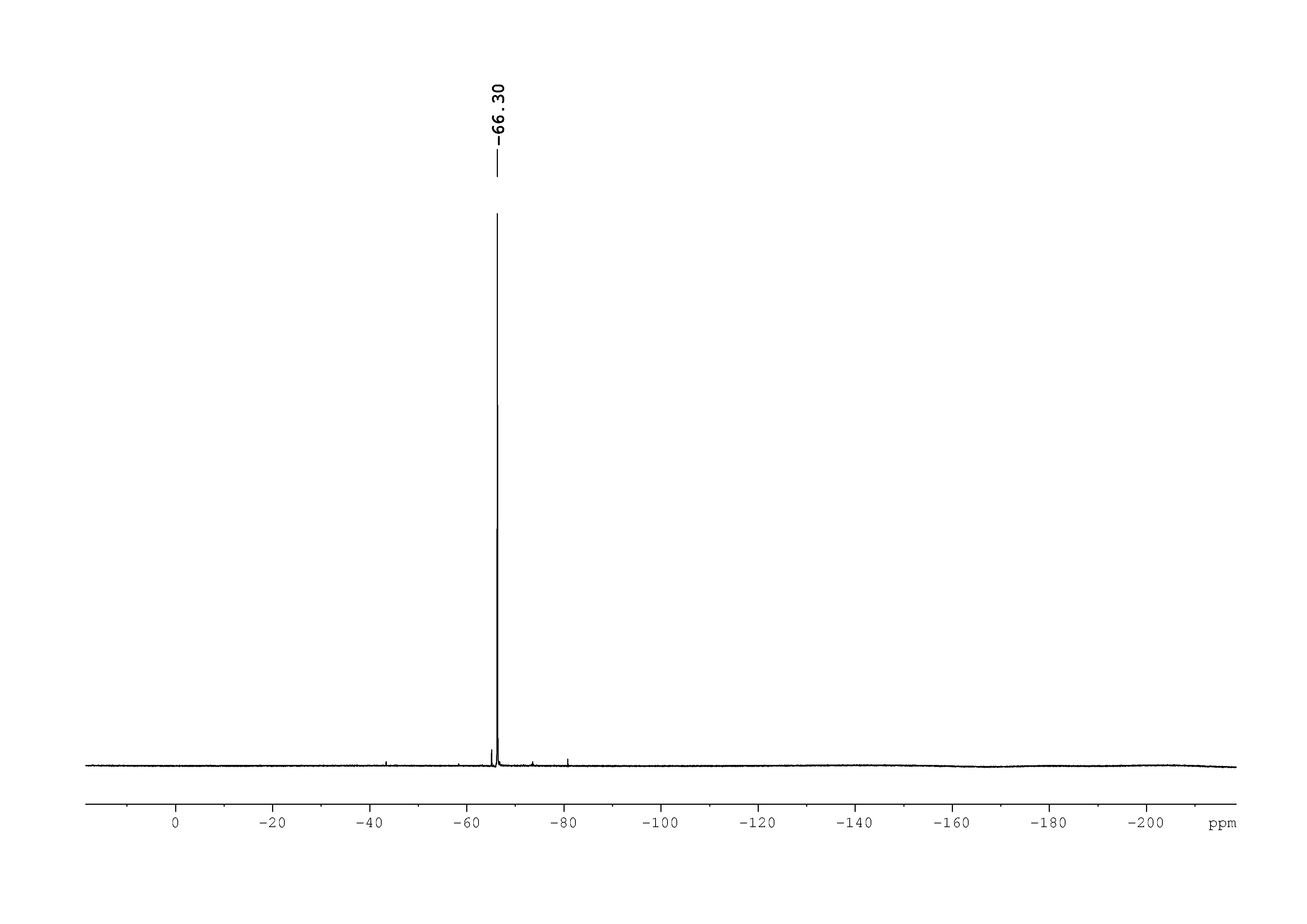
 ^19^F-NMR spectra of compound **3d**, CDCl_3_, 376.5 MHz


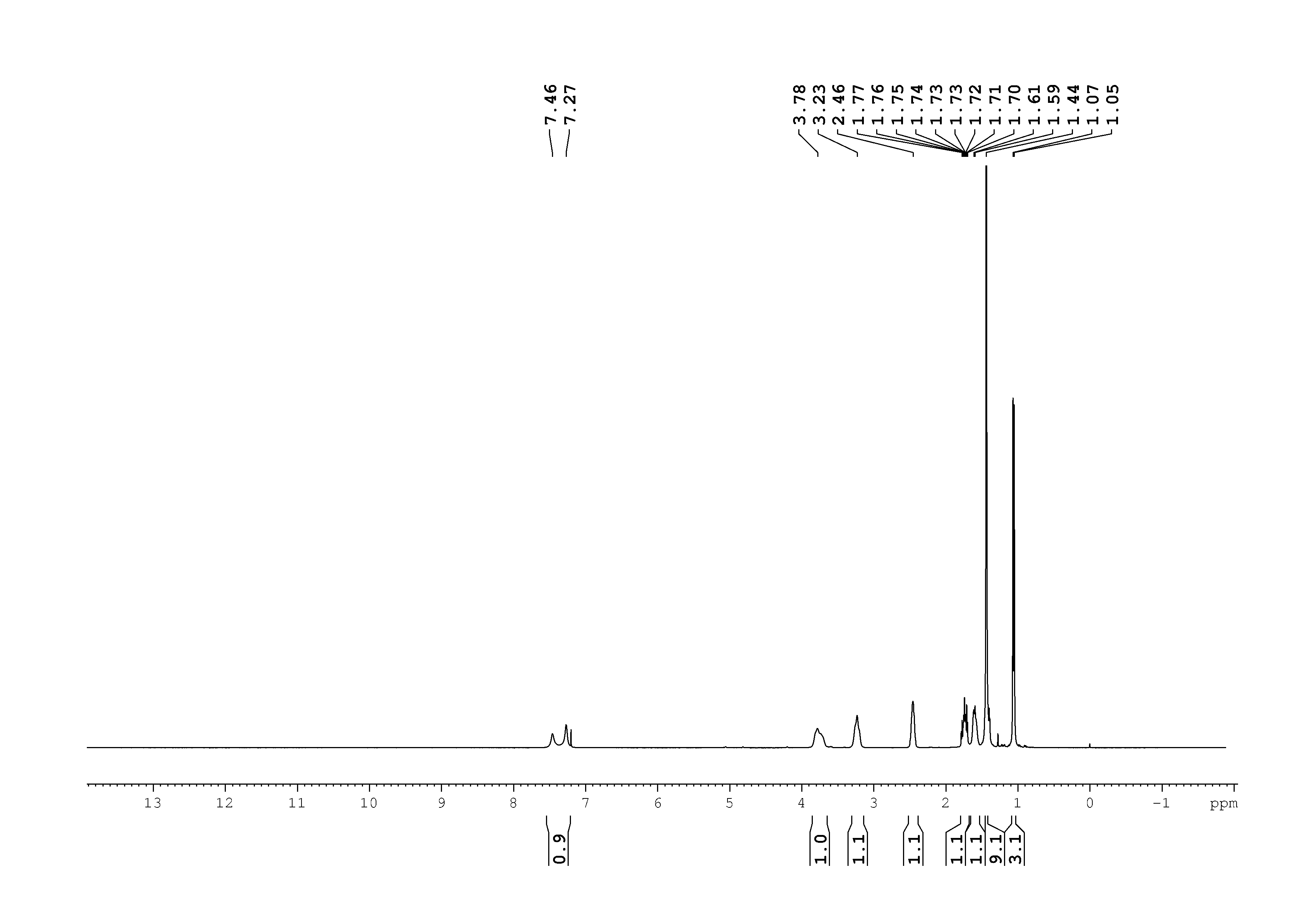
^1^H-NMR spectra of compound **3e**, CDCl_3_, 400.1 MHz


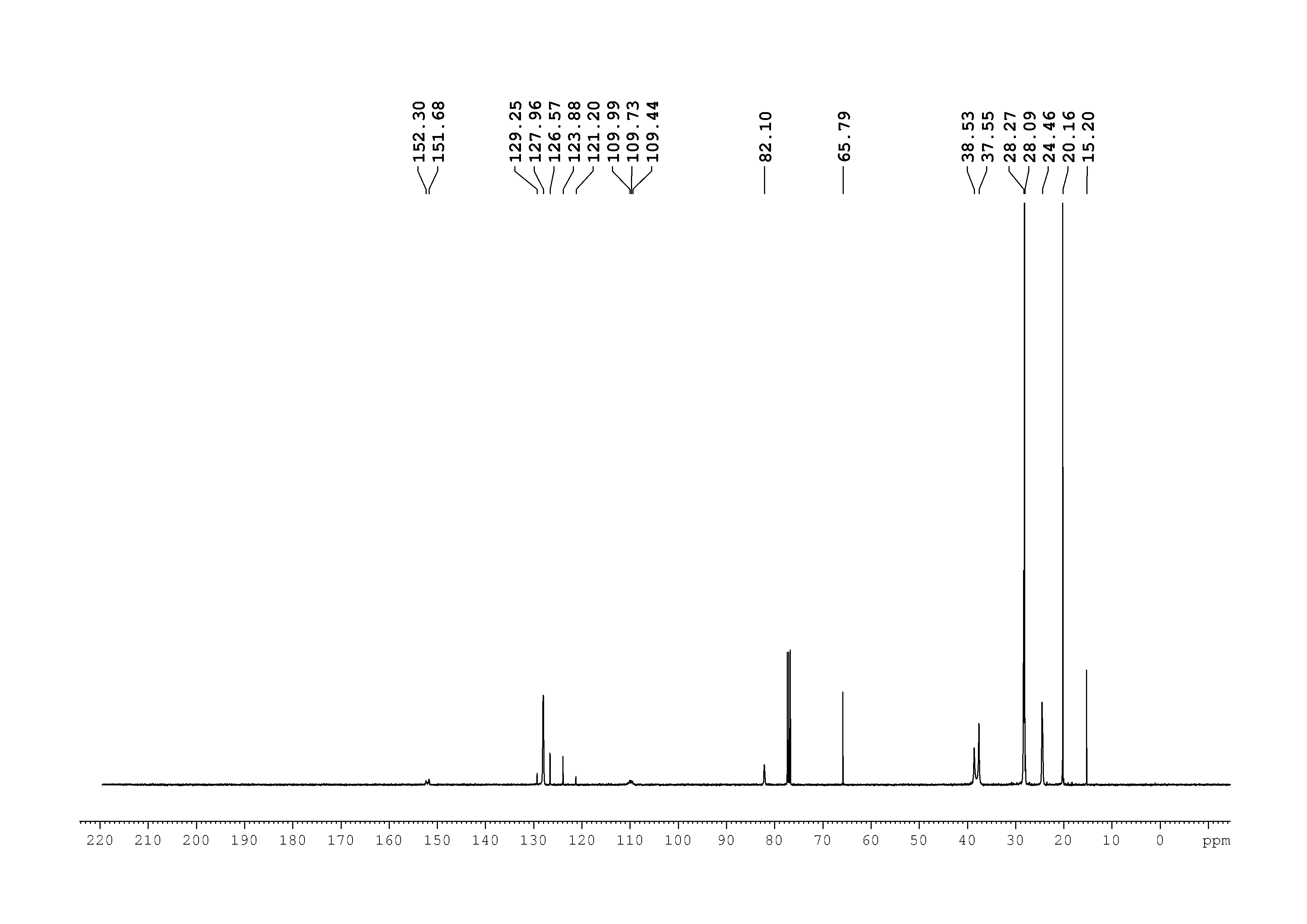
^13^C-NMR spectra of compound **3e**, CDCl_3_, 100.6 MHz


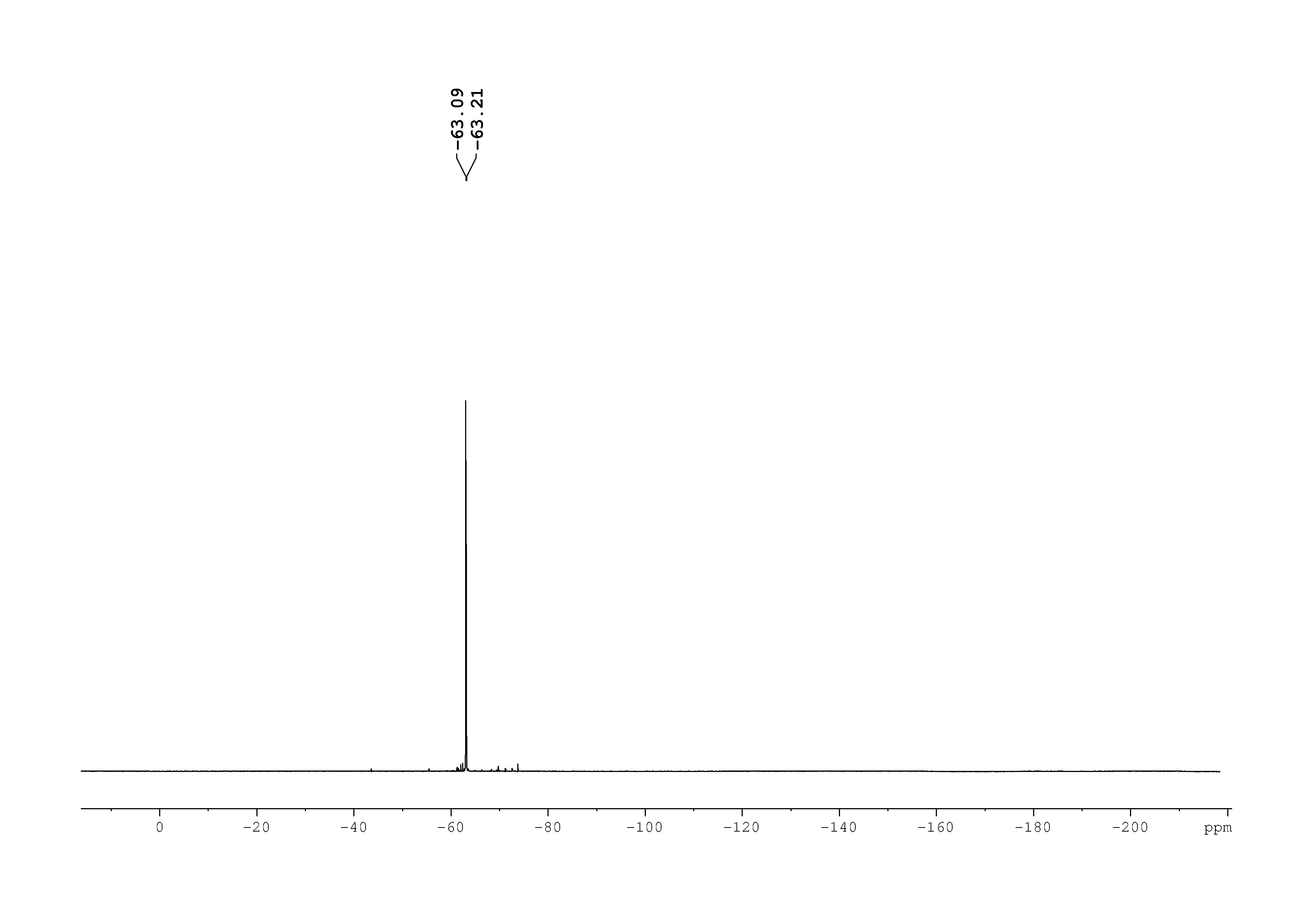
 ^19^F-NMR spectra of compound **3e**, CDCl_3_, 376.5 MHz


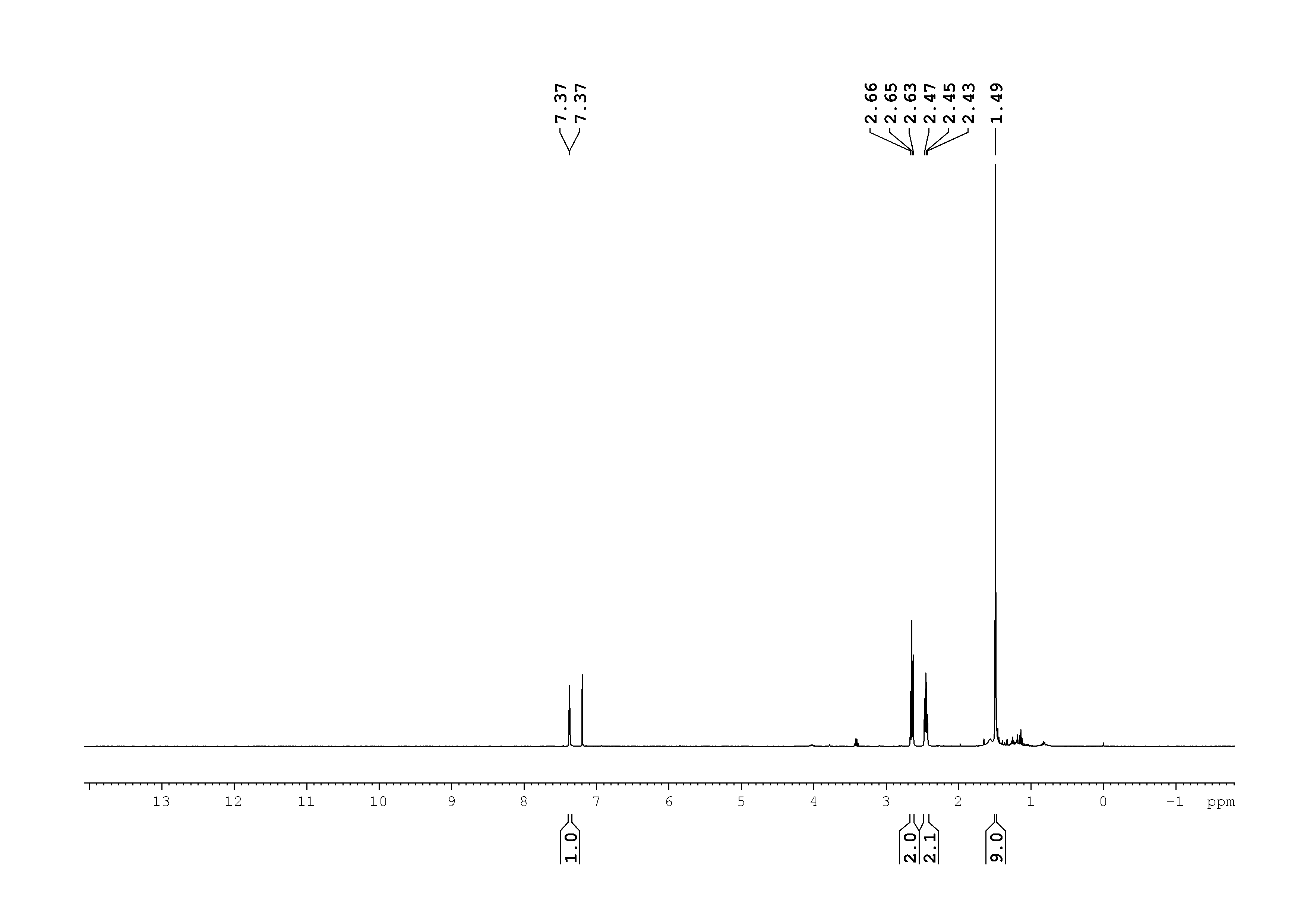
^1^H-NMR spectra of compound **3f**, CDCl_3_, 400.1 MHz


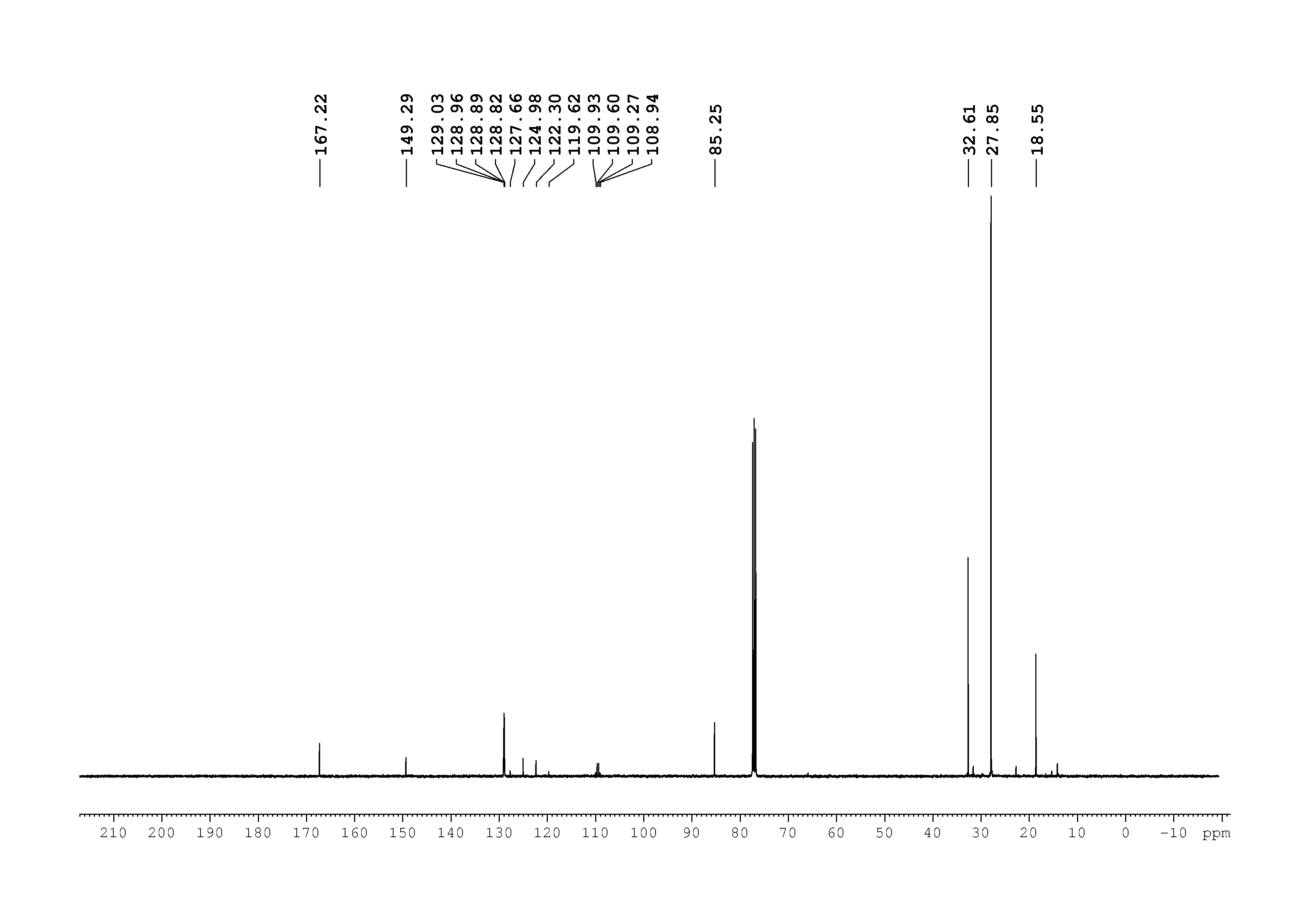
^13^C-NMR spectra of compound **3f**, CDCl_3_, 100.6 MHz


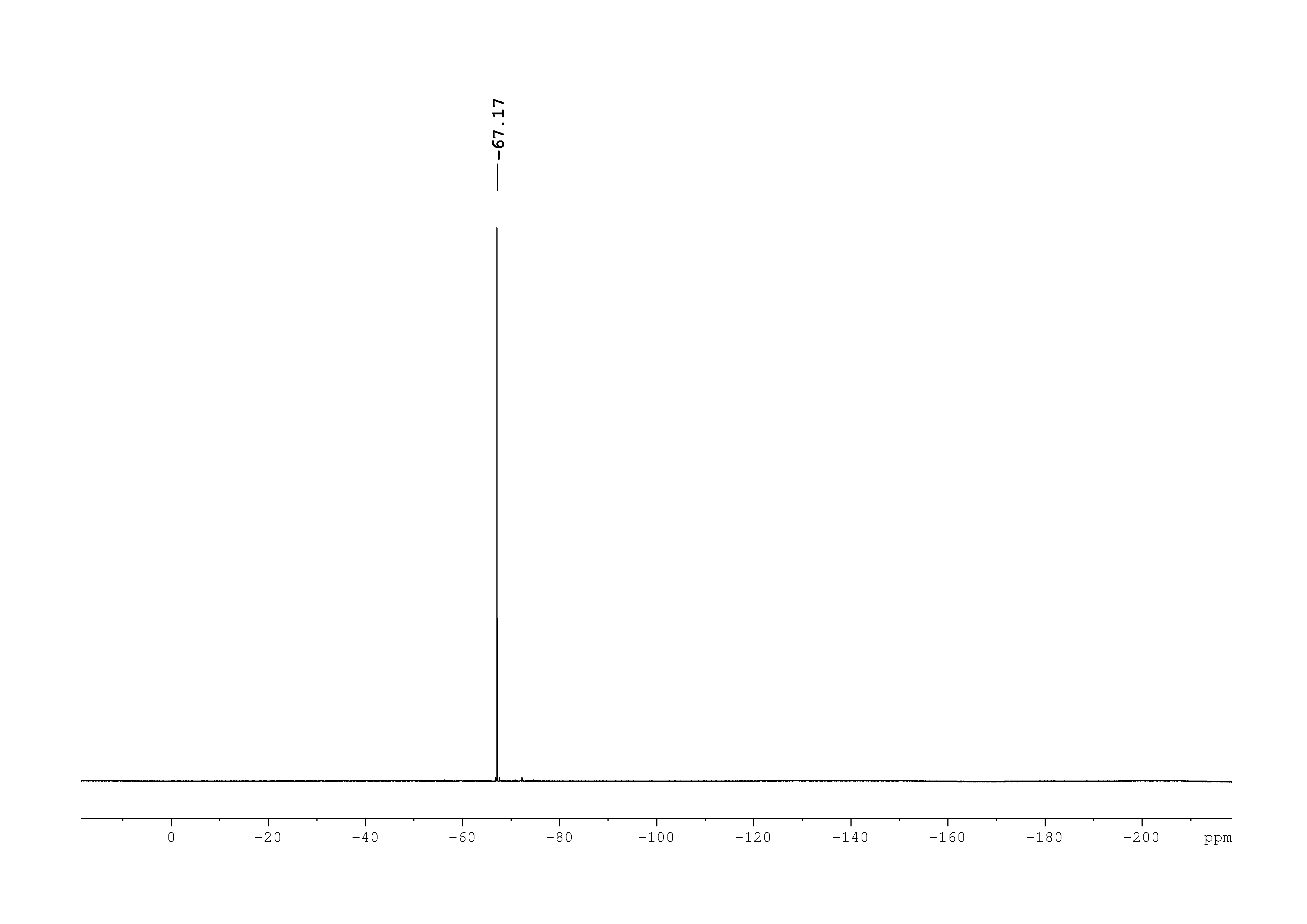
 ^19^F-NMR spectra of compound **3f**, CDCl_3_, 376.5 MHz


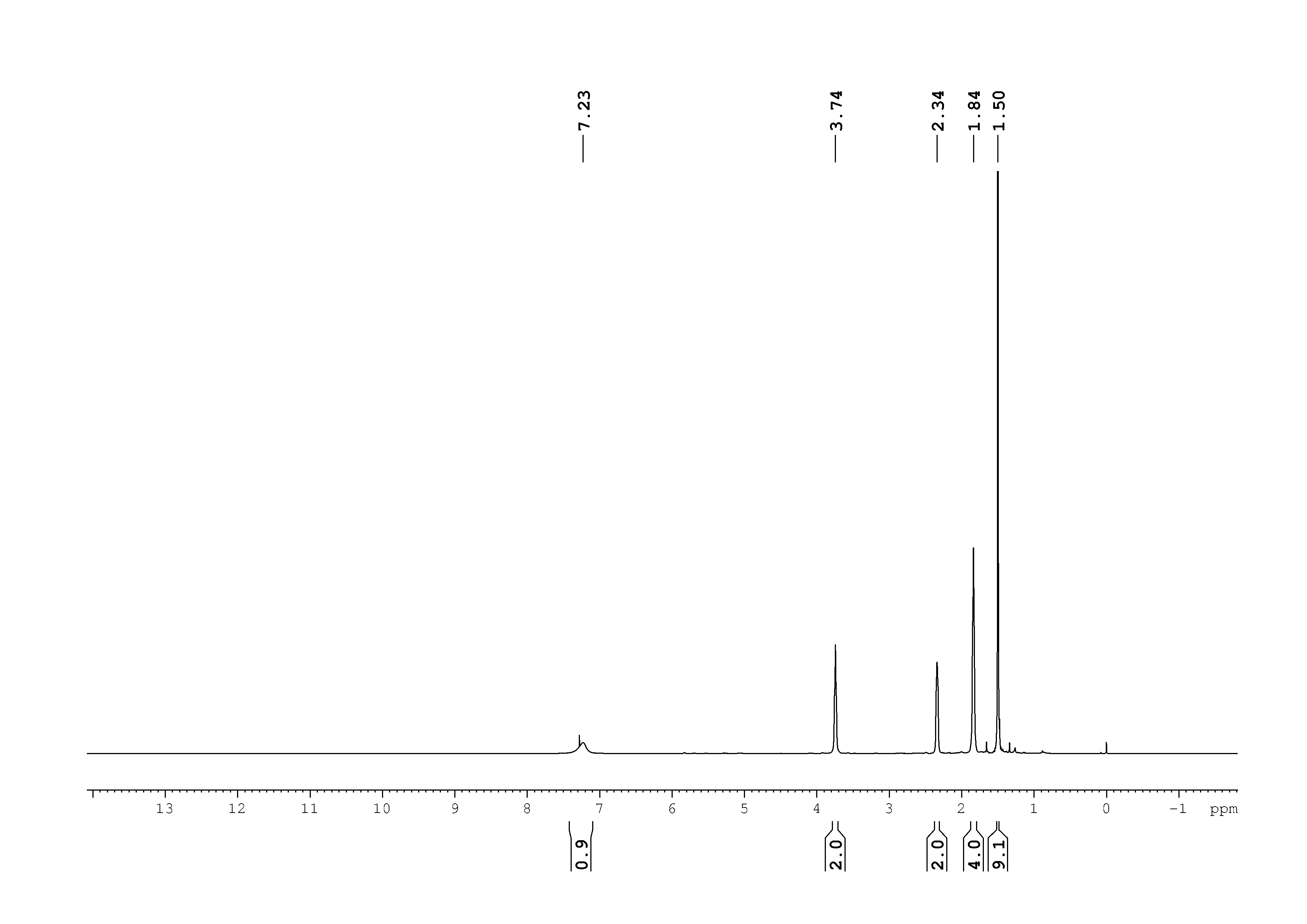
^1^H-NMR spectra of compound **3g**, CDCl_3_, 400.1 MHz


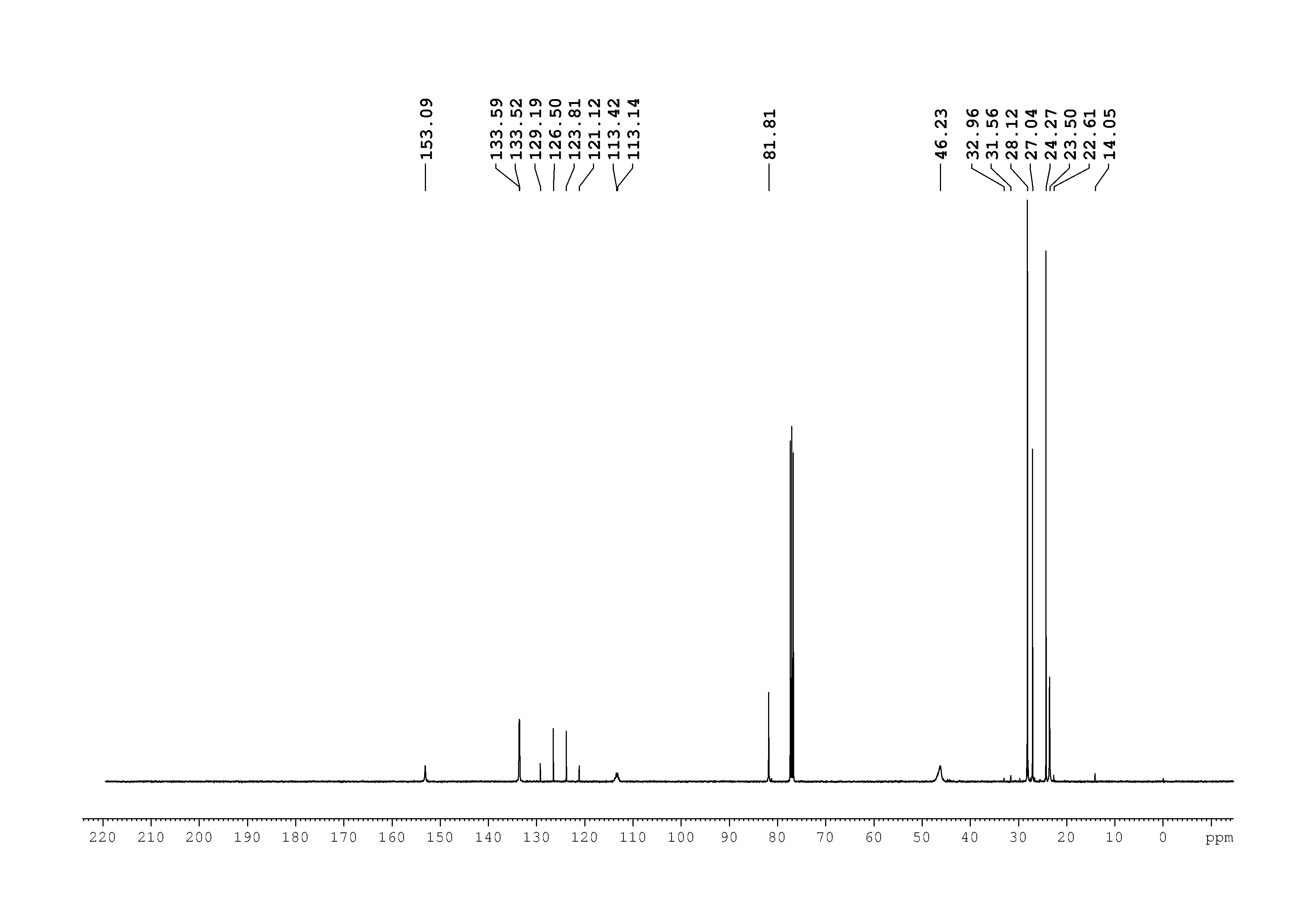
^13^C-NMR spectra of compound **3g**, CDCl_3_, 100.6 MHz


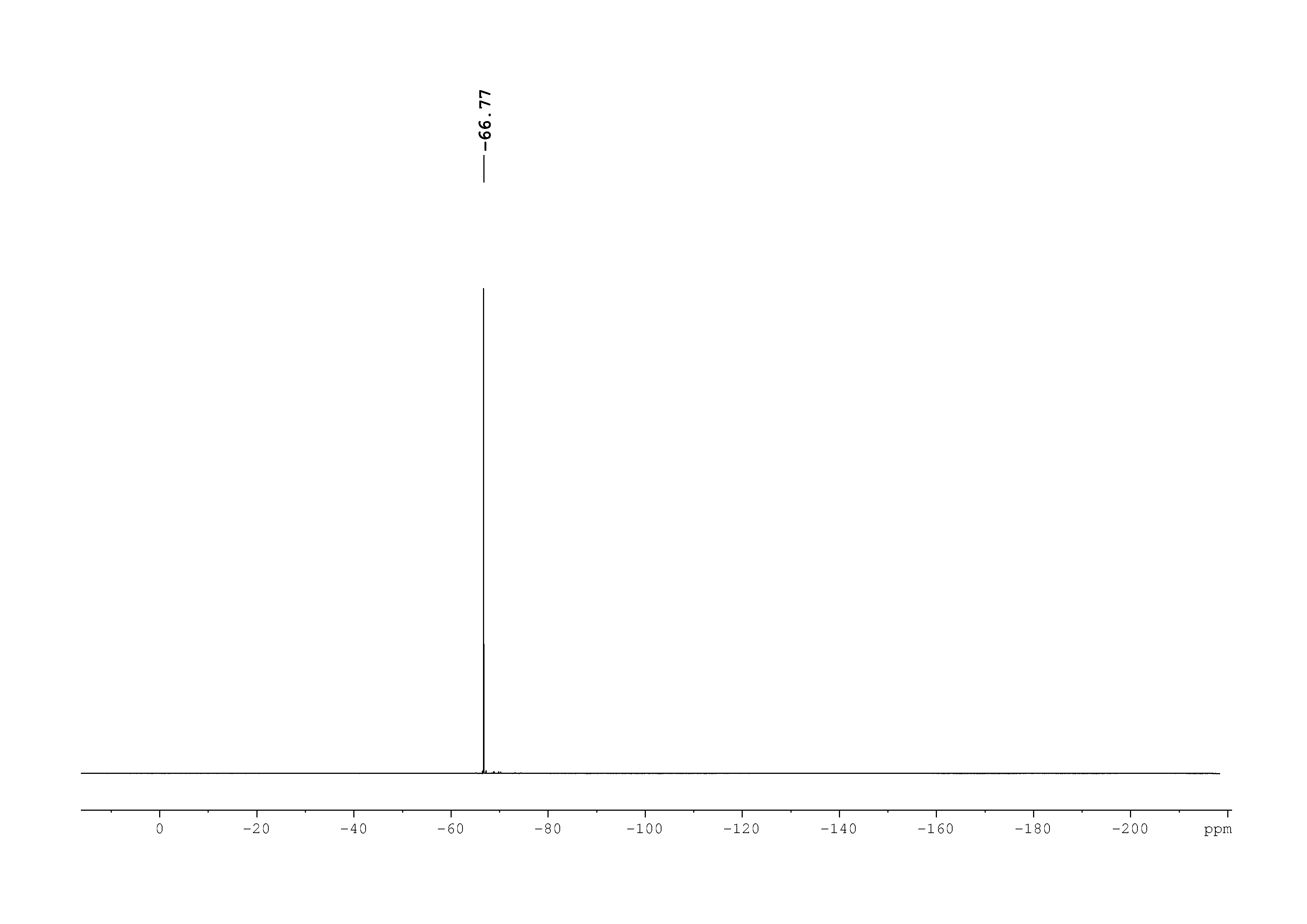
 ^19^F-NMR spectra of compound **3g**, CDCl_3_, 376.5 MHz

**
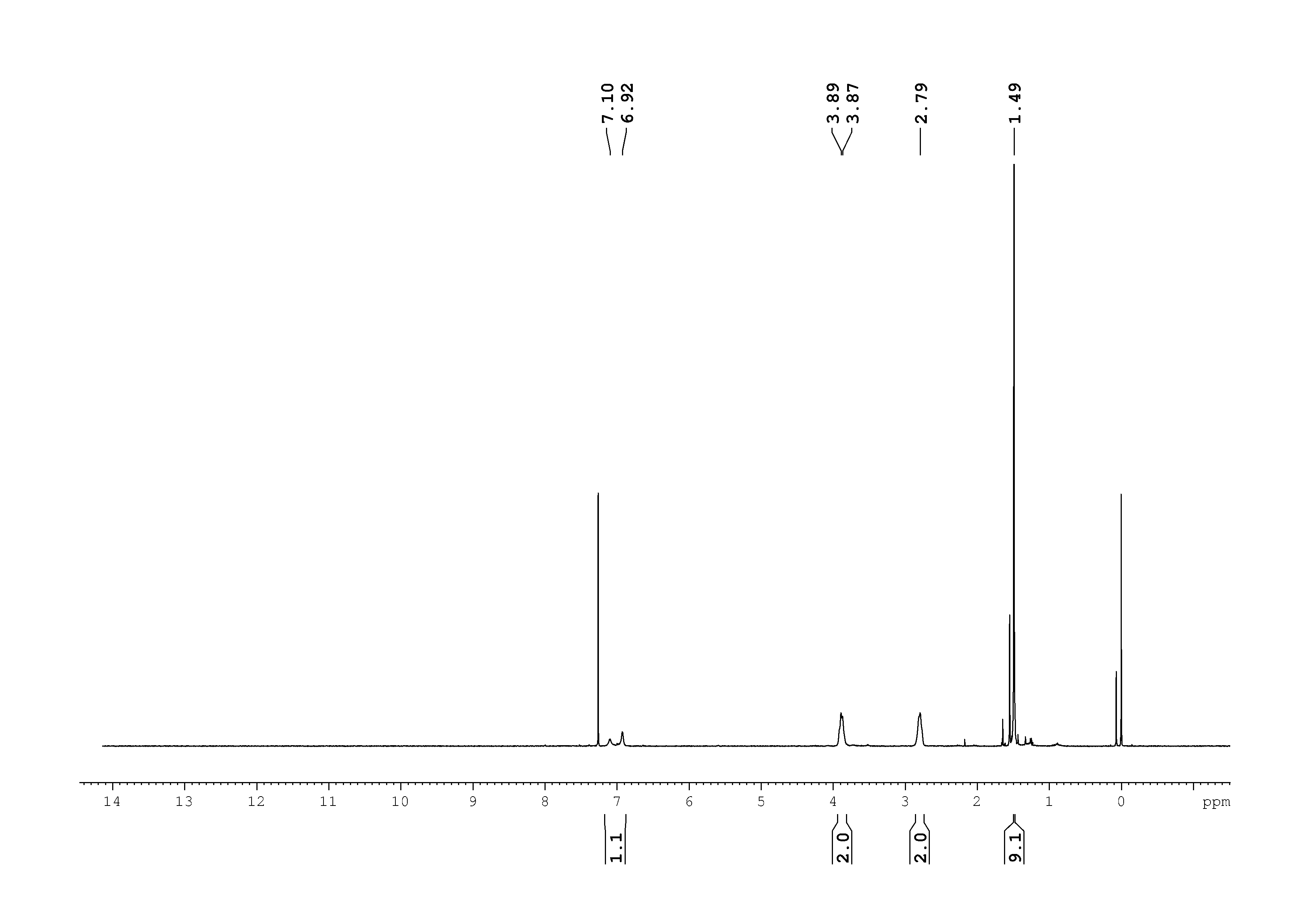
**^1^H-NMR spectra of compound **3h**, CDCl_3_, 400.1 MHz


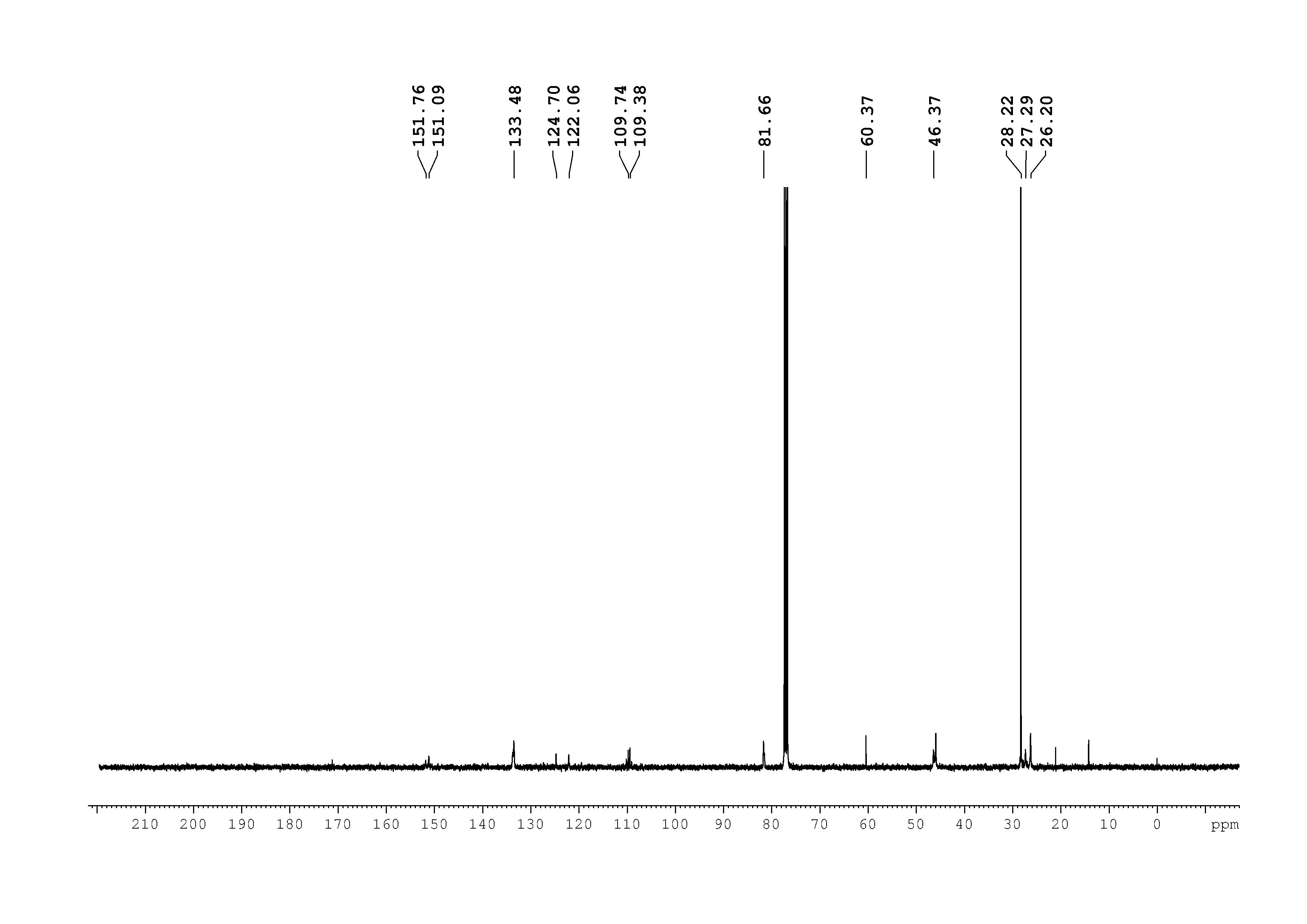
^13^C-NMR spectra of compound **3h**, CDCl_3_, 100.6 MHz


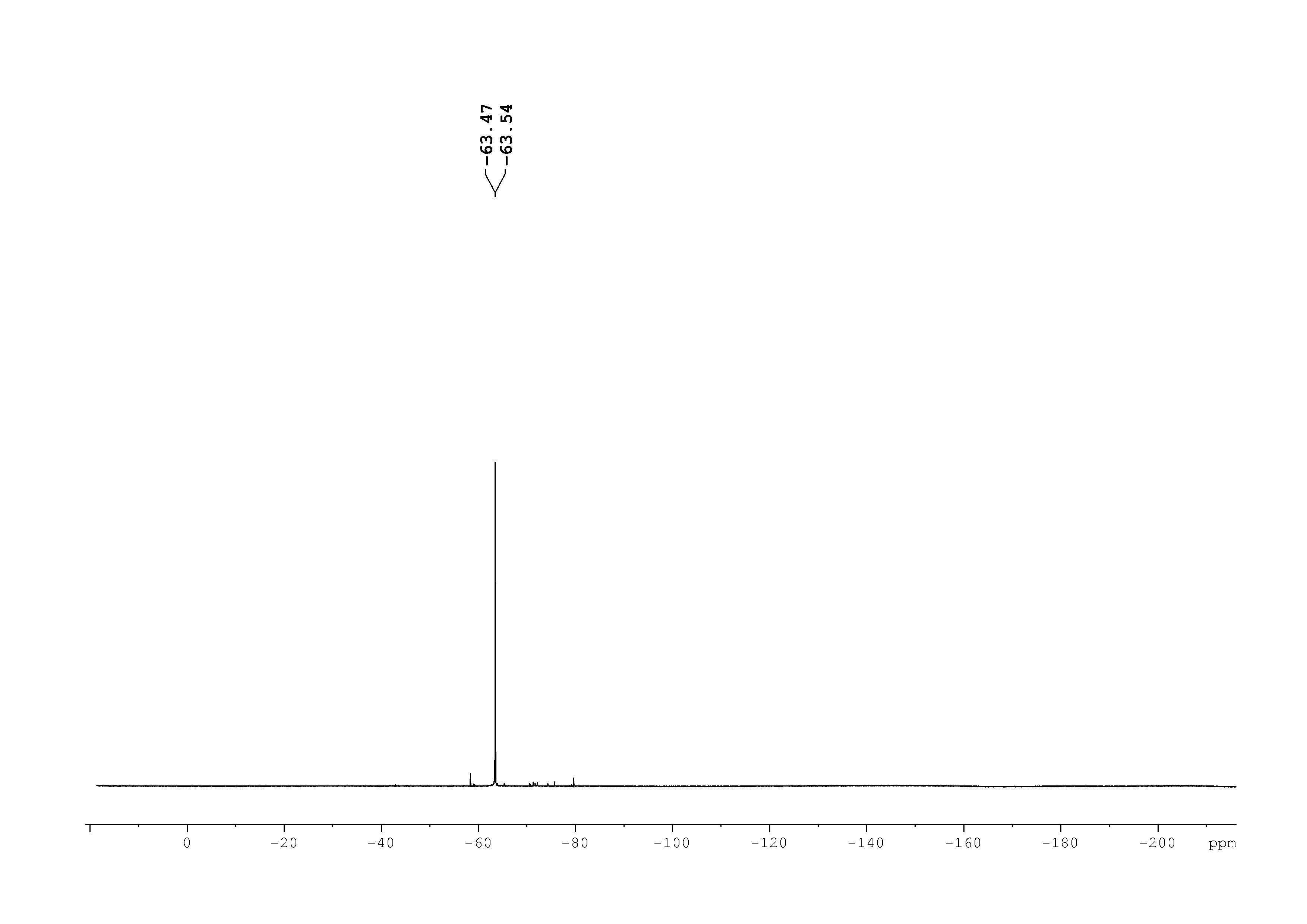

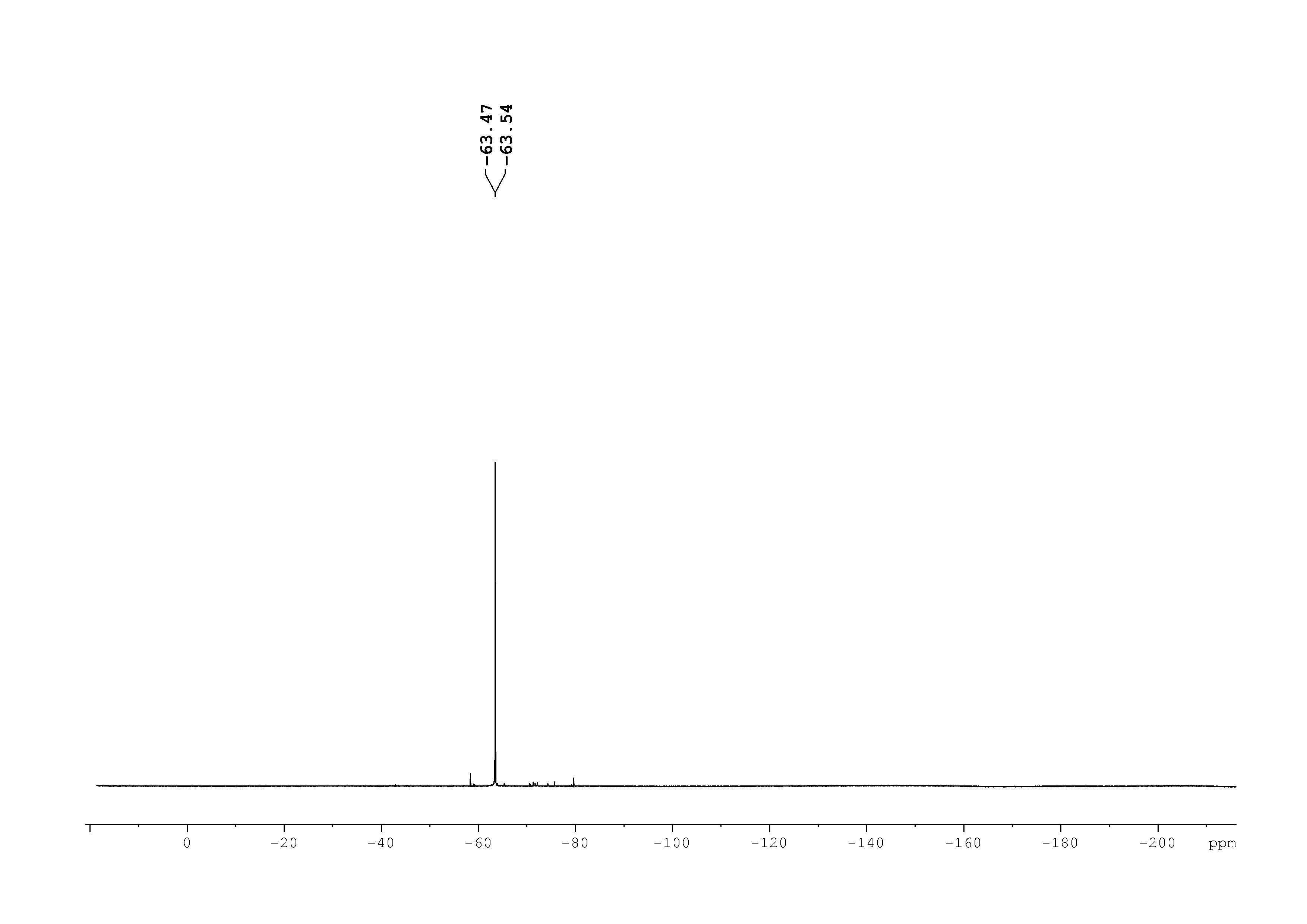
 ^19^F-NMR spectra of compound **3h**, CDCl_3_, 376.5 MHz


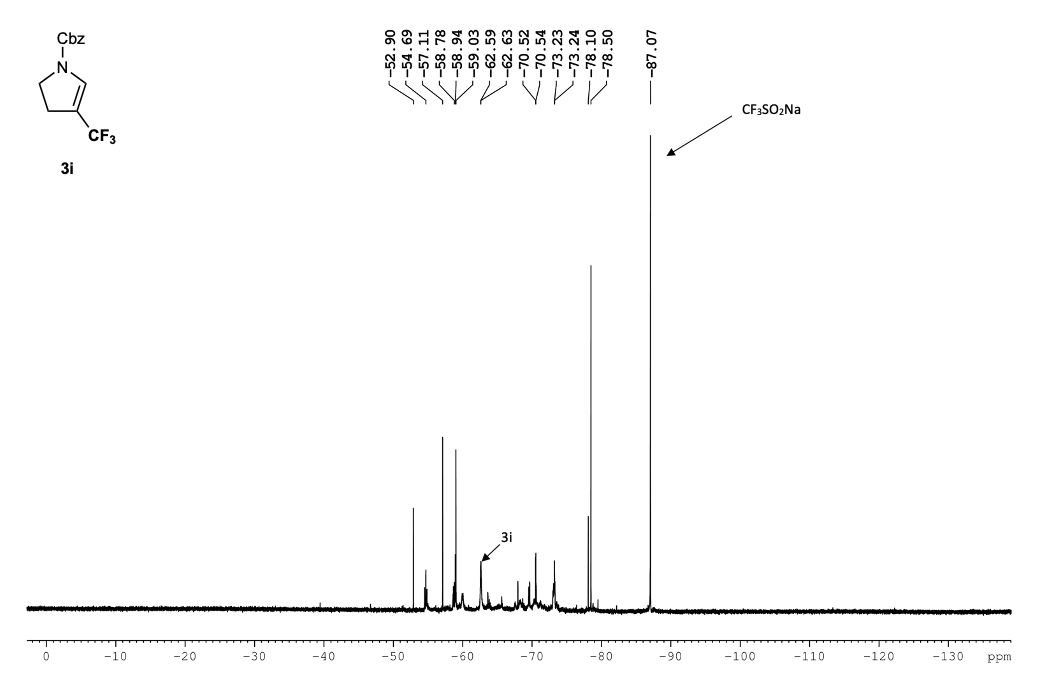
^19^F NMR spectra (376.5 MHz) of crude reaction mixture: **3i** (traces), CF_3_SO_2_Na and a lot of side products


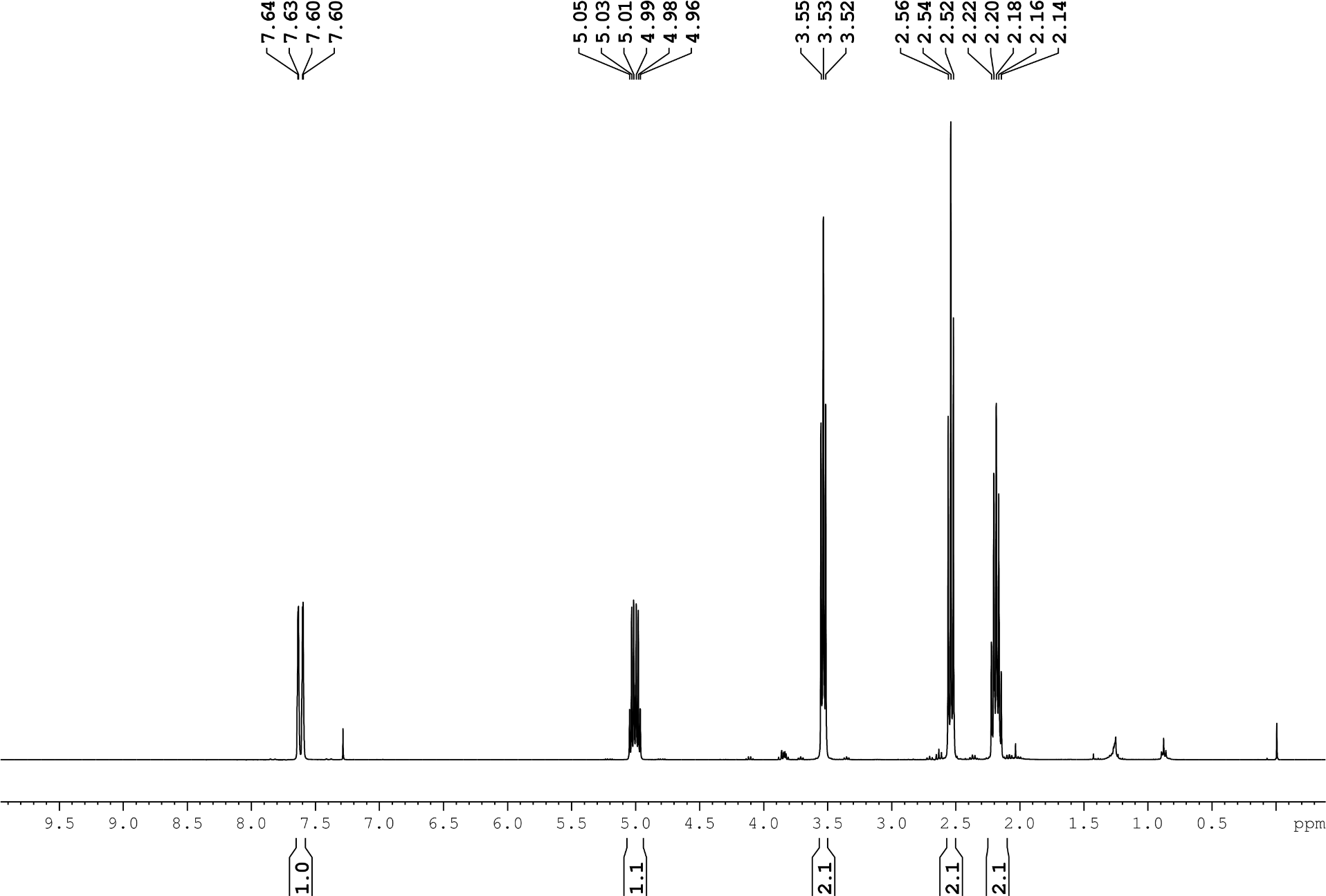
^1^H-NMR spectra of compound **3j**, CDCl_3_, 400.1 MHz


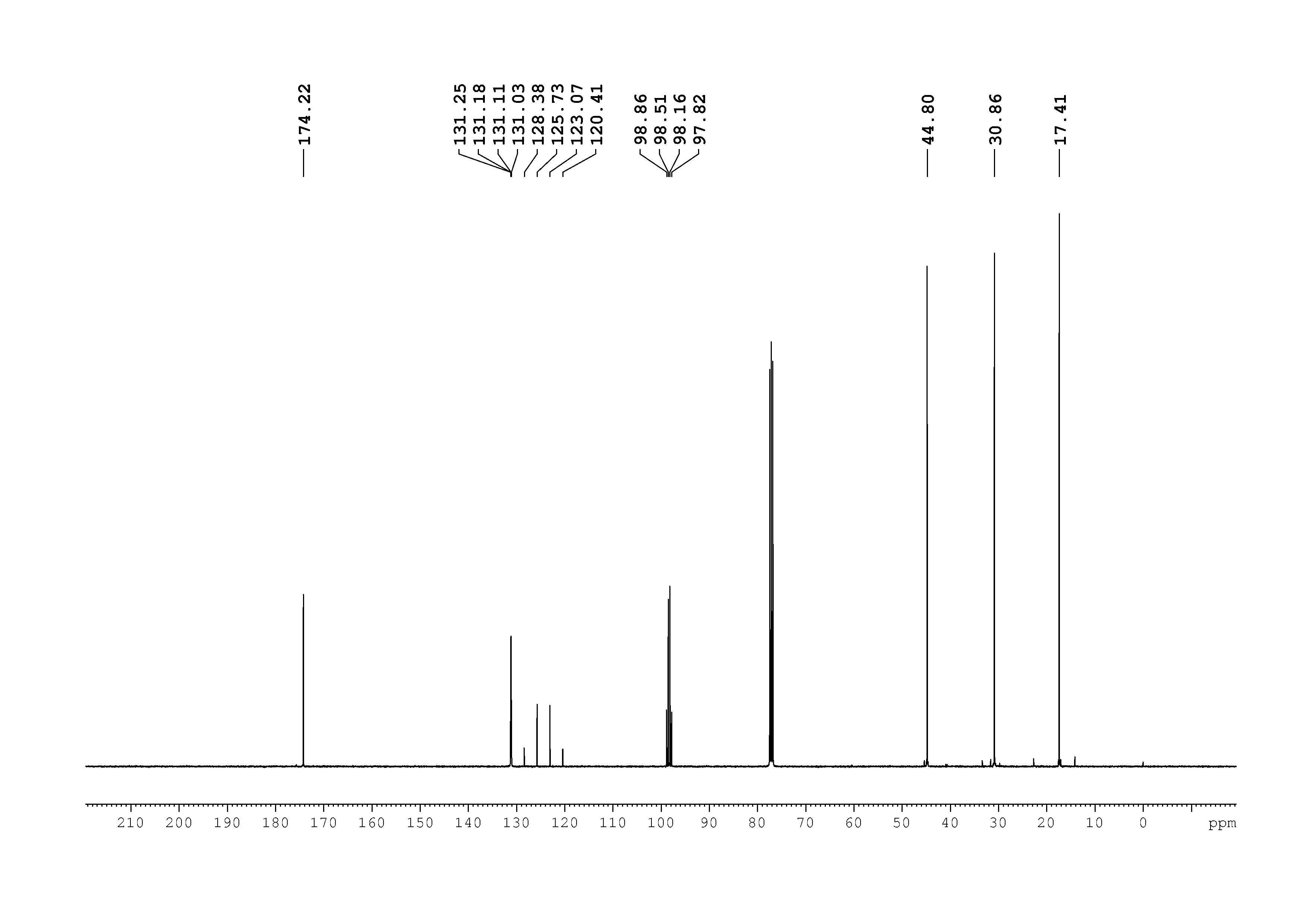
^13^C-NMR spectra of compound **3j**, CDCl_3_, 100.6 MHz


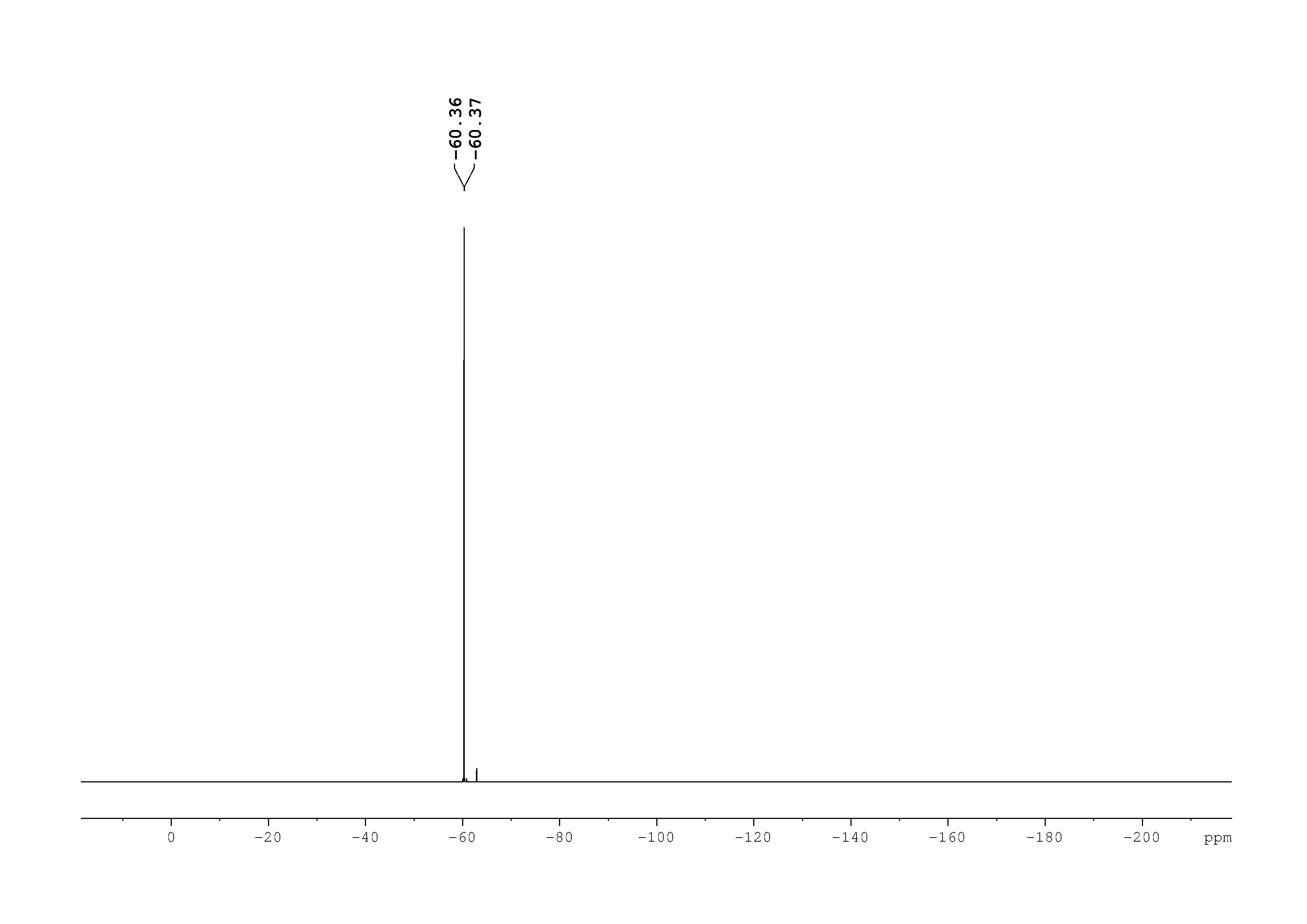
^19^F-NMR spectra of compound **3j**, CDCl_3_, 376.5 MHz


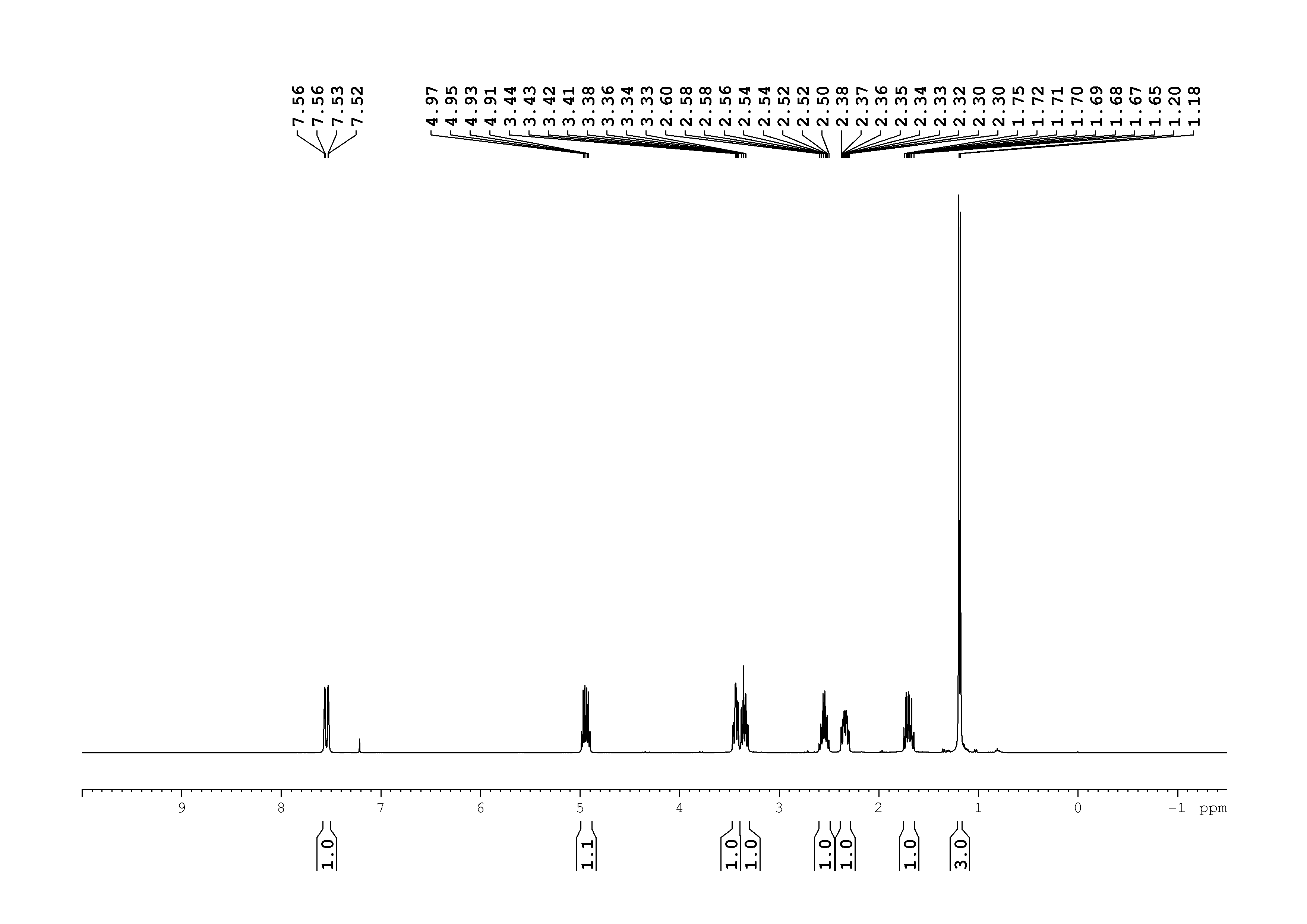
^1^H-NMR spectra of compound **3k**, CDCl_3_, 400.1 MHz


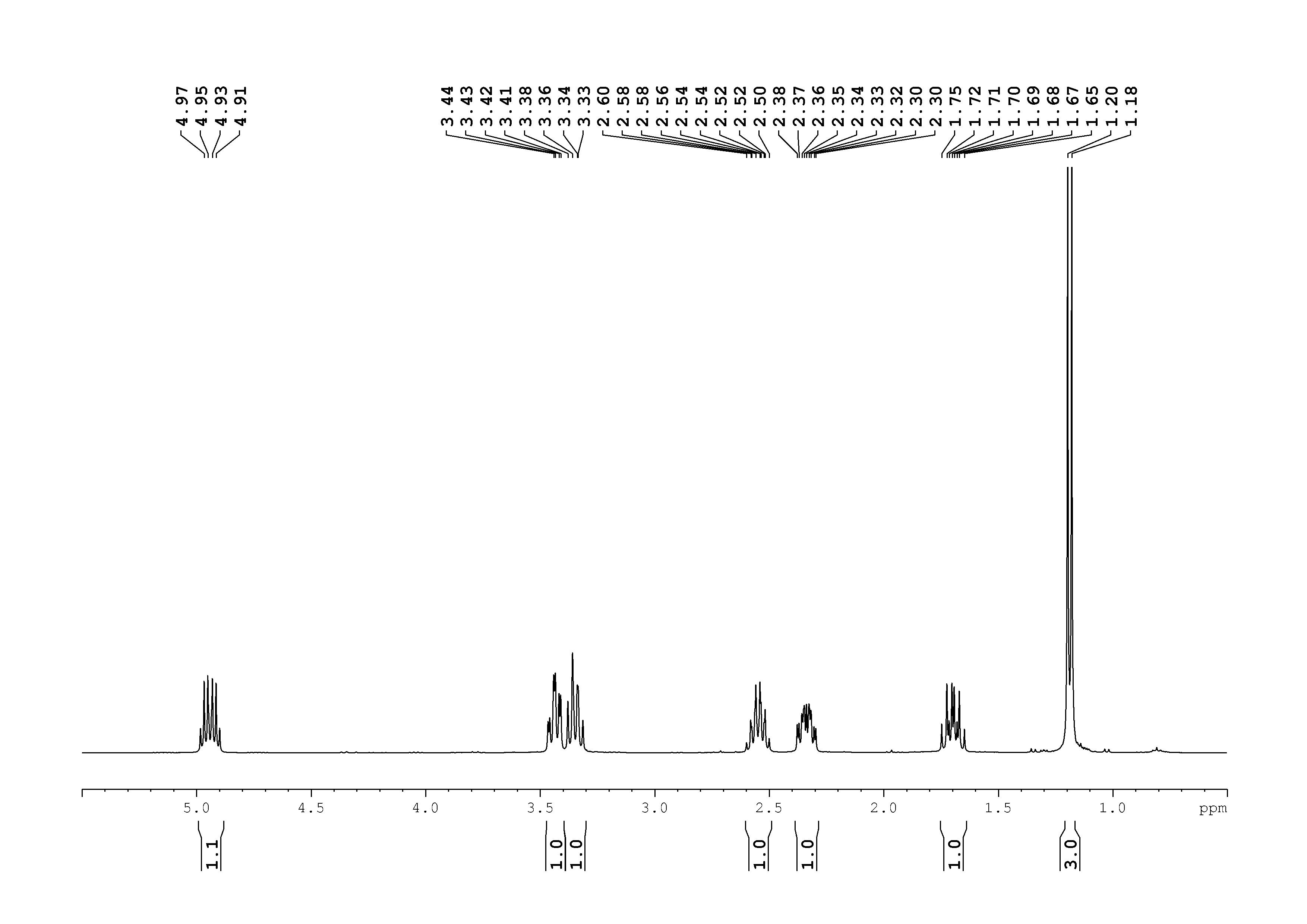
^1^H-NMR spectra of compound **3k**, CDCl_3_, 400.1 MHz

^13^C-NMR spectra of compound **3k**, CDCl_3_, 100.6 MHz


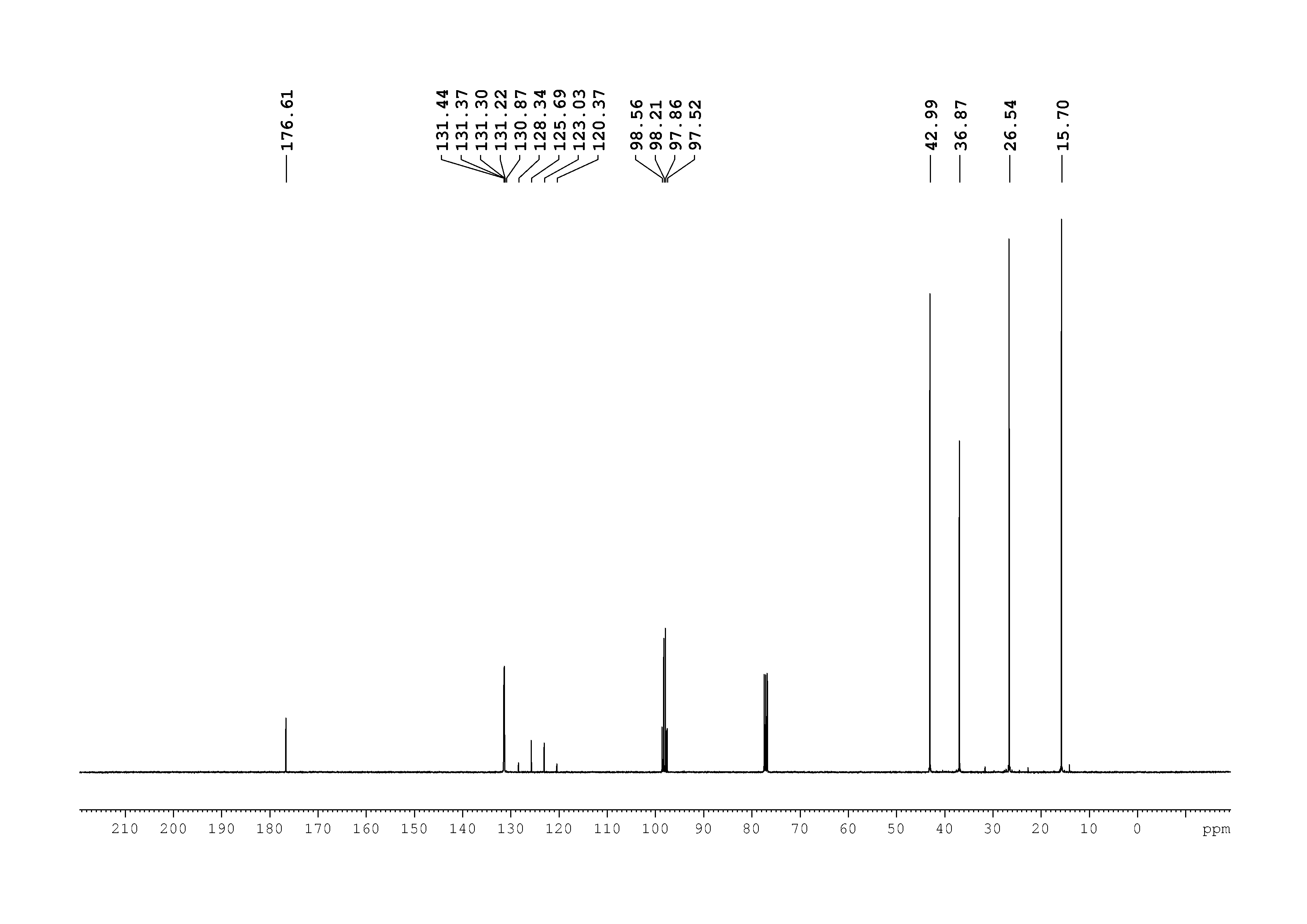


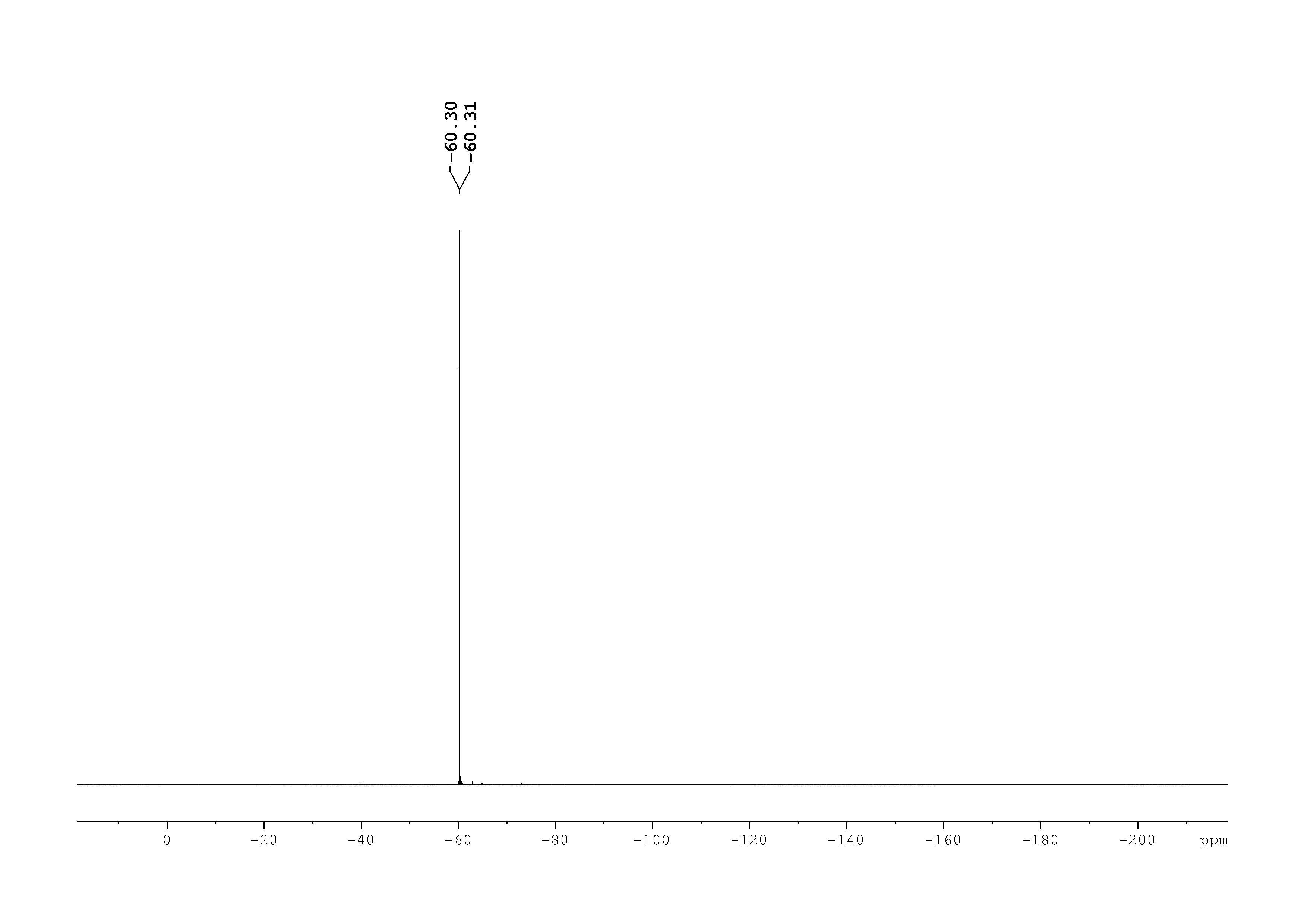
^19^F-NMR spectra of compound **3k**, CDCl_3_, 376.5 MHz


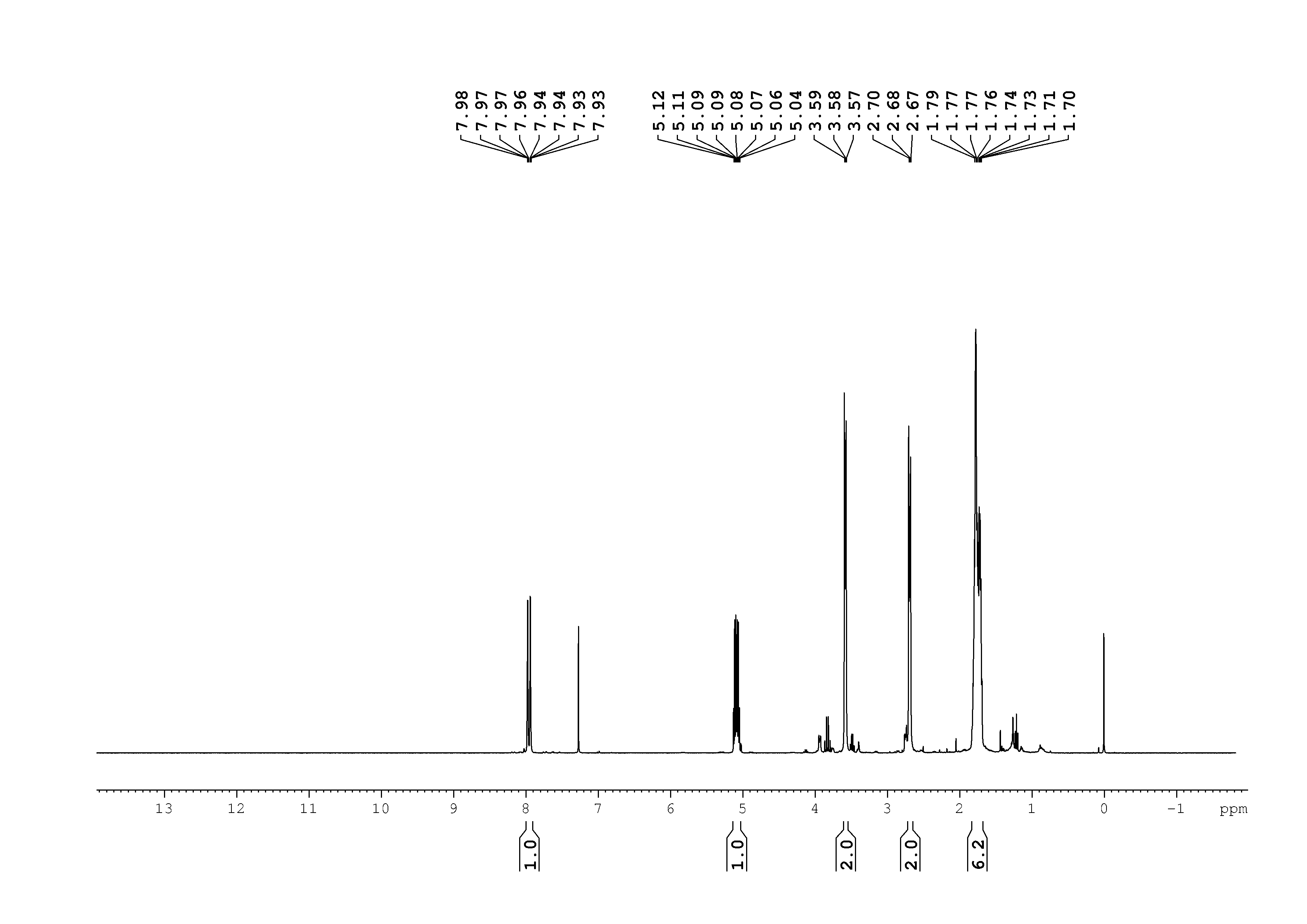
^1^H-NMR spectra of compound **3l**, CDCl_3_, 400.1 MHz


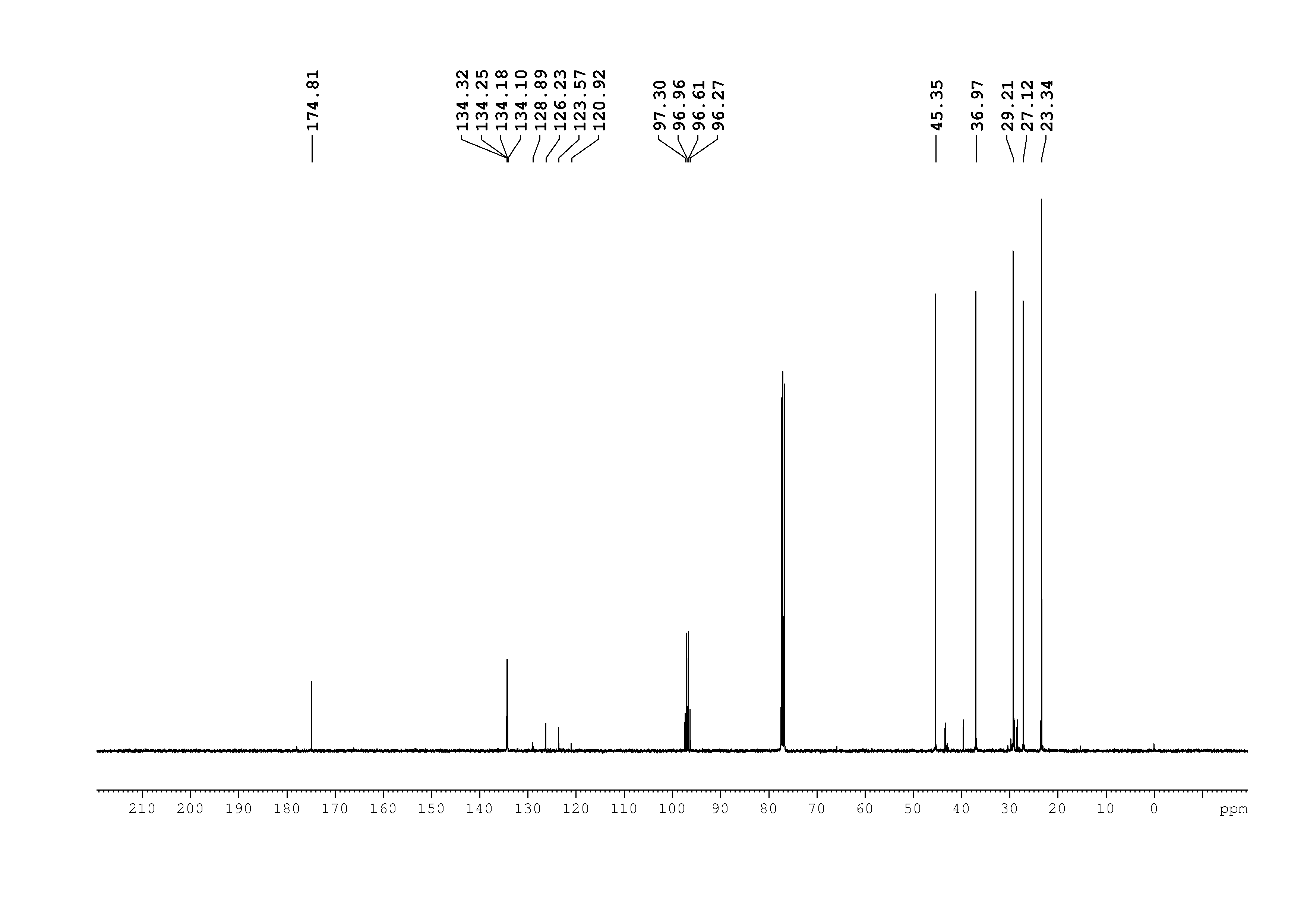
^13^C-NMR spectra of compound **3l**, CDCl_3_, 100.6 MHz

^19^F-NMR spectra of compound **3l**, CDCl_3_, 376.5 MHz


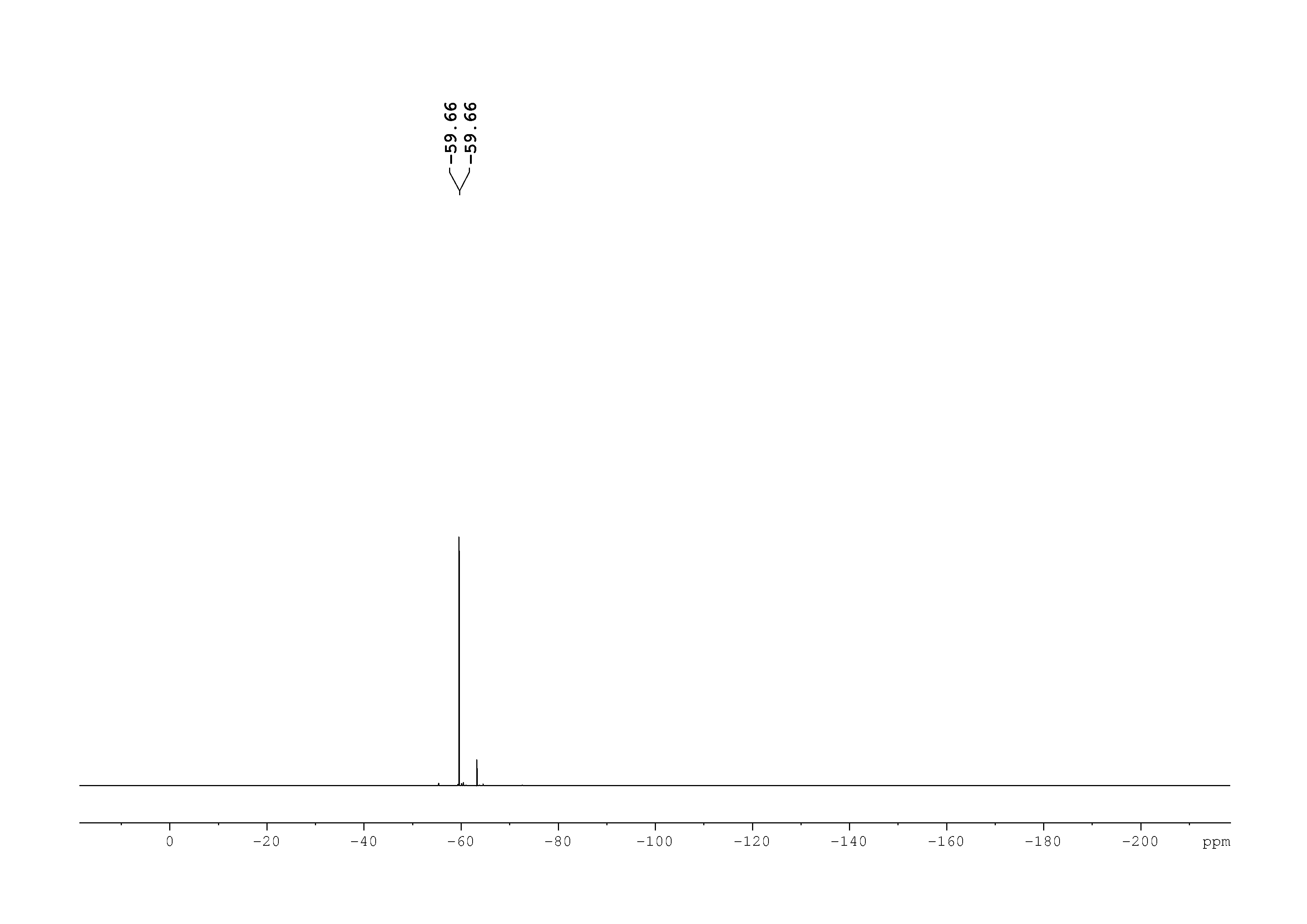


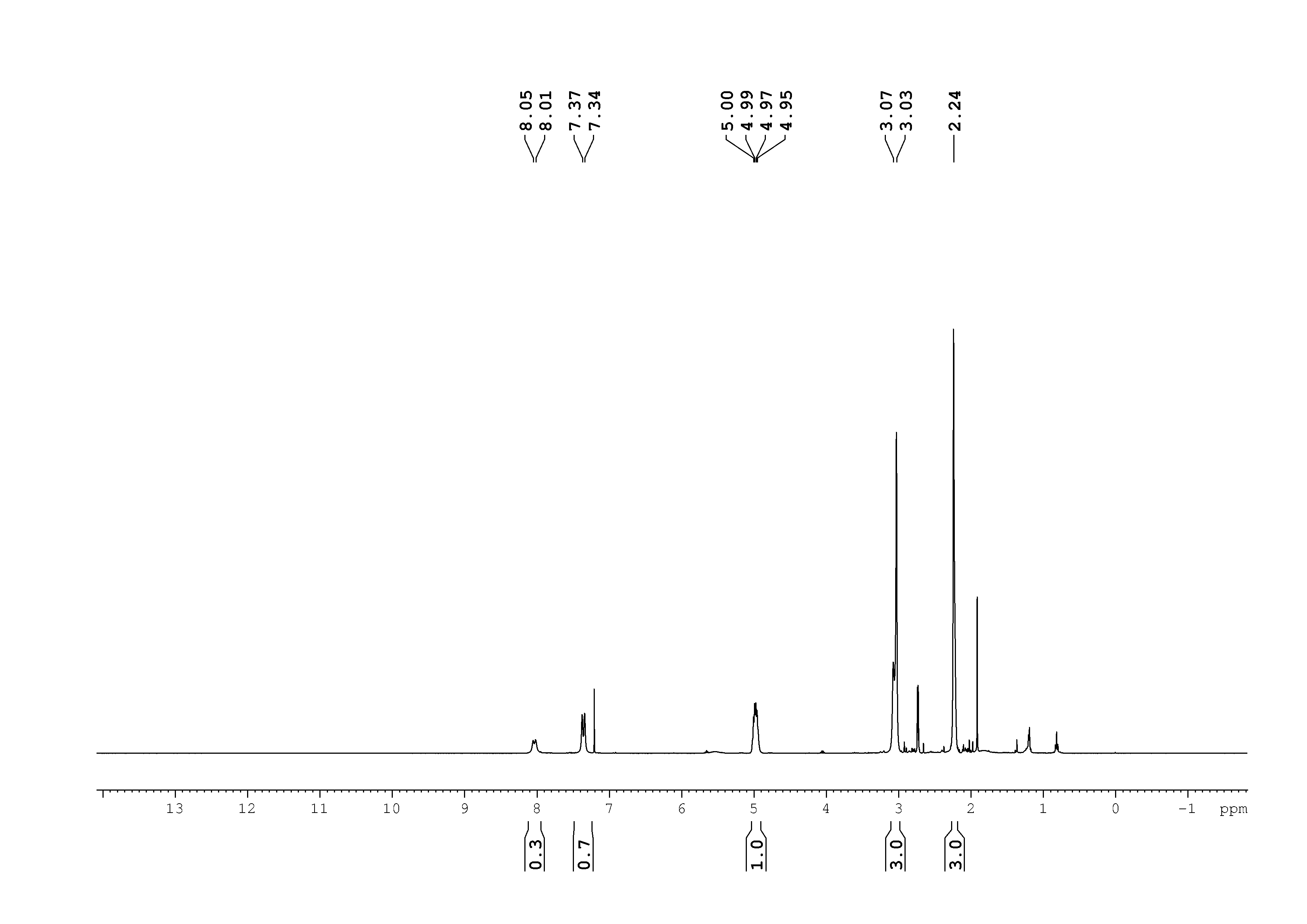
^1^H-NMR spectra of compound **3m**, CDCl_3_, 400.1 MHz


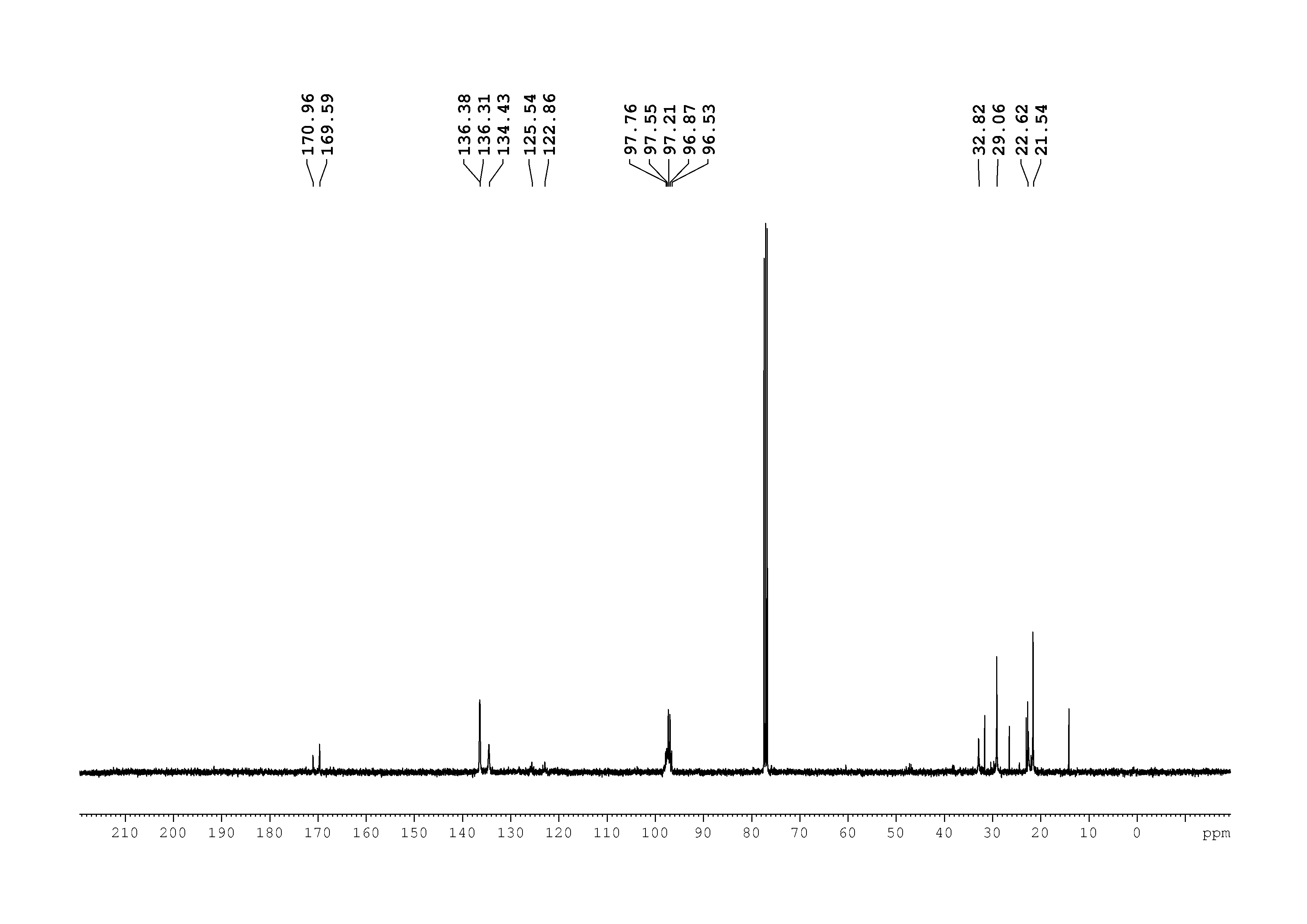
^13^C-NMR spectra of compound **3m**, CDCl_3_, 100.6 MHz


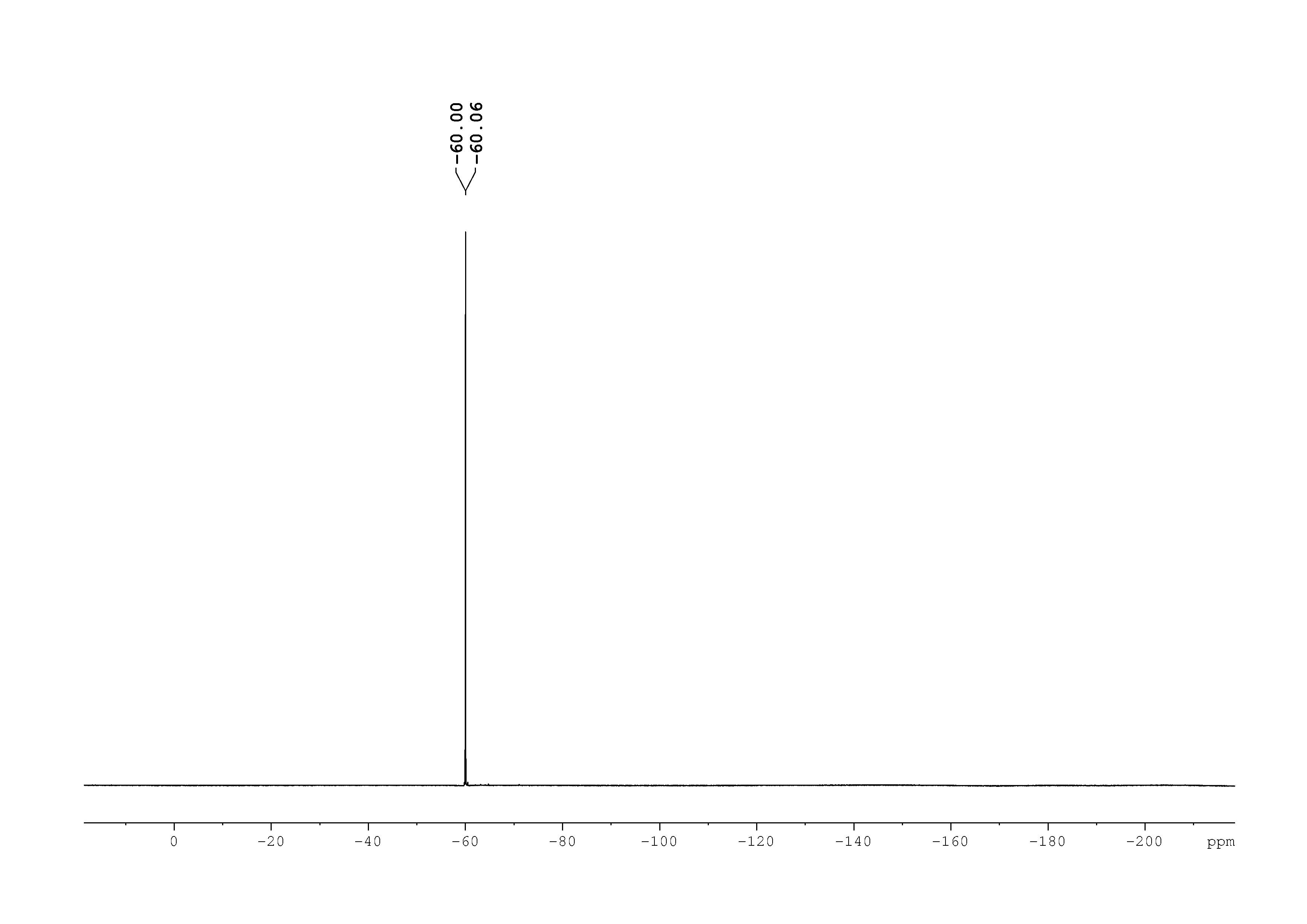
^19^F-NMR spectra of compound **3m**, CDCl_3_, 376.5 MHz


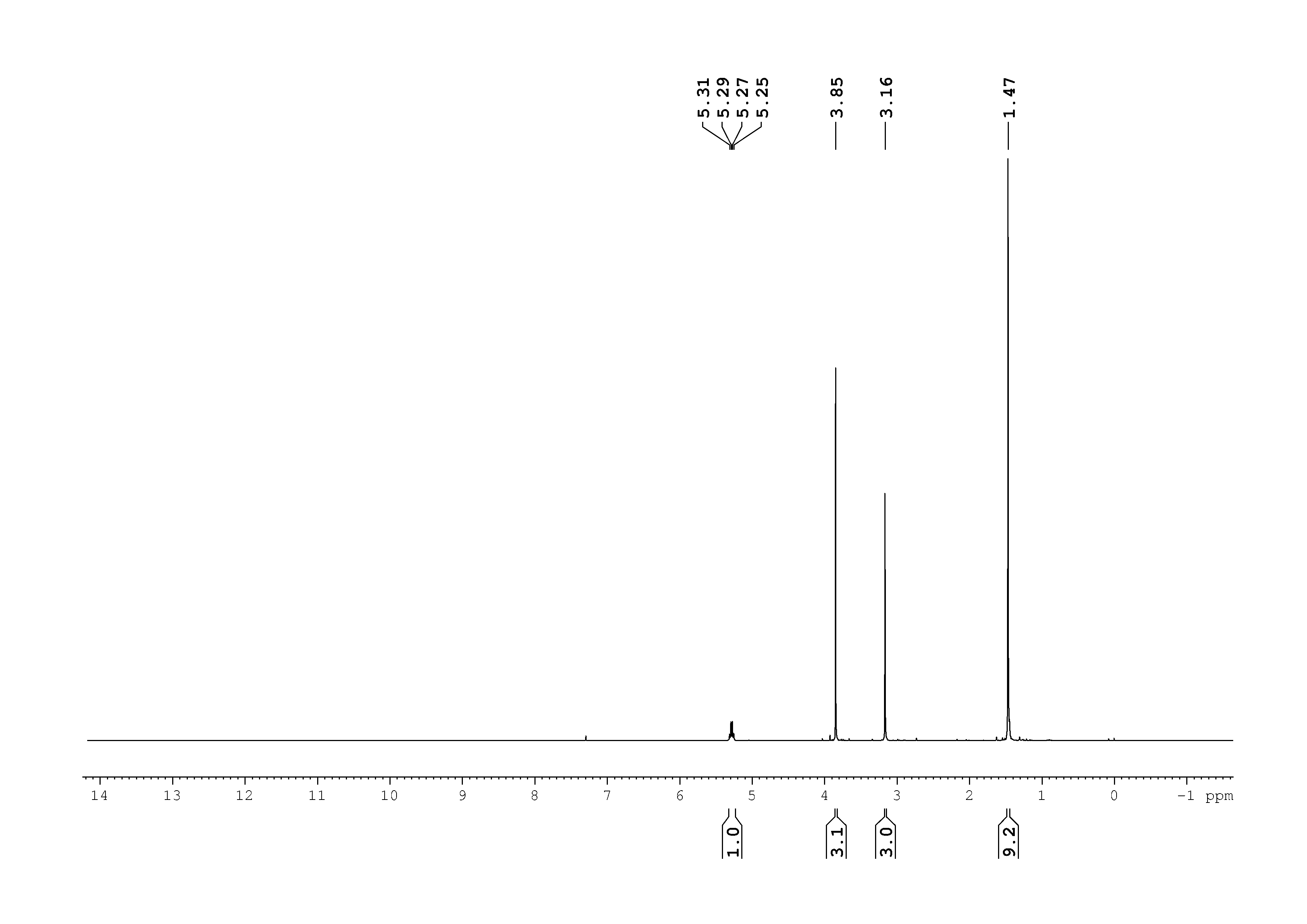
^1^H-NMR spectra of compound **3n**, CDCl_3_, 400.1 MHz


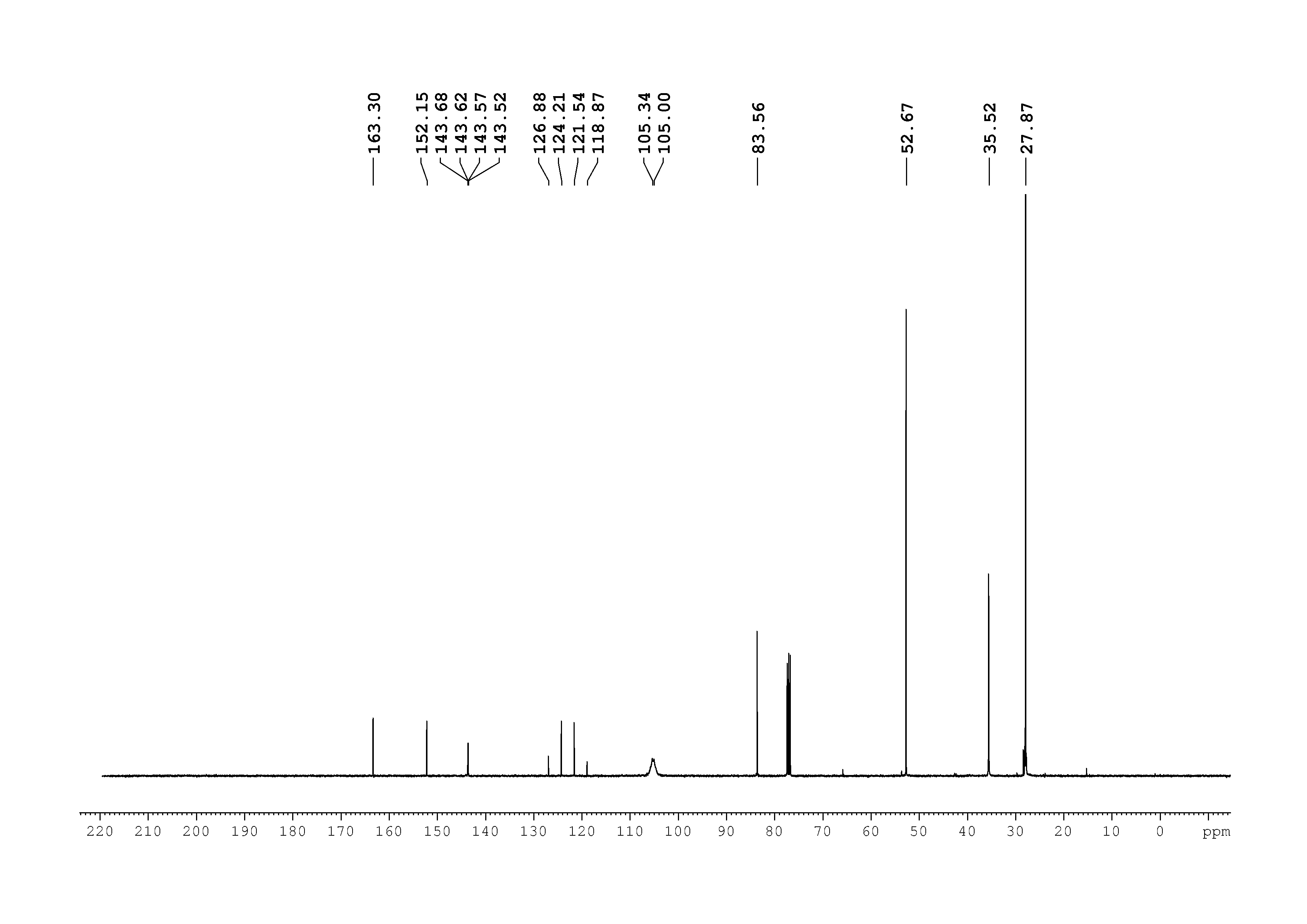
^13^C-NMR spectra of compound **3n**, CDCl_3_, 100.6 MHz


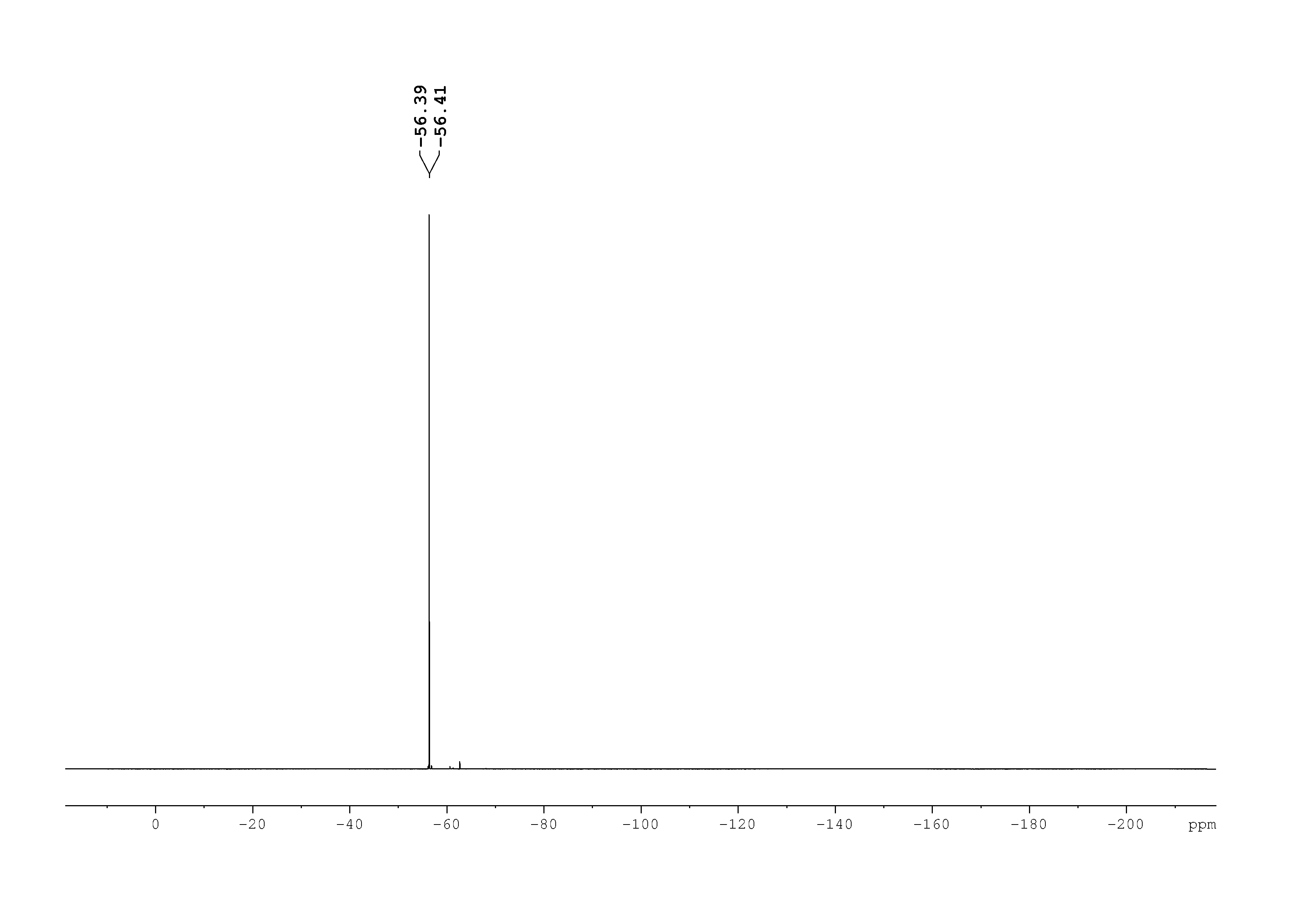
^19^F-NMR spectra of compound **3n**, CDCl_3_, 376.5 MHz


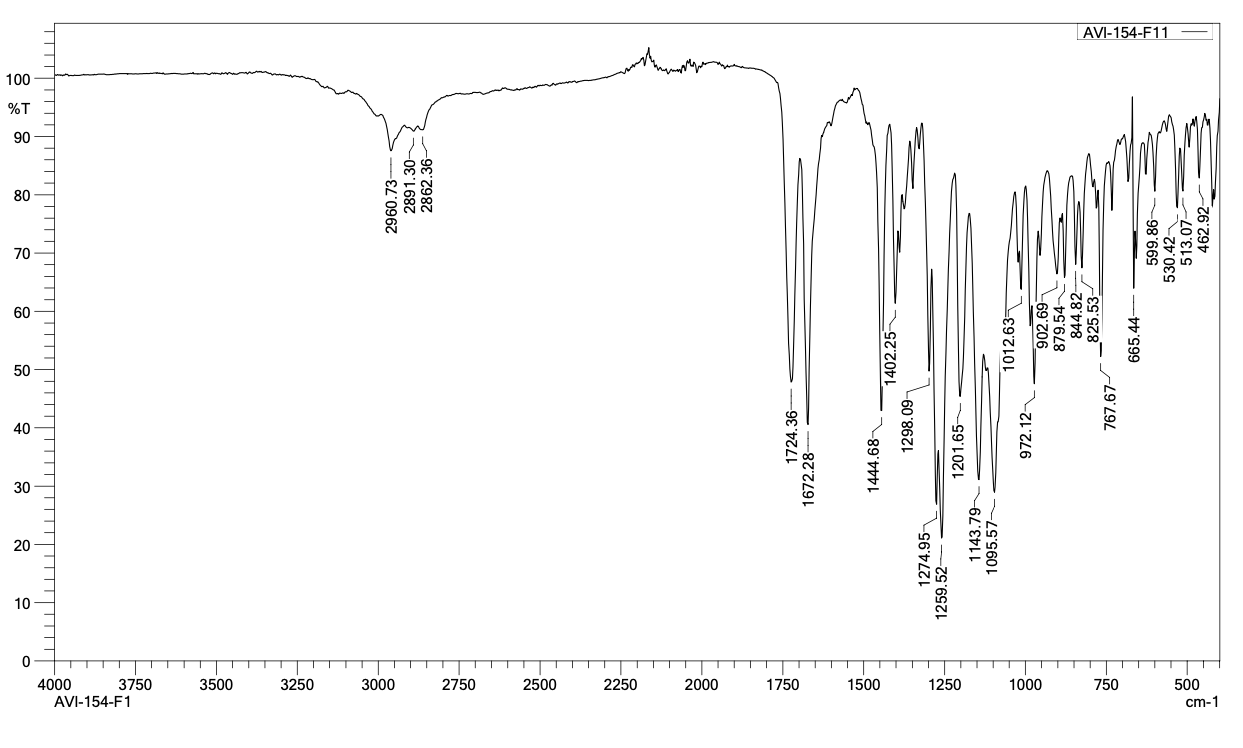
FTIR spectra of compound **3c**


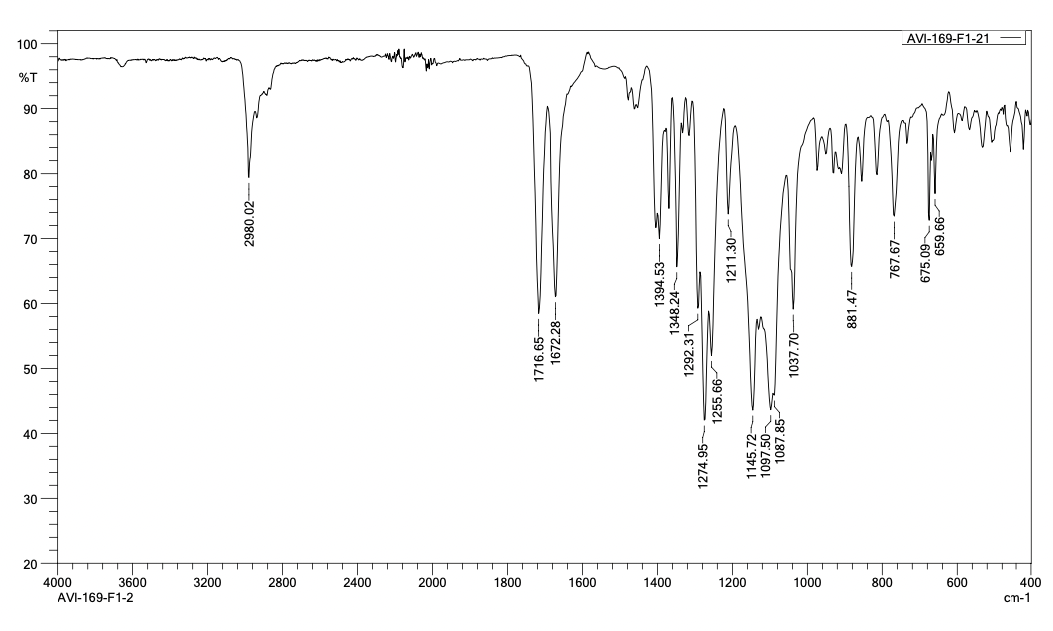
FTIR spectrum of compound **3d**

FTIR spectra of compound **3e**


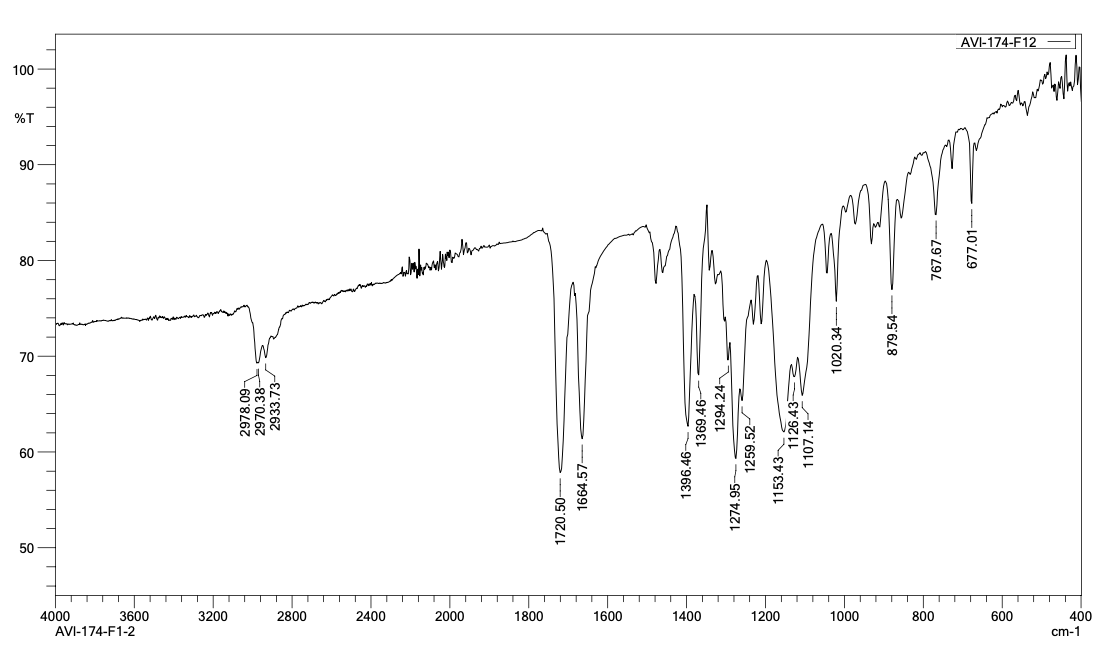


FTIR spectra of compound **3f**


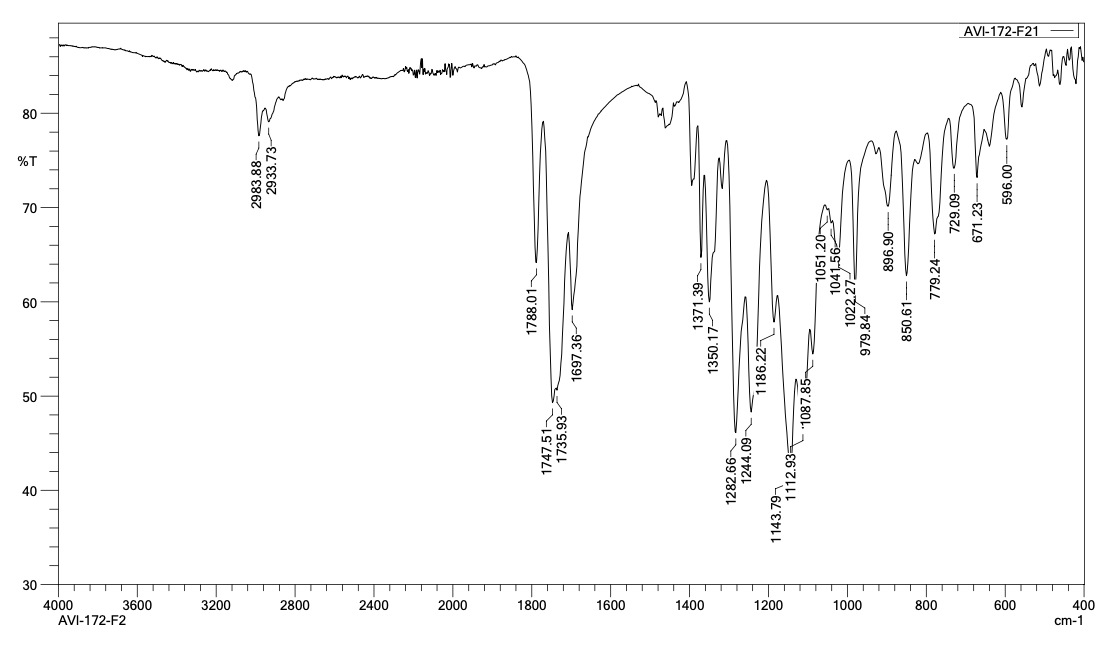


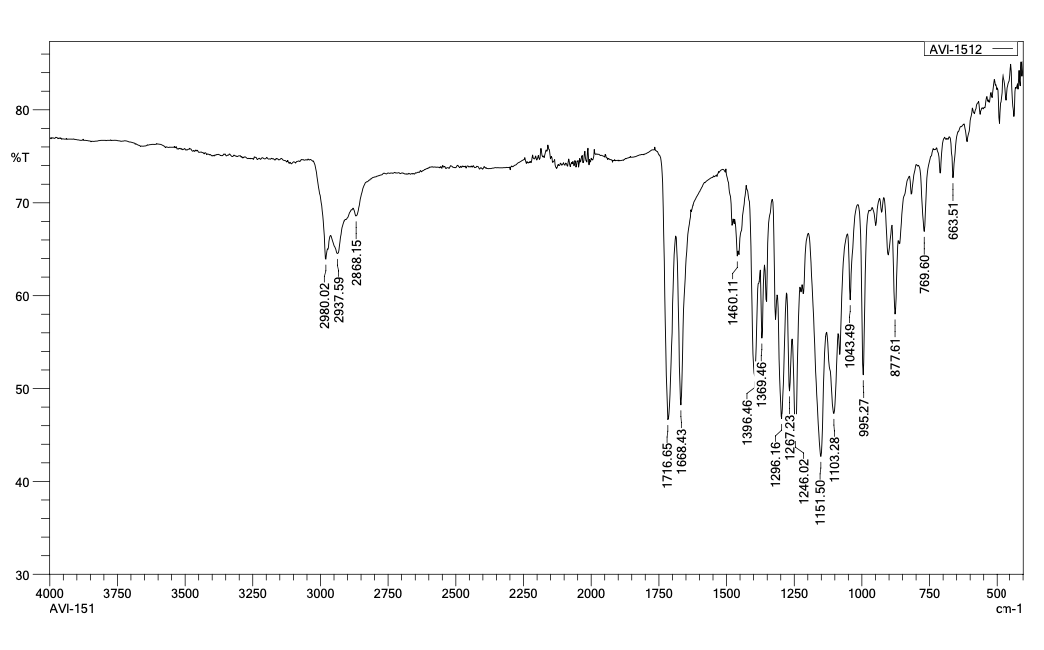
FTIR spectra of compound **3g**

FTIR spectra of compound **3h**


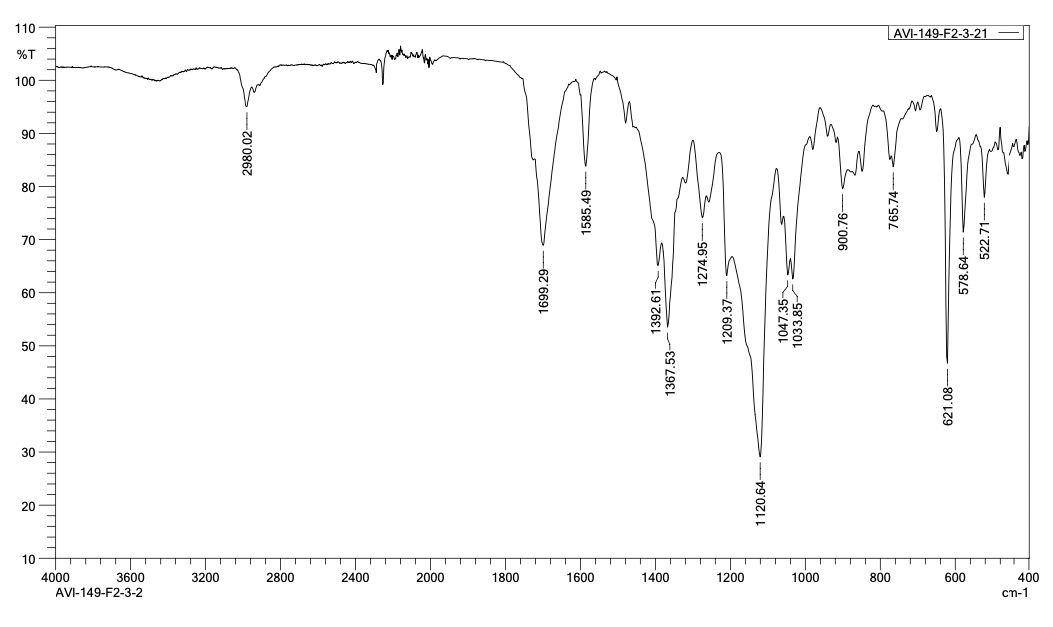


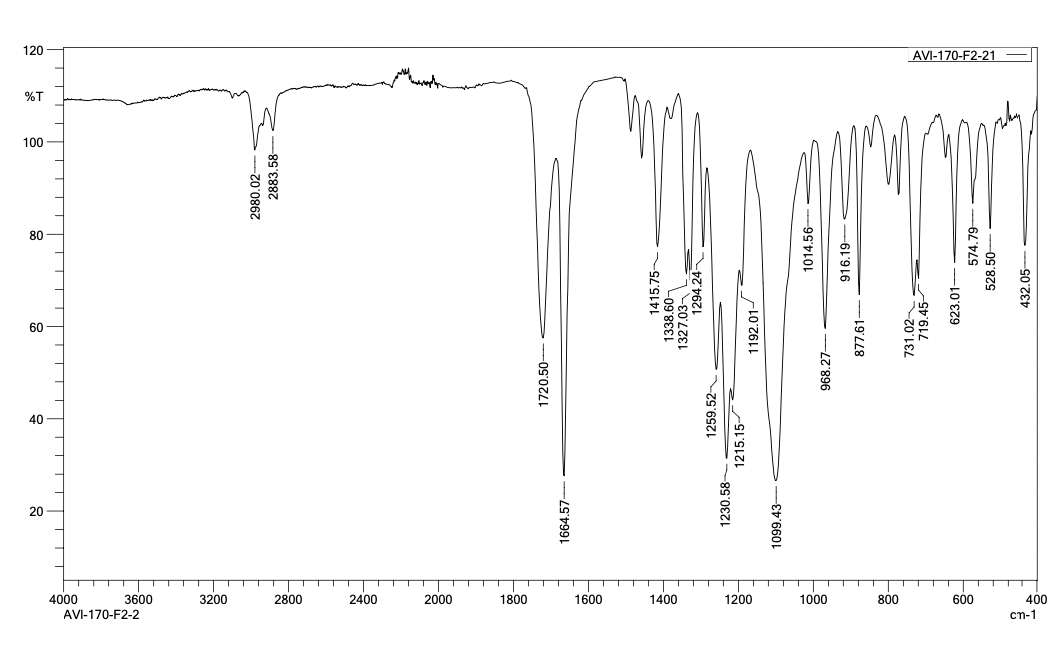
FTIR spectra of compound **3k**

FTIR spectra of compound **3m**


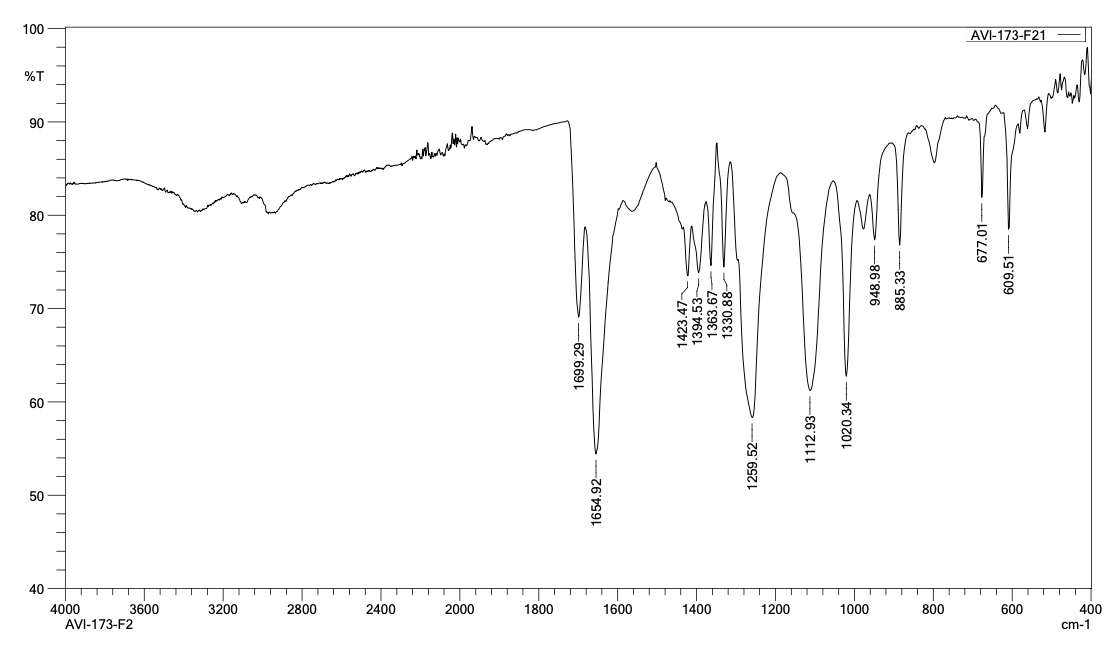


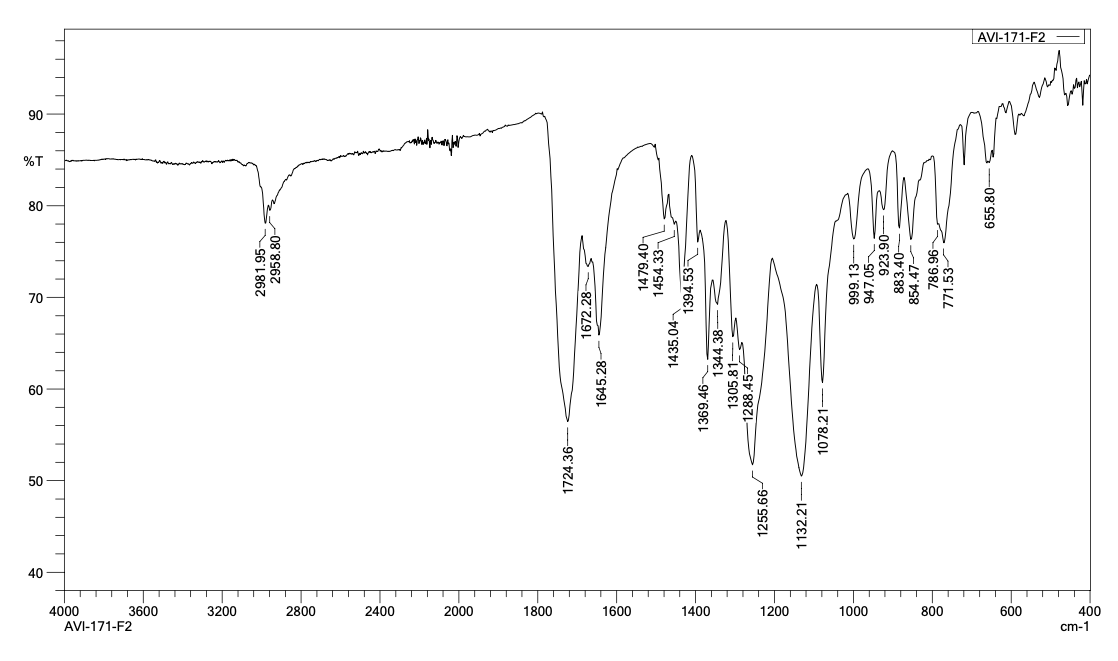
FTIR spectra of compound **3n**
